# Synthesis of tryptophan-dehydrobutyrine diketopiperazine and biological activity of hangtaimycin and its co-metabolites

**DOI:** 10.3762/bjoc.18.120

**Published:** 2022-09-07

**Authors:** Houchao Xu, Anne Wochele, Minghe Luo, Gregor Schnakenburg, Yuhui Sun, Heike Brötz-Oesterhelt, Jeroen S Dickschat

**Affiliations:** 1 Kekulé-Institute of Organic Chemistry and Biochemistry, University of Bonn, Gerhard-Domagk-Straße 1, 53121 Bonn, Germany; 2 Interfaculty Institute of Microbiology and Infection Medicine, Department of Microbial Bioactive Compounds, University of Tübingen, Auf der Morgenstelle 28, 72076 Tübingen, Germany; 3 Key Laboratory of Combinatorial Biosynthesis and Drug Discovery, Ministry of Education, and School of Pharmaceutical Sciences, Wuhan University, No. 185 East Lake Road, Wuhan, 430071, People's Republic of China; 4 Institute of Inorganic Chemistry, University of Bonn, Gerhard-Domagk-Straße 1, 53121 Bonn, Germany

**Keywords:** antibiotics, enantioselective synthesis, peptides, racemisation, *Streptomyces*

## Abstract

An improved synthesis for tryptophan-dehydrobutyrine diketopiperazine (TDD), a co-metabolite of the hybrid polyketide/non-ribosomal peptide hangtaimycin, starting from ʟ-tryptophan is presented. Comparison to TDD isolated from the hangtaimycin producer *Streptomyces spectabilis* confirmed its *S* configuration. The X-ray structure of the racemate shows an interesting dimerisation through hydrogen bridges. The results from bioactivity testings of hangtaimycin, TDD and the hangtaimycin degradation product HTM_222_ are given.

## Introduction

**Scheme 1:**
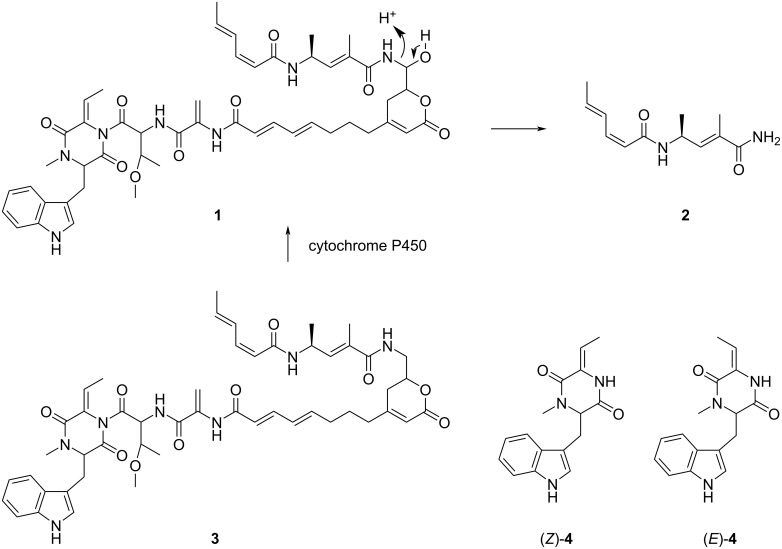
Structures of hangtaimycin (**1**) and its co-metabolites.

Hangtaimycin (**1**, Scheme 1) was first isolated from *Streptomyces spectabilis* and shown to possess weak antimicrobial activity against *Bacillus subtilis* [1]. Together with a structural revision from 29*Z* to 29*E* configuration and further biological evaluation of its hepatoprotective properties, its biosynthetic gene cluster was recently identified [2]. The biosynthetic machinery is composed of a hybrid *trans*-acyltransferase (*trans*-AT, [3–4]) polyketide synthase (PKS) and non-ribosomal peptide synthase (NRPS) [2] with a dehydrating bimodule [5–6] involved in the installation of the remaining *Z*-configured double bond within the polyketide backbone [7]. Furthermore, a cytochrome P450 monooxygenase was recently shown to be responsible for the oxidation of deoxyhangtaimycin (**3**), a compound with antiviral activity, to **1** [8]. The thereby installed hemiaminal function is also the breaking point for **1** into a larger lactone-polyene peptide fragment and a smaller fragment HTM_222_ (**2**, named after its molecular mass of 222 Da) [2]. Another hangtaimycin co-metabolite in *S. spectabilis* [9] is tryptophan-dehydrobutyrine diketopiperazine (TDD, **4**) that was already isolated several decades before the discovery of **1**, and likewise reported to have no antibacterial activity [9]. The initially published structure was that of (*E*)-**4** [9], but later revised as that of (*Z*)-**4**. The same compound is also observed in *S. olivaceus* [10] and was reported to function as a competitive inhibitor of glutathione *S*-transferase [11], which may be a result of a thiol addition of glutathione to the Michael acceptor in **4**. While the relative and absolute configuration of hangtaimycin have not yet firmly been established, **2** is known to be *S*-configured and is derived from an ʟ-alanine unit [2]. TDD (**4**) was recently suggested to be *R*-configured, containing a ᴅ-tryptophan unit, based on a comparison of the optical rotation of the isolated compound ([α]_D_^20^ = −12.67, *c* 1.1, 95% EtOH [1]) to **4** synthesised from ʟ-tryptophan ([α]_D_^21^ = +13, *c* 0.03, EtOH [12]), but the melting point of the synthetic material (mp 191–192 °C, for the compound numbered (*Z*)-**32** in ref. [12]) did not match that of isolated TDD (mp 121–123 °C [9]), and conclusively the compounds that have been compared cannot be the same. This prompted the authors of the synthetic study to conclude on the need for a structural revision of **4** [12], with unclear reasoning for the newly assigned structure. However, this newly suggested structure of **4** is not reflected in the structure of **1** [1–2] and not supported by bioinformatic analysis of its biosynthetic gene cluster [2], although it seems reasonable to consider **4** as a degradation product of **1**. Moreover, the originally reported optical rotation of **4** is positive ([α]_D_^24.5^ = +10.0, *c* 1.1, 95% EtOH [9]), in contrast to the later reported negative value mentioned above [1]. In order to resolve the confusion, we have reisolated **4** from *S. spectabilis* and report on an improved synthesis. Furthermore, the results from bioactivity testings with **1**, **2** and **4** are discussed.

## Results and Discussion

### Synthesis of TDD

**Scheme 2:**
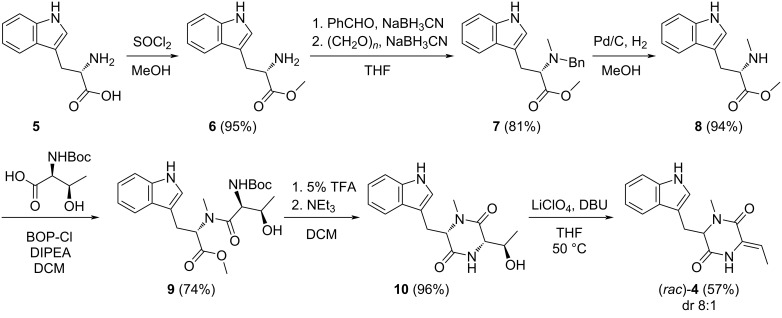
First synthetic route towards TDD (**4**).

**Figure 1:**
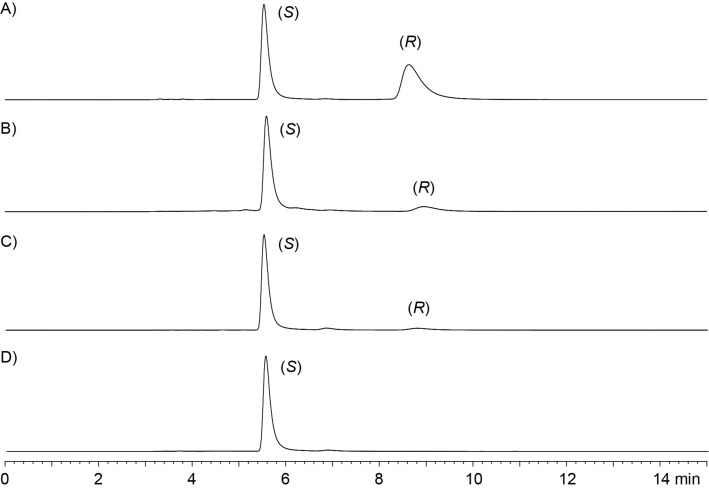
HPLC analyses of (*Z*)-**4** on a chiral stationary phase. A) Nearly racemic **4** from the first synthetic route (Scheme 2), B) enantiomerically enriched **4** (80% ee) from the second synthetic approach (Scheme 3), C) enantiomerically enriched **4** (90% ee) obtained under optimised conditions for the elimination reaction of **14** (Scheme 3), and D) natural enantiomerically pure (*S*)-TDD isolated from *S. spectabilis*.

The first synthetic route towards **4** started from ʟ-tryptophan (**5**) that was converted through a standard transformation into the methyl ester **6** and then through sequential reductive aminations with benzaldehyde and paraformaldehyde into **7** (Scheme 2) [13]. Cleavage of the benzyl group by catalytic hydrogenation afforded **8** that was coupled with *tert*-butyloxycarbonyl (Boc)-protected threonine using bis(2-oxo-3-oxazolidinyl)phosphinic chloride (BOP-Cl) [14–15] and Hünig’s base to give **9**. Cleavage of the Boc group with 5% TFA followed by basic treatment resulted in the cyclisation to the dioxopiperazine **10**. Acetylation and subsequent treatment with LiClO_4_ and DBU is a common strategy for the dehydration of serine and threonine units in peptides [16], but unfortunately the acetylation of **10** failed. Interestingly, the direct treatment of **10** with LiClO_4_ and DBU under prolonged reaction times (3 days) resulted in the elimination of water. This reaction proceeded with a high diastereoselectivity (*Z*/*E* = 8:1), giving access to **4** in a satisfactory yield of 29% over 6 steps. However, the optical rotation of the obtained material showed only a small positive value ([α]_D_^25^ = +1.9, *c* 0.27, EtOH), suggesting that **4** had undergone racemisation during the prolonged basic treatment with DBU in the last step. This was confirmed by HPLC analysis on a chiral stationary phase, showing that the obtained target compound **4** was nearly racemic (Figure 1A).

**Scheme 3:**
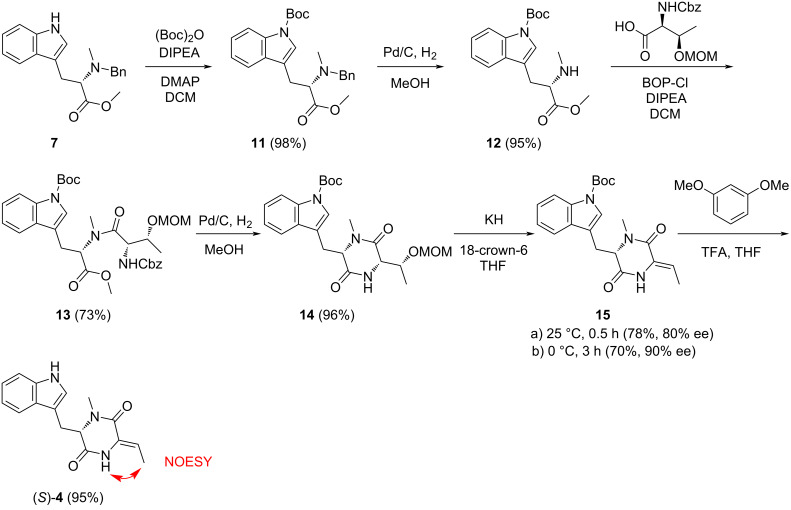
Second synthetic route towards TDD ((*Z*)-**4**).

Because of the configurational instability of **4** under base treatment, we aimed at an approach for the final elimination step using milder conditions (Scheme 3). The newly developed synthesis started from **7** that was Boc-protected at the indole to yield **11**. Removal of the benzyl group by catalytic hydrogenation to **12** was followed by coupling with benzyloxycarbonyl (Cbz) and methoxymethyl (MOM)-protected threonine to give **13**. Removal of the Cbz group by catalytic hydrogenation proceeded with spontaneous cyclisation to **14**. With this material, the elimination of the MOM group smoothly proceeded by treatment with KH and 18-crown-6 in THF at 25 °C to **15**, that upon removal of the Boc group with TFA and 1,3-dimethoxybenzene [17] gave (*Z*)-**4** as a single diastereomer through *anti* elimination. Overall, TDD was obtained from ʟ-tryptophan in a high yield of 37% over eight linear steps. HPLC analysis on a chiral stationary phase showed that **4** obtained through this second route was enantiomerically enriched (80% ee by peak integration, Figure 1B). Further improvement of the enantiomeric excess of **4** (90% ee, Figure 1C) was possible by performing the elimination reaction with **14** and KH and 18-crown-6 under ice cooling. This helped to suppress basic racemisation of **4**, but required prolonged reaction times and gave a slightly diminished yield for **15** (70%), lowering the overall yield of TDD to 33% over eight steps. The major enantiomer of **4** obtained from this second route was identical to natural TDD (Figure 1D) which is thus *S*-configured, i.e., derived from ʟ-tryptophan. Moreover, the olefinic double bond in **4** is *Z*-configured as indicated by a strong NOESY correlation between the amide NH and the neighbouring methyl group.

### The structure of TDD

**Figure 2:**
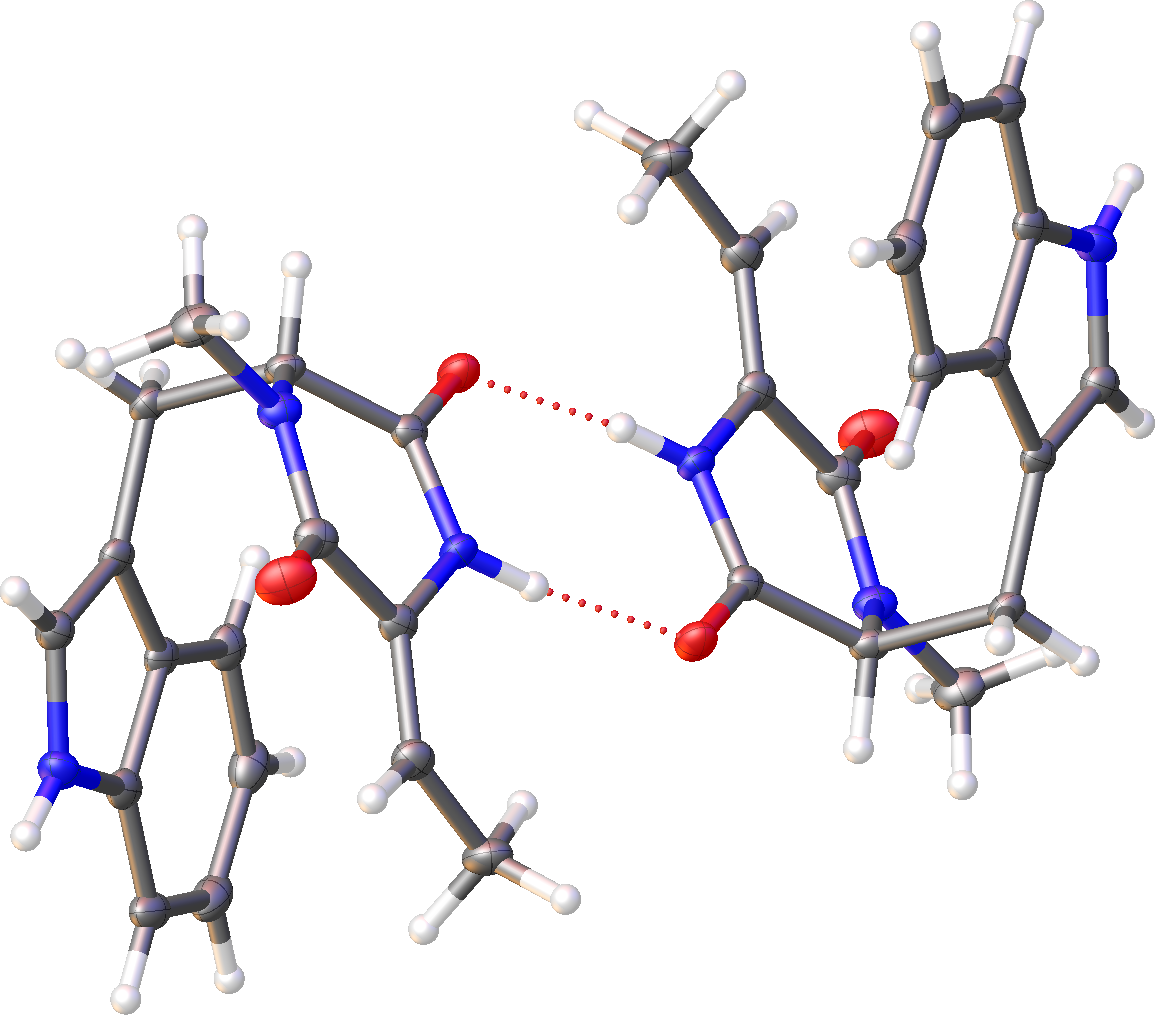
X-ray structure of (*rac*)-**4**.

These findings not only challenge the originally assigned structure of (*E*)-**4** [9] and confirm the structural revision of (*Z*)-**4** [1], but also question the suggested structural revision that placed the *N*-methyl group at the other nitrogen of the dioxopiperazine moiety [12]. Moreover, the confusing situation about the absolute configuration and optical rotation are resolved through this work, clearly showing *S*-configuration for **4** that exhibits a negative optical rotation ([α]_D_^25^ = –15.5, *c* 0.102, MeOH). The reason for the varying melting points for **4** in the literature is unclear, but we noticed a pronounced difference in the crystallisation behaviour of racemic and enantiomerically pure **4**. While (*rac*)-**4** readily formed crystals (mp 134–136 °C), several attempts to crystallise (*S*)-**4**, a material that was obtained as a viscous oil, failed. The X-ray crystallographic analysis of (*rac*)-**4** showed an interesting dimer interaction of its enantiomers through hydrogen bridges between the amide (NH-CO) groups (Figure 2, for crystallographic parameters cf. Supporting Information File 1, Table S1), that may support its easy crystallisation in comparison to enantiomerically enriched **4**. Note that the dimer between the two enantiomers of **4** is achiral which allows for a regular packing of (*rac*)-**4** in the crystal. In contrast, a hypothetical similar interaction between two molecules of the same enantiomer can only lead to a chiral dimer that, if formed at all, may crystallise less efficiently.

### Bioactivity testing

**Figure 3:**
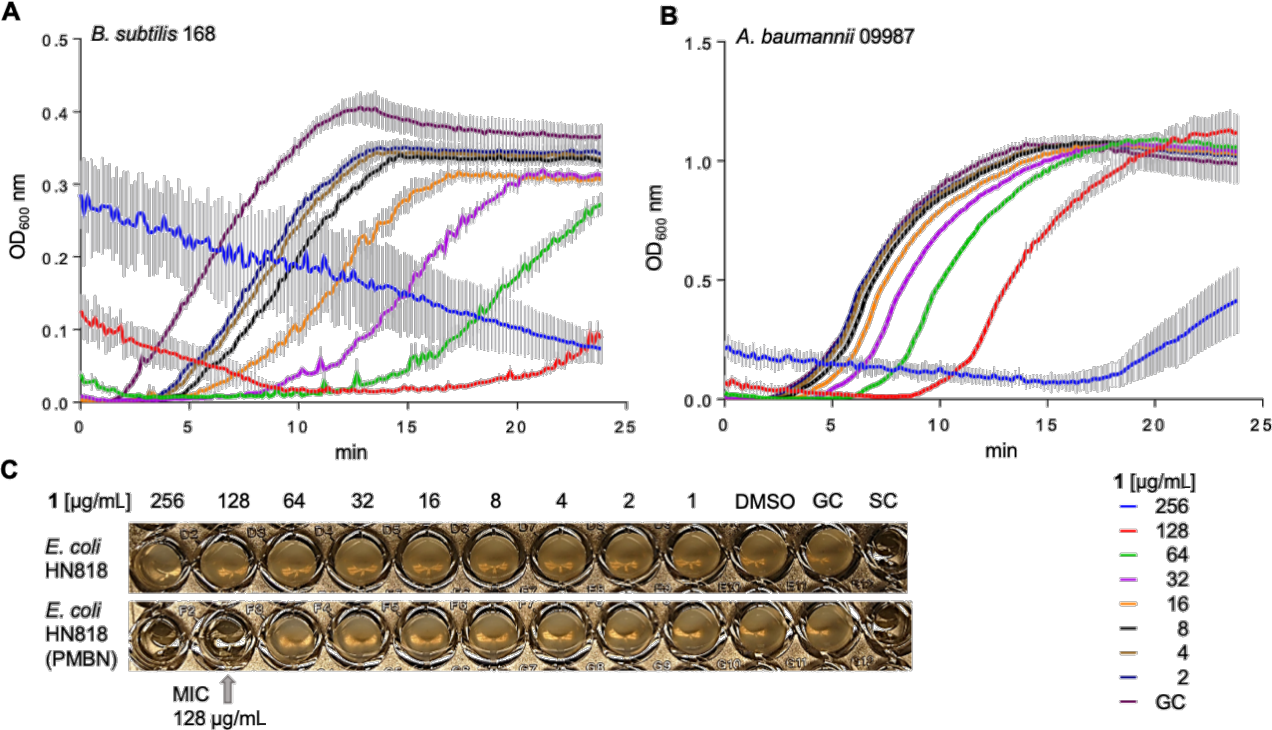
Bioactivity testing with hangtaimycin (**1**). A) Growth retardation of model species *B. subtilis* 168 and of B) nosocomial pathogen *A. baumannii* 09987 monitored by recording the turbidity increase of growing culture aliquots exposed to a concentration series of **1** every 10 min over 24 h. An increase in the optical density at 600 nm (OD_600_) reflects biomass production. At concentrations of ≥64 µg/mL **1** precipitated, leading to elevated OD_600_ values at the beginning of the experiment that were unrelated to growth. The curves reflect the mean values of three separate cultures and standard deviations are depicted by black bars. C) MIC assay against *E. coli*. The MIC is defined as the lowest concentration of an antibacterial agent inhibiting visible bacterial growth (no turbidity detected by the naked eye) after overnight incubation. Only when the outer membrane was permeabilised by polymyxin B nonapeptide (PMBN), **1** inhibited growth of *E. coli* sufficiently to yield a clear MIC. GC, growth control (no inhibitor), SC, sterile control (no bacteria), DMSO, culture medium supplemented with 1% DMSO, reflecting the DMSO concentrations in the hangtaimycin-containing samples.

Previous reports have mentioned that TDD (**4**) exhibits no antibacterial activity, without providing information about the test organisms used [9]. For this reason, and because of the above-mentioned confusions about the true nature of **4** in the previous literature, the bioactivity of natural (enantiomerically pure) **4** isolated from *S. spectabilis* was reinvestigated. For comparison, synthetic (*rac*)-(*Z*)-**4** and its stereoisomer (*rac*)-(*E*)-**4** (Scheme 2) were included in the bioactivity testing, as well as the previously synthesised HTM_222_ (**2**) and **1** isolated from *S. spectabilis*. Neither **2** nor any of the stereoisomers of **4** showed antibacterial effects against a panel of Gram-positive and Gram-negative organisms (Supporting Information File 1, Table S2). Only **1** exhibited concentration-dependent growth retardation of the Gram-positive species *Bacillus subtilis* 168 and *Acinetobacter baumannii* 09987 (Figure 3A and B). However, growth inhibition was not strong enough to yield a clear MIC value, as the determination of a MIC requires complete inhibition of visible bacterial growth and residual growth occurred up to the highest concentration tested (256 µg/mL). In the Gram-negative *Escherichia coli*, the outer membrane protects the cells from the impact of **1**. When the integrity of the outer membrane was compromised by adding the outer-membrane permeabilizing polymyxin B nonapeptide (PMBN, 10 μg/mL), a MIC of 128 mg/mL was achieved (Figure 3C).

We also investigated whether the reported inhibition of glutathione *S*-transferase [11] is a result of a Michael addition of glutathione to TDD. However, no reaction occurred between glutathione and TDD in DMF/H_2_O (1:1) under prolonged stirring at room temperature. Also the addition of base (NEt_3_) did not promote the reaction. Therefore, the mode of action of TDD towards glutathione *S*-transferase needs further investigation.

## Conclusion

We have established an efficient synthesis of TDD that makes this compound available from ʟ-tryptophan with a high yield of 33% (90% ee) over eight linear steps, establishing *S* configuration for the natural product from *S. spectabilis* that is likely reflected in the corresponding portion of hangtaimycin. A key step in the synthesis is the elimination of a MOM group using KH and 18-crown-6 that must be carried out with care, because TDD easily undergoes racemisation under basic conditions. The X-ray analysis showed an interesting dimer interaction of the enantiomers in racemic TDD through hydrogen bridges, that may support its much easier crystallisation in comparison to enantiomerically pure or enriched TDD. In fact, the obtained viscous oils did not crystallise, suggesting that the previously reported melting point of 121–123 °C [9] that is close to our measured melting point for (*rac*)-**4** (134–136 °C) may have been measured for isolated material after it had undergone (partial) racemisation. The reported inactivity of **4** against bacteria was confirmed in this study, and also **2** is an inactive metabolite of *S. spectabilis*, while for **1** moderate growth retardation against *A. baumannii* and *B. subtilis*, and growth inhibition against PMBN-treated *E. coli* was observed. However, the low activity of **1** in these assays suggests that the natural function of this structurally remarkable compound awaits future clarification.

## Supporting Information

File 1 Experimental, analytical and X-ray data as well as copies of NMR spectra.
