# Supplementary material for: Synthesis of tryptophan-dehydrobutyrine diketopiperazine and biological activity of hangtaimycin and its co-metabolites
Source: Beilstein J Org Chem. 2022 Sep 7;18:1159–65. doi: 10.3762/bjoc.18.120 (PMC9475180; doi:10.3762/bjoc.18.120)
Supplement: File 1 — Experimental, analytical and X-ray data as well as copies of NMR spectra. [file Beilstein_J_Org_Chem-18-1159-s001.pdf]

BEILSTEIN JOURNAL OF ORGANIC CHEMISTRY

Supporting Information

for

Synthesis of tryptophan-dehydrobutyrine diketopiperazine and biological activity of hangtaimycin and its co-metabolites

Houchao Xu, Anne Wochele, Minghe Luo, Gregor Schnakenburg, Yuhui Sun,
Heike Brötz-Oesterhelt and Jeroen S. Dickschat

Beilstein J. Org. Chem. **2022,** *18,* 1159–1165. doi:10.3762/bjoc.18.120

Experimental, analytical and X-ray data as well as copies of NMR spectra

License and Terms: This is a supporting information file under the terms of the Creative Commons Attribution License (https://creativecommons.org/licenses/by/4.0). Please note that the reuse, redistribution and reproduction in particular requires that the author(s) and source are credited and that individual graphics may be subject to special legal provisions.
The license is subject to the *Beilstein Journal of Organic Chemistry* terms and conditions: (https://www.beilstein-journals.org/bjoc/terms)

General synthetic and analytical methods

Chemicals were obtained from Sigma Aldrich (St. Louis, MO, USA), Thermo Fisher Scientific (Waltham, MA, USA) and TCI (Tokyo, Japan) and were used without further purification. Solvents for reactions were dried according to standard procedures. POLYGRAM® SIL G/UV254 plates (Macherey-Nagel, Düren, Germany) were used for TLC. The staining solution for TLC was molybdophosphoric acid in EtOH (10 g/100 mL). The solvents used for purification were distilled before use. Flash chromatography was performed with silica Geduran® Si 60 (40–63 µm, Merck, Darmstadt, Germany). NMR spectra were recorded on a Bruker Avance I 500 MHz spectrometer or a Bruker Avance III HD 700 MHz Cryo spectrometer. Chemical shifts were referenced to the residual proton signal of the solvent for ^{1}H NMR and the ^{13}C signal for ^{13}C NMR. IR spectra were recorded on a Bruker α infrared spectrometer with a diamond ATR probehead. Peak intensities are given as s (strong), m (medium), w (weak) and br (broad). Optical rotations were recorded on a Modular Compact Polarimeter MCP 100 (Anton Paar, Graz, Austria). The optical rotation parameters are 1) temperature: 25 °C, 2) wavelength: 589 nm (the sodium D line), and 3) the path-length: 10 cm. Concentrations *c* are given in g 100 mL^{-1}. High resolution mass spectra were measured with an LTQ Orbitrap XL (Thermo Scientific, Waltham, Massachusetts, USA).

Synthetic procedures

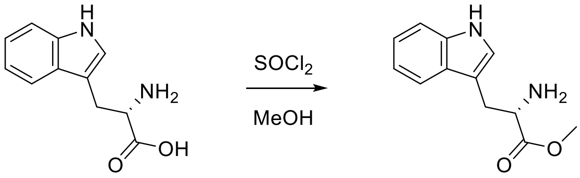

5 **6**

$SOCl_2$ (4.45 mL, 61.3 mmol) was added to MeOH (80 mL) dropwise at 0 °C. The mixture was stirred at 0 °C for 30 min, followed by the addition of **5** (5.00 g, 24.5 mmol). After refluxing for 18 h, the solvent was evaporated and sat. aq. Na_2CO_3 solution (100 mL) was added. The aqueous phase was extracted with EtOAc (3 × 100 mL). The combined organic phases were dried with $MgSO_4$ and then concentrated under reduced pressure to give **6** as white solid.

Methyl L-tryptophanate (6). Yield: 5.07 g, 23.3 mmol (95%); **^{1}H-NMR** (400 MHz, CD_3OD): δ (ppm) 7.55 (d, J = 8.0 Hz, 1H), 7.37 (d, J = 8.1 Hz, 1H), 7.12 (m, 2H), 7.04 (ddd, J = 8.0, 7.0, 1.1 Hz, 1H), 3.79 (dd, J = 6.7, 5.7 Hz, 1H), 3.67 (s, 3H), 3.22 (ddd, J = 14.3, 5.7, 0.8 Hz, 1H), 3.13 (ddd, J = 14.3, 6.7, 0.7 Hz, 1H); **^{13}C-NMR** (100 MHz, CD_3OD): δ (ppm) 176.7 (C), 138.1 (C), 128.7 (C), 124.6 (CH), 122.5 (CH), 119.8 (CH), 119.2 (CH), 112.3 (CH), 110.6 (C), 55.9 (CH), 52.4 (CH_3), 31.4 (CH_2).

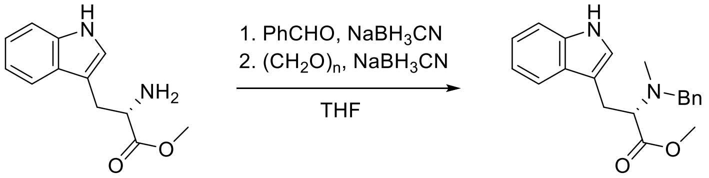

6 **7**

Compound **6** (2.18 g, 10.0 mmol) was added to MeOH (100 mL), followed by benzaldehyde (1.11 g, 10.5 mmol). After stirring at room temperature for 1 h, $NaBH_3CN$ (0.66 g, 10.5 mmol) was added and CH_3COOH was used to adjust the pH of the reaction solution until pH 6–7 was reached. The reaction mixture was then stirred at room temperature for 18 h, followed by the addition of paraformaldehyde (0.30 g, 10.0 mmol) and $NaBH_3CN$ (0.66 g, 10.5 mmol). CH_3COOH was again added dropwise until pH 6–7 was reached. After stirring for another 18 h, the reaction mixture was concentrated under reduced pressure and added with sat. aq. $NaHCO_3$ solution (100 mL). The aqueous phase was extracted with EtOAc (3 × 100 mL). The combined organic phases were dried with $MgSO_4$. After removal of the solvent under reduced

pressure, the residue was purified by column chromatography on silica gel (cyclohexane : EtOAc = 3 : 1) to give known compound **7** [1] as colorless oil.

Methyl *N*$^{\alpha}$-benzyl-*N*$^{\alpha}$-methyl-L-tryptophanate (7). TLC (cyclohexane : EtOAc = 3 : 1): R_f = 0.34; **Yield**: 2.60 g, 8.1 mmol (81%); **Optical rotation**: $[\alpha]_D^{25}$ = –48.9 (*c* 0.18, MeOH); **^{1}H-NMR** (400 MHz, CD_3OD): δ (ppm) 7.38 (d, *J* = 7.9 Hz, 1H), 7.32 (d, *J* = 8.2 Hz, 1H), 7.26 (m, 5H), 7.08 (ddd, *J* = 8.2, 7.0, 1.1 Hz, 2H), 7.00 (d, *J* = 0.8 Hz, 1H), 6.96 (ddd, *J* = 8.0, 7.0, 1.0 Hz, 1H), 3.82 (d, *J* = 13.3 Hz, 1H), 3.72 (dd, *J* = 9.5, 5.6 Hz, 1H), 3.60 (m, 4H), 3.30 (dd, *J* = 4.6, 0.7 Hz, 1H), 3.08 (ddd, *J* = 14.2, 5.6, 0.9 Hz, 1H); **^{13}C-NMR** (101 MHz, CD_3OD): δ (ppm) 174.1 (C), 140.2 (C), 138.0 (C), 130.1 (CH), 129.2 (CH), 128.7 (C), 128.1 (CH), 124.1 (CH), 122.2 (CH), 119.6 (CH), 119.2 (CH), 112.2 (CH), 111.9 (C), 67.6 (CH), 60.1 (CH_2), 51.4 (CH_3), 38.5 (CH_3), 26.8 (CH_2).

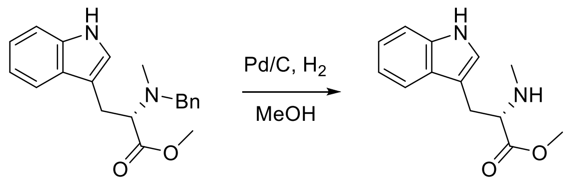

7 8

A solution of **7** (2.19 g, 6.8 mmol) in MeOH (20 mL) was added to palladium on charcoal (5 wt %, 0.22 g, 10 % w/w) under argon atmosphere. The reaction mixture was stirred under the pressure of H_2 (4 bar) overnight. After filtration, the residue was concentrated under reduced pressure to give known compound **8** [1] as pale yellow oil without further purification.

Methyl *N*$^{\alpha}$-methyl-L-tryptophanate. TLC (EtOAc : MeOH = 10 : 1): R_f = 0.23; **Yield**: 1.48 g, 6.4 mmol (94%); **Optical rotation**: $[\alpha]_D^{25}$ = 26.0 (*c* 0.10, MeOH); **^{1}H-NMR** (500 MHz, CD_3OD): δ (ppm) 7.53 (d, *J* = 7.9 Hz, 1H), 7.35 (d, *J* = 8.1 Hz, 1H), 7.11 (ddd, *J* = 8.2, 7.1, 1.2 Hz, 1H), 7.08 (s, 1H), 7.03 (ddd, *J* = 8.0, 7.0, 1.1 Hz, 1H), 3.60 (m, 4H), 3.17 (dd, *J* = 6.6, 0.8 Hz, 2H), 2.36 (s, 3H); **^{13}C-NMR** (126 MHz, CD_3OD): δ (ppm) 175.5 (C), 138.1 (C), 128.6 (C), 124.5 (CH), 122.5 (CH), 119.8 (CH), 119.1 (CH), 112.3 (CH), 110.4 (C), 64.9 (CH), 52.2 (CH_3), 34.4 (CH_3), 29.5 (CH_2).

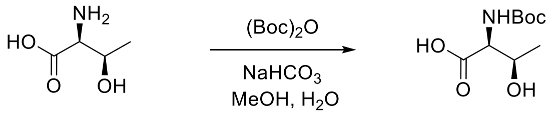

To a solution of L-threonine (**S1**) (2.00 g, 16.8 mmol) in H_2O (34 mL) was added $NaHCO_3$ (2.17 g, 25.9 mmol), followed by $(Boc)_2O$ (5.72 g, 26.2 mmol) dissolved in MeOH (34 mL). After the reaction mixture was stirred at room temperature for 24 h, MeOH was removed in vacuum. The residue was acidified with aq. HCl solution (2 N) until the pH = 2 was reached. The aqueous phase then was extracted with EtOAc (3 × 100 mL). The combined organic phases were dried with $MgSO_4$ and concentrated under reduced pressure to give **S2** as colorless oil.

(*tert*-Butoxycarbonyl)-L-threonine. Yield: 3.64 g, 16.6 mmol (99%); **^{1}H-NMR** (500 MHz, CD_3OD): δ (ppm) 4.24 (qd, J = 6.4, 2.9 Hz, 1H), 4.06 (d, J = 2.9 Hz, 1H), 1.43 (s, 9H), 1.18 (d, J = 6.4 Hz, 3H); **^{13}C-NMR** (126 MHz, CD_3OD): δ (ppm) 174.4 (C), 158.3 (C), 80.7 (C), 68.6 (CH), 60.4 (CH), 28.7 (CH_3), 20.4 (CH_3).

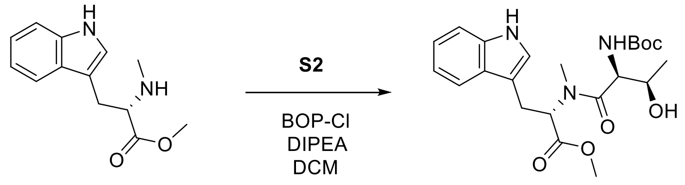

Compound **S2** (1.10 g, 5.0 mmol) was added to a solution of **8** (1.16 g, 5.0 mmol) and *N*,*N*-diisopropylethylamine (2.09 mL, 12.0 mmol) in CH_2Cl_2 (40 mL). After the mixture was cooled to 0 °C BOP-Cl (1.40 g, 5.5 mmol) was added. The reaction solution was stirred at room temperature for 15 h and then diluted with EtOAc (200 mL). After washing with sat. aq. NH_4Cl solution (30 mL), sat. aq. $NaHCO_3$ solution (30 mL) and brine (30 mL), the organic phase was dried with $MgSO_4$ and concentrated in vacuum. The residue was purified by column chromatography on silica gel (EtOAc) to give **9** as yellowish oil.

Methyl N^{α}-((*tert*-butoxycarbonyl)-L-threonyl)-N^{α}-methyl-L-tryptophanate (9). TLC (EtOAc): R_f = 0.68; **Yield**: 1.60 g, 3.7 mmol (74%); **Optical rotation**: $[\alpha]_D^{25}$ = –58.6 (*c* 0.09, MeOH); **HRMS** (ESI$^+$): calculated for $C_{22}H_{32}N_3O_6^+$ 434.2286, found 434.2308; **^{1}H-NMR** (400 MHz, CD_3OD): δ (ppm) 7.71 and 7.60 (d, J = 7.0 Hz and 7.9 Hz, 1H),

7.39 and 7.36 (d, *J* = 7.4 Hz and 8.1 Hz, 1H), 7.16 and 7.12 (m, 1H), 7.13 and 7.11 (s, 1H), 7.12 and 7.05 (m and ddd, *J* = 8.0, 7.0, 1.1 Hz, 1H), 5.44 and 5.18 (dd, *J* = 11.5, 3.8 Hz and 9.9, 5.4 Hz, 1H), 4.43 and 3.80 (d and m, *J* = 4.8 Hz, 1H), 3.96 and 2.89 (m, 1H), 3.81 and 3.75 (s, 3H), 3.57 and 3.48 (dd, *J* = 15.0, 3.9 Hz, and 15.4, 5.5 Hz, 1H), 3.31 and 3.25 (m and dd, *J* = 14.8, 11.3 Hz, 1H) 2.99 and 2.92 (s, 3H), 1.45 (s, 9H), 1.18 and 0.28 (d, *J* = 6.3 Hz and 6.3 Hz, 3H); **^{13}C-NMR** (101 MHz, CD_3OD): *δ* (ppm) 175.2 and 173.7 (C), 172.8 and 172.1 (C), 157.7 and 157.6 (C), 138.1 and 138.0 (C), 128.5 and 128.1 (C), 125.0 and 124.2 (CH), 122.8 and 122.4 (CH), 120.4 and 119.8 (CH), 119.1 and 119.0 (CH), 112.6 and 112.3 (CH), 111.1 and 110.5 (C), 80.7 and 80.5 (C), 68.5 and 68.0 (CH), 62.1 and 60.5 (CH), 57.0 and 53.7 (CH), 53.0 and 52.7 (CH_3), 34.4 and 30.2 (CH_3), 28.6 (CH_3), 26.0 and 25.4 (CH_2), 19.9 and 18.4 (CH_3).

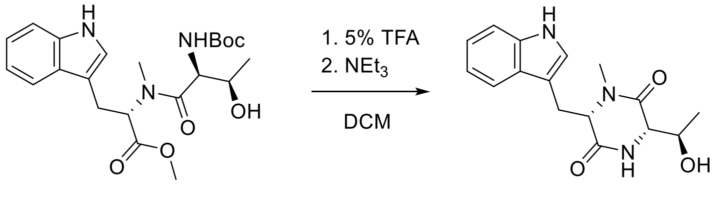

9 **10**

To a solution of **9** (85 mg, 0.20 mmol) in CH_2Cl_2 (1 mL) was added trifluoroacetic acid (0.05 mL). After stirring at room temperature for 30 min, the reaction mixture was concentrated under reduced pressure. Then another portion of CH_2Cl_2 (1 mL) was added to the residue, followed by triethylamine (40 mg, 0.40 mmol). After stirring at room temperature for 2 h, the reaction mixture was concentrated under reduced pressure again. The residue was then purified by column chromotography on silica gel (EtOAc/MeOH = 6:1) to give **10** as yellowish oil.

(3*S*,6*S*)-6-((1*H*-Indol-3-yl)methyl)-3-((*R*)-1-hydroxyethyl)-1-methylpiperazine-2,5-dione (10). TLC (EtOAc : MeOH = 6 : 1): R_f = 0.33; **Optical rotation**: $[\alpha]_D^{25}$ = –53.4 (*c* 0.31, MeOH); **Yield**: 58 mg, 0.19 mmol (96%); **HRMS** (ESI^+): calculated for $C_{16}H_{20}N_3O_3^+$ 302.1499, found 302.1496; **IR** (diamond ATR): $\tilde{\nu}$ / cm^{-1} = 3314 (m), 1673 (s), 1457 (m), 1346 (w), 1205 (m), 1140 (m), 801 (w), 747 (m); **^{1}H-NMR** (500 MHz, CD_3OD): *δ* (ppm) 7.64 (d, *J* = 7.8 Hz, 1H), 7.37 (d, *J* = 8.1 Hz, 1H), 7.11 (m, 2H), 7.04 (ddd, *J* = 7.9, 7.0, 1.0 Hz, 3H), 4.26 (dd, *J* = 7.3, 4.3 Hz, 1H), 3.66 (d, *J* = 4.4 Hz, 2H), 3.57 (dd, *J* = 14.8, 7.3 Hz, 1H), 3.49 (ddd, *J* = 14.9, 4.3, 0.9 Hz, 1H), 2.70 (s, 3H), 1.03 (d, *J* = 6.5 Hz, 3H); **^{13}C-NMR** (126 MHz, CD_3OD): *δ* (ppm) 170.7 (C), 167.4 (C), 138.0

(C), 128.8 (C), 125.1 (CH), 122.6 (CH), 120.1 (CH), 119.4 (CH), 112.4 (CH), 111.1 (C), 69.9 (CH), 64.9 (CH), 62.7 (CH), 34.6 (CH_3), 31.1 (CH_2), 20.0 (CH_3).

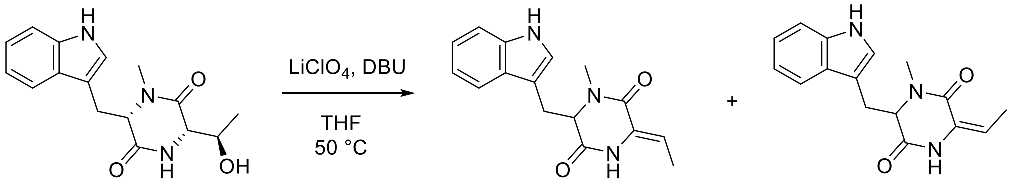

10 (*rac*)-*Z*-**4** (*rac*)-*E*-**4**

To a solution of **10** (0.45 g, 1.5 mmol) in THF (60 mL) was added $LiClO_4$ (3.20 g, 30.0 mmol) and DBU (0.57 g, 3.8 mmol) sequentially. After stirring at 50 °C for 3 days, the reaction mixture was diluted with H_2O (100 mL) and extracted with EtOAc (3 × 100 mL). The combined organic phases were dried with $MgSO_4$ and then concentrated under reduced pressure. The residue was purified by column chromatography on silica gel (EtOAc) to give (*Z*)-**4** and (*E*)-**4** as white solids.

(*Z*)-6-((1*H*-Indol-3-yl)methyl)-3-ethylidene-1-methylpiperazine-2,5-dione ((*rac*)-(*Z*)-4). TLC (EtOAc): R_f = 0.21; **Yield**: 0.24 g, 0.85 mmol (57%); **HRMS** (ESI^+): calculated for $C_{16}H_{18}N_3O_2^+$ 284.1394, found 284.1393; **IR** (diamond ATR): $\tilde{\nu}$ / cm^{-1} = 3254 (m), 2926 (m), 2854 (w), 1681 (s), 1630 (s), 1490 (w), 1400 (m), 1265 (w), 741 (m); **^{1}H-NMR** (700 MHz, CD_3OD): *δ* (ppm) 7.54 (d, *J* = 8.0 Hz, 1H), 7.29 (d, *J* = 8.1 Hz, 1H), 7.08 (ddd, *J* = 8.1, 7.0, 1.1 Hz, 1H), 6.98 (ddd, *J* = 8.0, 7.0, 1.0 Hz, 1H), 6.87 (s, 1H), 5.29 (q, *J* = 7.4 Hz, 1H), 4.32 (dd, *J* = 4.9, 2.9 Hz, 1H), 3.47 (ddd, *J* = 14.9, 3.0, 0.8 Hz, 1H), 3.10 (s, 3H), 0.91 (d, *J* = 7.5 Hz, 3H); **^{13}C-NMR** (176 MHz, CD_3OD): *δ* (ppm) 169.08 (C), 162.79 (C), 138.17 (C), 129.09 (C), 127.90 (C), 126.41 (CH), 122.41 (CH), 119.85 (CH), 119.45 (CH), 114.55 (CH), 111.95 (CH), 108.13 (C), 64.80 (CH), 33.08 (CH_3), 28.20 (CH_2), 10.17 (CH_3).

(*E*)-6-((1*H*-Indol-3-yl)methyl)-3-ethylidene-1-methylpiperazine-2,5-dione ((*rac*)-(*E*)-4). TLC (EtOAc): R_f = 0.18; **Yield**: 30 mg, 0.11 mmol (7%); **IR** (diamond ATR): $\tilde{\nu}$ / cm^{-1} = 3299 (m), 2924 (w), 1681 (s), 1631 (s), 1456 (m), 1400 (m), 1259 (w), 1104 (w), 764 (m); **^{1}H-NMR** (500 MHz, CD_3OD): *δ* (ppm) 7.49 (d, *J* = 7.9 Hz, 1H), 7.31 (d, *J* = 8.1 Hz, 1H), 7.08 (ddd, *J* = 8.2, 7.0, 1.2 Hz, 3H), 7.00 (ddd, *J* = 8.0, 7.0, 1.1 Hz, 1H), 6.94 (s, 1H), 4.59 (q, *J* = 7.5 Hz, 1H), 4.25 (dd, *J* = 4.7, 3.1 Hz, 1H), 3.42 (ddd, *J* = 14.8, 3.1, 0.7 Hz, 1H), 3.32 (m, 1H), 3.09 (s, 3H), 1.56 (d, *J* = 7.5 Hz, 3H); **^{13}C-NMR**

(126 MHz, CD_3OD): *δ* (ppm) 169.2 (C), 162.6 (C), 138.1 (C), 129.2 (C), 126.8 (C), 126.1 (CH), 122.4 (CH), 120.0 (CH), 119.4 (CH), 119.3 (CH), 111.9 (CH), 108.4 (C), 65.0 (CH), 32.9 (CH_3), 28.0 (CH_2), 13.0 (CH_3).

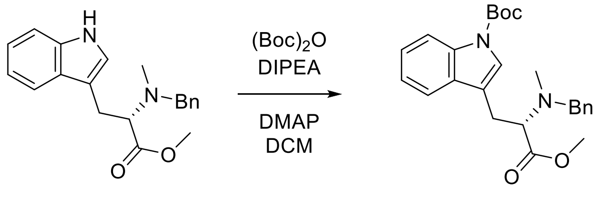

7 11

To a solution of **7** (0.46 g, 1.4 mmol) dissolved in CH_2Cl_2 (15 mL) was added $(Boc)_2O$ (0.47 g, 2.2 mmol), DMAP (35 mg, 0.28 mmol) and *N*,*N*-diisopropylethylamine (0.5 mL, 2.8 mmol). After stirring at room temperature for 2 h, the reaction mixture was diluted with EtOAc (200 mL) and washed with sat. aq. NH_4Cl solution (30 mL), sat. aq. $NaHCO_3$ solution (30 mL) and brine (30 mL). The organic layer was then dried with $MgSO_4$ and concentrated under reduced pressure. The residue was purified by column chromatography on silica gel (cyclohexane/EtOAc = 5:1) to give **11** as colorless oil.

***tert*-Butyl (*S*)-3-(2-(benzyl(methyl)amino)-3-methoxy-3-oxopropyl)-1*H*-indole-1-carboxylate (11). TLC** (cyclohexane/EtOAc = 5:1): R_f = 0.55; **Yield**: 0.59 g, 1.4 mmol (98%); **Optical rotation**: $[\alpha]_D^{25}$ = –34.6 (*c* 0.05, MeOH); **HRMS** (ESI^+): calculated for $C_{25}H_{31}N_2O_4^+$ 423.2278, found 423.2277; **IR** (diamond ATR): $\tilde{\nu}$ / cm^{-1} = 3009 (w), 1738 (s), 1452 (m), 1373 (s), 1256 (w), 1229 (m), 1217 (m); **^{1}H-NMR** (700 MHz, CD_3OD): *δ* (ppm) 8.11 (d, *J* = 8.1 Hz, 1H), 7.4 (m, 2H), 7.29 (ddd, *J* = 8.3, 7.2, 1.2 Hz, 1H), 7.23 (m, 5H), 7.18 (ddd, *J* = 8.0, 7.2, 1.0 Hz, 1H), 3.84 (d, *J* = 13.3 Hz, 1H), 3.72 (dd, *J* = 8.2, 6.9 Hz, 1H), 3.71 (s, 3H), 3.65 (d, *J* = 13.4 Hz, 1H), 3.21 (ddd, *J* = 14.6, 8.2, 1.0 Hz, 1H), 3.04 (ddd, *J* = 14.6, 7.0, 1.1 Hz, 1H), 2.38 (s, 3H), 1.69 (s, 9H); **^{13}C-NMR** (176 MHz, CD_3OD): *δ* (ppm) 173.7 (C), 151.0 (C), 140.2 (C), 136.8 (C), 131.8 (C), 130.0 (CH), 129.2 (CH), 128.1 (CH), 125.3 (CH), 124.8 (CH), 123.5 (CH), 119.9 (CH), 118.5 (CH), 116.1 (CH), 84.7 (C), 66.1 (CH), 60.1 (CH_2), 51.6 (CH_3), 38.2 (CH_3), 28.4 (CH_3), 26.2 (CH_2).

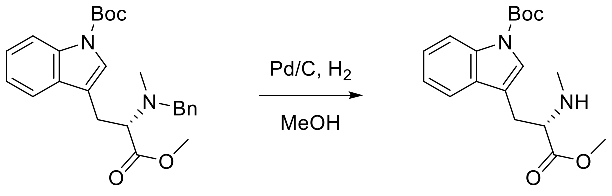

11 12

A solution of **11** (0.68 g, 1.61 mmol) in MeOH (10 mL) was added to palladium on charcoal (5 wt %, 68 mg, 10 % w/w) under argon atmosphere. The reaction mixture was stirred under the pressure of H_2 (4 bar) for 6 h. After filtration, the residue was concentrated under reduced pressure and purified by column chromotography on silica gel (EtOAc) to give **12** as a colorless oil.

***tert*-Butyl (*S*)-3-(3-methoxy-2-(methylamino)-3-oxopropyl)-1*H*-indole-1-carboxylate (12). TLC** (EtOAc): R_f = 0.30; **Yield**: 0.51 g, 1.53 mmol (95%); **Optical rotation**: $[\alpha]_D^{25}$ = 16.2 (*c* 0.18, MeOH); **HRMS** (ESI⁺): calculated for $C_{18}H_{25}N_2O_4^+$ 333.1809, found 333.1818; **IR** (diamond ATR): $\tilde{\nu}$ / cm^{-1} = 2976 (w), 1733 (s), 1453 (m), 1370 (s), 1228 (m), 1158 (m), 1083 (m), 793 (w), 746 (w); **^{1}H-NMR** (500 MHz, CD_3OD): *δ* (ppm) δ 8.12 (d, *J* = 8.3 Hz, 1H), 7.55 (ddd, *J* = 7.7, 1.3, 0.7 Hz, 1H), 7.47 (s, 1H), 7.31 (ddd, *J* = 8.4, 7.3, 1.3 Hz, 1H), 7.25 (ddd, *J* = 8.1, 7.3, 1.1 Hz, 1H), 3.62 (s, 3H), 3.56 (t, *J* = 6.8 Hz, 1H), 3.09 (dd, *J* = 6.8, 1.0 Hz, 2H), 2.36 (s, 3H), 1.69 (s, 9H); **^{13}C-NMR** (126 MHz, CD_3OD): *δ* (ppm) 175.6 (C), 150.9 (C), 136.8 (C), 131.6 (C), 125.5 (CH), 125.1 (CH), 123.6 (CH), 119.9 (CH), 117.2 (CH), 116.1 (CH), 84.8 (C), 64.2 (CH), 52.2 (CH_3), 34.5 (CH_3), 29.2 (CH_2), 28.4 (CH_3).

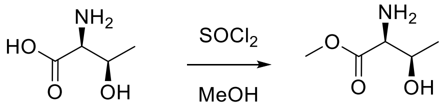

S1 S3

$SOCl_2$ (2.51 mL, 34.5 mmol) was added to MeOH (80 mL) dropwise at 0 °C. The mixture was then stirred at 0 °C for 30 min, followed by the addition of L-threonine (**S1**) (3.00 g, 25.2 mmol). After the reaction mixture was refluxed for 4 h, the solvent was evaporated to give **S3** as white solid.

Methyl L-threoninate (S3). Yield: 3.33 g, 25.0 mmol (99%); **Optical rotation**: $[\alpha]_D^{25}$ = 5.7 (*c* 0.14, MeOH); **^{1}H-NMR** (500 MHz, CD_3OD): *δ* (ppm) 4.11 (qd, *J* = 6.5, 4.0 Hz, 1H), 3.81 (s, 3H), 3.39 (d, *J* = 4.0 Hz, 1H), 1.30 (d, *J* = 6.5 Hz, 3H); **^{13}C-NMR** (126 MHz, CD_3OD): *δ* (ppm) 175.5 (C), 69.5 (CH), 61.1 (CH), 52.5 (CH_3), 20.1 (CH_3).

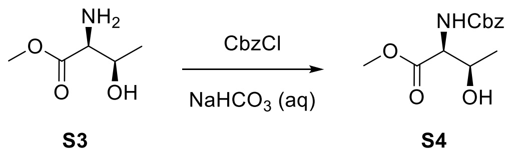

Compound **S3** (3.33 g, 25.0 mmol) was added to a solution of $NaHCO_3$ (4.62 g, 55.0 mmol) in H_2O (250 mL), followed by Cbz-Cl (3.9 mL, 27.5 mmol). After stirring at room temperature for 5 h, the reaction mixture was extracted with EtOAc (3 × 150 mL). The organic phases were combined, dried with $MgSO_4$ and then concentrated under reduced pressure. The residue was purified by column chromatography on silica gel (cyclohexane/EtOAc = 1:1) to give **S4** as white solid.

Methyl ((benzyloxy)carbonyl)-L-threoninate (S4). Yield: 6.54 g, 24.5 mmol (98%); **Optical rotation**: $[\alpha]_D^{25}$ = –11.0 (*c* 0.32, MeOH); **^{1}H-NMR** (500 MHz, CD_3OD): *δ* (ppm) 7.34 (m, 5H), 5.15 (d, *J* = 1.5 Hz, 2H), 4.29 (qd, *J* = 6.4, 3.2 Hz, 1H), 4.25 (d, *J* = 3.1 Hz, 1H), 3.77 (s, 3H), 1.22 (d, *J* = 6.4 Hz, 3H); **^{13}C-NMR** (126 MHz, CD_3OD): *δ* (ppm) 172.9 (C), 158.9 (C), 138.1 (C), 129.5 (CH), 129.0 (CH), 129.0 (CH), 128.8 (CH), 128.8 (CH), 68.4 (CH), 67.8 (C_2), 61.2 (CH), 52.7 (CH_3), 20.2 (CH_3).

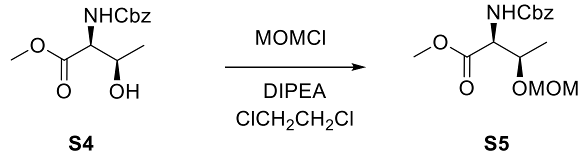

To a solution of **S4** (0.27 g, 1.0 mmol) in 1,2-dichloroethane (10 mL) was added *N*,*N*-diisopropylethylamine (0.26 g, 2.0 mmol). The mixture was then cooled to 0 °C and MOM-Cl (0.12 g, 1.5 mmol) was added. After refluxing for 5 h, the reaction was quenched with sat. aq. NH_4Cl solution (20 mL) and then extracted with EtOAc (3 × 20 mL). The organic layers were combined, dried with $MgSO_4$ and then concentrated in vacuo. The residue was purified by column chromatography on silica gel (cyclohexane/EtOAc = 1:1) to give **S5** as colorless oil.

Methyl *N*-((benzyloxy)carbonyl)-*O*-(methoxymethyl)-L-threoninate (S5). Yield: 0.30 g, 0.96 mmol (96%); **Optical rotation**: $[\alpha]_D^{25}$ = –24.1 (*c* 0.22, MeOH); **^{1}H-NMR** (500 MHz, CD_3OD): *δ* (ppm) 7.37 (m, 5H), 5.15 (s, 2H), 4.66 (d, *J* = 7.0 Hz, 1H), 4.55 (d, *J* = 7.0 Hz, 1H), 4.34 (d, *J* = 2.9 Hz, 1H), 4.31 (qd, *J* = 6.3, 3.0 Hz, 1H), 3.77 (s, 3H),

3.30 (s, 3H), 1.24 (d, *J* = 6.3 Hz, 3H); **^{13}C-NMR** (126 MHz, CD_3OD): *δ* (ppm) 172.7 (C), 159.1 (C), 138.2 (C), 129.5 (CH), 129.0 (CH), 129.0 (CH), 128.8 (CH), 128.8 (CH), 96.0 (CH_2), 73.9 (CH), 67.8 (CH_2), 60.4 (CH), 55.9 (CH_3), 52.8 (CH_3), 17.3 (CH_3).

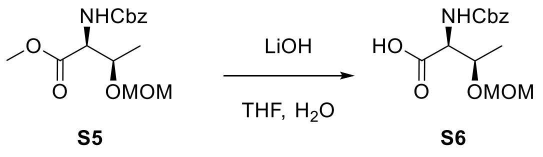

To a solution of **S5** (0.30 g, 0.96 mmol) in THF/H_2O (4:1, 5 mL) was added LiOH (49 mg, 2.03 mmol). The reaction mixture was stirred at room temperature for 3 h and then diluted with EtOAc (30 mL). The solution was treated with aq. HCl solution (1 N) dropwise at 0 °C until pH = 2 of the aqueous phase was reached. Then the aqueous phase was extracted with EtOAc (2 × 20 mL). The organic layers were combined, dried with $MgSO_4$ and concentrated in vacuo to give **S6** as a white solid.

***N*-((Benzyloxy)carbonyl)-*O*-(methoxymethyl)-L-threonine (S6). Yield**: 0.27 g, 0.91 mmol (95%); **Optical rotation**: $[\alpha]_D^{25}$ = 2.4 (*c* 0.13, MeOH); **^{1}H-NMR** (500 MHz, CD_3OD): *δ* (ppm) 7.35 (m, 5H), 5.12 (d, *J* = 3.4 Hz, 1H), 4.66 (d, *J* = 6.9 Hz, 1H), 4.56 (d, *J* = 6.9 Hz, 1H), 4.33 (qd, *J* = 6.4, 2.8 Hz, 1H), 4.26 (d, *J* = 2.7 Hz, 1H), 3.31 (s, 3H), 1.22 (d, *J* = 6.3 Hz, 3H); **^{13}C-NMR** (126 MHz, CD_3OD): *δ* (ppm) 173.9 (C), 159.1 (C), 138.2 (C), 129.5 (CH), 129.0 (CH), 129.0 (CH), 128.8 (CH), 128.8 (CH), 95.8 (CH_2), 73.7 (CH), 67.8 (CH_2), 60.2 (CH), 55.9 (CH_3), 17.3 (CH_3).

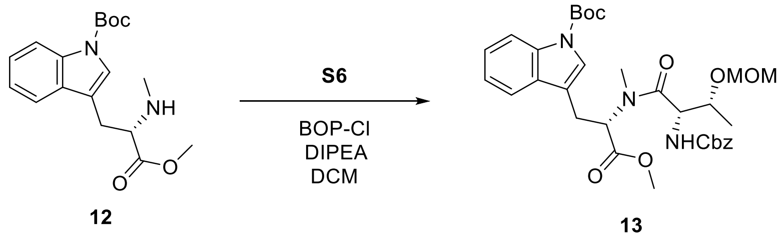

Compound **S6** (0.43 g, 1.45 mmol) was added to a solution of **12** (0.48 g, 1.45 mmol) and *N*,*N*-diisopropylethylamine (0.61 mL, 3.47 mmol) in CH_2Cl_2 (20 mL). Then BOP-Cl (0.41 g, 1.60 mmol) was added after the mixture was cooled to 0 °C. The reaction solution was stirred at room temperature for 8 h and then diluted with EtOAc (200 mL). After washing with sat. aq. NH_4Cl solution (30 mL), sat. aq. $NaHCO_3$ solution (30 mL)

and brine (30 mL), the organic phase was dried with $MgSO_4$ and concentrated in vacuo. The residue was then purified by column chromotography on silica gel (cyclohexane/EtOAc = 1:1) to give **13** as a pale yellow oil.

***tert*-Butyl 3-((*S*)-2-((2*S*,3*R*)-2-(((benzyloxy)carbonyl)amino)-3-(methoxymethoxy)-*N*-methylbutanamido)-3-methoxy-3-oxopropyl)-1*H*-indole-1-carboxylate (13).** **TLC** (cyclohexane : EtOAc = 1 : 1): R_f = 0.42; **Yield**: 0.64 g, 1.05 mmol (73%); **HRMS** (ESI$^+$): calculated for $C_{32}H_{42}N_3O_9^+$ 612.2916, found 612.2916; **IR** (diamond ATR): $\tilde{\nu}$ / cm^{-1} = 2978 (w), 1721 (s), 1648 (m), 1453 (m), 1370 (s), 1254 (s), 1217 (s), 1154 (s), 1030 (s), 747 (m), 698 (w); **^{1}H-NMR** (500 MHz, CD_3OD): δ (ppm) 8.14 and 8.10 (d, J = 8.3 Hz and 8.2 Hz, 1H), 7.76 and 7.63 (d, J = 6.7 Hz and 7.7 Hz, 1H), 7.56 and 7.48 (s, 1H), 7.34 (m, 7H), 5.40 and 5.27 (dd, J = 10.0, 5.5 Hz and 11.2, 3.9 Hz, 1H), 5.10 and 5.01 (d, J = 12.3 Hz and 12.4 Hz, 2H), 4.60 and 4.25 (s, 2H), 4.58 and 4.16 (d, J = 5.7 Hz and 6.2 Hz, 1H), 3.96 and 3.33 (m, 1H), 3.75 and 3,71 (s, 3H), 3.52 and 3.31(dd and m, J = 14.6, 3.3 Hz, 1H), 3.43 and 3.26 (dd, J = 15.5, 6.1 Hz and 15.4, 10.0 Hz, 1H), 3.30 and 3.11 (s, 3H), 3.08 and 2.93(s, 3H), 1.68 and 1.66 (s, 9H), 1.22 and 0.34 (d, J = 6.3 Hz and 6.2 Hz, 3H); **^{13}C-NMR** (126 MHz, CD_3OD): δ (ppm) 173.4 and 173.0 (C), 172.2 and 171.8 (C), 158.4 and 158.2 (C), 151.0 and 150.9 (C), 138.2 and 138.1 (C), 137.0 and 136.8 (C), 131.5 and 131.1 (C), 129.5 and 129.4 (CH), 129.0 and 128.9 (CH), 129.0 and 128.9 (CH), 125.8 and 125.8 (CH), 125.4 and 125.1 (CH), 124.1 and 123.7 (CH), 120.0 and 119.9 (CH), 117.3 and 116.7 (C), 116.5 and 116.1 (CH), 96.9 and 96.4 (CH_2), 84.9 and 84.8 (C), 74.8 and 74.2 (CH), 67.8 and 67.7 (CH_2), 61.1 and 59.0 (CH), 56.9 and 56.2 (CH), 55.9 and 55.6 (CH_3), 53.2 and 52.8 (CH_3), 33.9 and 30.0 (CH_3), 28.4 and 28.4 (CH_3), 25.4 and 25.1 (CH_2), 18.0 and 17.4 (CH_3).

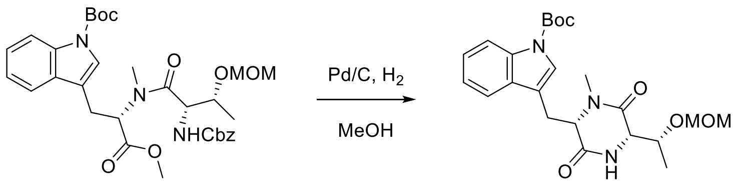

13 14

A solution of **13** (100 mg, 0.164 mmol) in MeOH (5 mL) was added to palladium on charcoal (5 wt %, 10 mg, 10% w/w) under argon atmosphere. The reaction mixture was stirred under the pressure of H_2 (4 bar) for 3 h. After filtration, the residue was

concentrated under reduced pressure and purified by column chromotography on silica gel (EtOAc/MeOH = 12:1) to give **14** as colorless oil.

***tert*-Butyl 3-(((2*S*,5*S*)-5-((*R*)-1-(methoxymethoxy)ethyl)-1-methyl-3,6-dioxopiperazin-2-yl)methyl)-1*H*-indole-1-carboxylate (14).** **TLC** (EtOAc : MeOH = 8 :1): R_f = 0.46; **Yield**: 70 mg, 0.157 mmol (96%); **Optical rotation**: $[\alpha]_D^{25}$ = –19.4 (*c* 0.17, MeOH); **HRMS** (ESI$^+$): calculated for $C_{23}H_{32}N_3O_6^+$ 446.2286, found 446.2285; **IR** (diamond ATR): $\tilde{\nu}$ / cm^{-1} = 2976 (w), 1733 (s), 1684 (s), 1665 (s), 1454 (m), 1370 (s), 1256 (m), 1158 (s), 1086 (m), 1030 (m), 765 (w), 749 (w); **^{1}H-NMR** (700 MHz, CD_3OD): *δ* (ppm) 8.15 (d, *J* = 8.3 Hz, 1H), 7.65 (d, *J* = 7.8 Hz, 1H), 7.50 (s, 1H), 7.34 (ddd, *J* = 8.4, 7.2, 1.2 Hz, 1H), 7.27 (ddd, *J* = 8.0, 7.1, 1.0 Hz, 1H), 4.42 (d, *J* = 6.8 Hz, 1H), 4.35 (t, *J* = 5.4 Hz, 1H), 4.18 (d, *J* = 6.8 Hz, 1H), 3.74 (d, *J* = 5.3 Hz, 1H), 3.48 (dd, *J* = 14.9, 5.7 Hz, 1H), 3.43 (dd, *J* = 14.9, 5.1 Hz, 1H), 3.25 (s, 3H), 2.98 (s, 3H), 1.69 (s, 9H), 1.04 (d, *J* = 6.4 Hz, 3H); **^{13}C-NMR** (176 MHz, CD_3OD): *δ* (ppm) 169.6 (C), 166.6 (C), 150.9 (C), 136.8 (C), 131.8 (C), 126.0 (CH), 125.7 (CH), 123.9 (CH), 120.2 (CH), 116.8 (C), 116.4 (CH), 96.4 (CH_2), 85.0 (C), 76.4 (CH), 64.0 (CH), 61.6 (CH), 55.9 (CH_3), 34.1 (CH_3), 29.2 (CH_2), 28.4 (CH_3), 17.1 (CH_3).

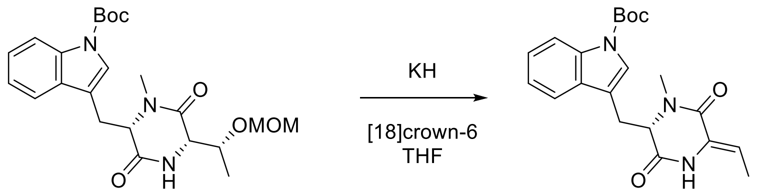

14 15

Potassium hydride (30 wt % in mineral oil, 20 mg, 0.15 mmol) was added to THF (1 mL), followed by [18]crown-6 (40 mg, 0.15 mmol). The mixture was stirred at room temperature for 30 min and then a solution of **14** (45 mg, 0.10 mmol) in THF (1 mL) was added at 0°C. After stirring at 0°C for 3 h, the reaction mixture was quenched with sat. aq. NH_4Cl solution (10 mL) and extracted with EtOAc (3 × 10 mL). The organic layers were combined, dried with $MgSO_4$ and concentrated under reduced pressure. The residue was purified by column chromatography on silica gel (EtOAc) to give **15** as colorless oil.

***tert*-Butyl (*S*,*Z*)-3-((5-ethylidene-1-methyl-3,6-dioxopiperazin-2-yl)methyl)-1*H*-indole-1-carboxylate (15). TLC** (EtOAc): R_f = 0.34; **Yield**: 27 mg, 0.07 mmol (70%); **Optical rotation**: $[\alpha]_D^{25}$ = –119.3 (*c* 0.14, MeOH); **HRMS** (ESI+): calculated for $C_{21}H_{25}N_3O_4Na^+$ 406.1737, found 406.1734; **IR** (diamond ATR): $\tilde{\nu}$ / cm^{-1} = 2971 (w), 1736 (s), 1684 (w), 1454 (w), 1371 (s), 1257 (w), 1229 (m), 1216 (m), 1086 (s), 763 (w); **^{1}H-NMR** (700 MHz, CD_3OD): δ (ppm) 8.06 (d, J = 8.4 Hz, 1H), 7.55 (d, J = 8.0 Hz, 1H), 7.29 (m, 2H), 7.21 (ddd, J = 8.1, 7.2, 1.0 Hz, 1H), 5.38 (q, J = 7.5 Hz, 1H), 4.38 (dd, J = 4.8, 3.1 Hz, 1H), 3.37 (m, 2H), 3.14 (s, 3H), 1.68 (s, 9H), 1.04 (d, J = 7.5 Hz, 3H); **^{13}C-NMR** (176 MHz, CD_3OD): δ (ppm) 168.4 (C), 162.4 (C), 150.8 (C), 136.8 (C), 131.9 (C), 128.3 (C), 127.1 (CH), 125.5 (CH), 123.6 (CH), 120.1 (CH), 115.8 (CH), 114.8 (CH), 114.7 (C), 85.0 (C), 64.2 (CH), 33.1 (CH_3), 28.3 (CH_3), 27.6 (CH_2), 10.5 (CH_3).

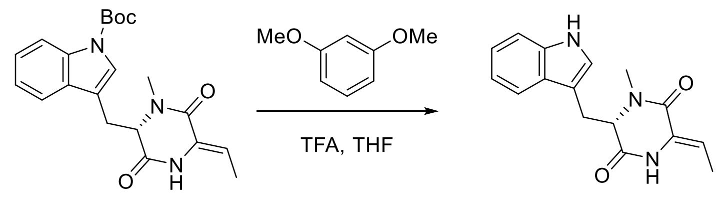

15 **4**

Compound **15** (18 mg, 0.047 mmol), 1,3-dimethoxybenzene (0.1 mL) and TFA (2 mL) were sequentially added into a reaction flask. The reaction mixture was then stirred at room temperature for 30 min. After removal of TFA under reduced pressure, EtOAc (10 mL) and sat. aq. $NaHCO_3$ solution (10 mL) were added. The mixture was stirred overnight and then extracted with EtOAc (2 × 10 mL). The organic layers were combined, dried with $MgSO_4$ and concentrated under reduced pressure. The residue was then purified by column chromatography on silica gel (EtOAc) to give **4** (90% ee by chiral HPLC).

(*S*,*Z*)-6-((1*H*-Indol-3-yl)methyl)-3-ethylidene-1-methylpiperazine-2,5-dione (4). Yield: 12.6 mg, 0.045 mmol (95%); **Optical rotation**: $[\alpha]_D^{25}$ = –15.5 (*c* 0.10, MeOH). Other spectroscopic data were the same as for (*rac*)-(*Z*)-**4** reported above.

Chiral HPLC

Analytical-scale HPLC seperations were carried out using a PLATINblue series HPLC system (Knauer, Berlin, Germany), equipped with PAD-1 photodiode array detector (190–1000 nm) and a DAICEL Chiralpak IA column (5.0 µm; 4.6 mm × 250 mm). The UV-Vis absorption was monitored at 190–600 nm. HPLC parameters: 1) temperature: 25 °C, 2) injection volume: 2 µL, 3) eluent: acetonitrile, 4) flow rate: 1.0 mL min^{-1}, and 5) pressure: 52 bar.

Single crystal X-ray diffraction study

Single crystals of (*rac*)-**4** were grown from a concentrated solution in methanol upon standing at −20 °C. The data collection of a suitable clear colourless plate-like crystal was perfomed on a Bruker D8 Venture diffractometer using Cu-K_α radiation (λ = 1.54178 Å). The diffractometer was equipped with a low-temperature device (Cryostream 800er series, Oxford Cryosystems, 100(2) K). Intensities were measured by fine-slicing ω- and φ-scans and corrected for background, polarisation and Lorentz effects. An empirical absorption correction was applied for the data set following Blessing's method [2]. The structure was solved by intrinsic phasing methods [3] and refined anisotropically by the least-squares procedure implemented in the XL program system [4]. The carbon-bonded hydrogen atoms were included isotropically using the riding model on the bound carbon atoms, the positions of hydrogen atoms at hetero atoms (O, N) were refined freely. CCDC 2182500 contains the supplementary crystallographic data for this paper, which can be obtained free of charge from The Cambridge Crystallographic Data Centre via www.ccdc.cam.ac.uk/data_request/cif.

Table S1. Crystal data and structure refinement for (*rac*)-**4**.

crystal habitus	clear colourless plate
device type	Bruker D8 Venture
empirical formula	$C_{17}H_{21}N_3O_3$
moiety formula	$C_{16}H_{17}N_3O_2$, CH_4O
formula weight	315.37 Da
temperature / K	100(2)
crystal system	monoclinic
space group	$P2_1/c$ (no. 14)
a / Å	14.4700(4)
b / Å	12.2361(4)
c / Å	9.0324(3)
α / °	90
β / °	95.1840(10)
γ / °	90
volume / $Å^3$	1592.70(9)
Z	4
ρ_{calc} / g cm^{-3}	1.315
μ / mm^{-1}	0.747
F(000)	672
crystal size/mm^3	0.22 × 0.16 × 0.04
absorption correction	empirical
*T*min; *T*max	0.4716; 0.7536
radiation	CuK_α (λ = 1.54178)
2Θ range for data collection / °	6.132 to 135.464°
completeness to Θ	0.993
index ranges	–17 ≤ h ≤ 17, –14 ≤ k ≤ 14, –10 ≤ l ≤ 10
reflections collected	30098
independent reflections	2873 [R_{int} = 0.0516, R_{sigma} = 0.0328]
data / restraints / parameters	2873 / 0 / 220
goodness-of-fit on F^2	1.052
final R indexes [I>=2σ (I)]	R_1 = 0.0441, wR_2 = 0.1166
final R indexes [all data]	R_1 = 0.0454, wR_2 = 0.1182
Largest diff. peak/hole / e $Å^{-3}$	0.33 / –0.37

Determination of the minimal inhibitory concentration (MIC)

The minimal inhibitory concentration (MIC) was determined in cation-adjusted Mueller-Hinton medium (MH II) that contains casein, beef extract and starch by using a two-fold serial dilution method according to the standards and guidelines of the Clinical and Laboratory Standards Institute (CLSI) [5]. Due to solubility issues of some of the compounds at high concentrations, a predilution of all compounds was made in DMSO. The test compounds were dissolved in DMSO at 20 mg/mL and a two-fold serial dilution was prepared in round-bottom polystyrene microtiter plates (Sarstedt, Germany) in DMSO; 1.28 µL of the DMSO solution were transferred to 48,7 µL MHII and seeded with 50 mL of a bacterial suspension in MH II to yield a final inoculum of 5×10^5 colony-forming units per mL. After overnight incubation at 37 °C without shaking in an incubator, the MIC was determined as the lowest compound concentration preventing visible bacterial growth. The strain panel included representative species of nosocomial pathogens, which are known as “ESKAPE” bacteria [6]. Specifically, the following strains were used: *Enterococcus faecium* BM 4147-1 [7], *Staphylococcus aureus* ATCC 29213, *Klebsiella pneumoniae* ATCC 12657, *Acinetobacter baumannii* 09987, *Pseudomonas aeruginosa* ATCC 27853 and *Enterobacter aerogenes* ATCC 13048. *Bacillus subtilis* 168 (*trpC2*) [8], *Escherichia coli* HN817 and *E. coli* HN818 [9,10] were used as further reference strains. The ATCC strains were provided by the American Type Culture Collection. *A. baumannii* 09987 was obtained from the University of Bonn, Germany.

Growth curves (MIC)

For continuous recording growth curves, samples were prepared as above with the exception that flat-bottom polystyrene plates (Sarstedt, Germany) were used and that the plates were incubated at 37 °C in a Tecan SPARK microplate reader overnight with intermittent shaking for 30s every 10 min. The optical density at 600 nm was recorded directly after each shaking interval. The medium background of the sterile control was subtracted from the OD_{600} values of the bacterial cultures. Means and standard deviations were calculated using GraphPad Prism.

Table S2. Determination of the Minimal Inhibitory Concentration (MIC).[a]

**Bacterial Strain**	**MIC [µg/mL]**				
	**1**	**4**	(*rac*)-(*Z*)-**4**	(*rac*)-(*E*)-**4**	HTM_{222}
Bacillus subtilis 168	>256[b]	>256	>256	>256	> 256
Staphylococcus aureus ATCC 29213	>256	n.d.	n.d.	n.d.	n.d.
Enterococcus faecium BM 4147-1	>256	n.d.	n.d.	n.d.	n.d.
Escherichia coli HN817 (wildtype)	>256	>256	>256	>256	> 256
E. coli HN818 (Δ*acrAB*)	>256	n.d.	n.d.	n.d.	n.d.
E. coli HN818 (Δ*acrAB*), PMBN 10 µg/ml	128	>256	>256	>256	> 256
Klebsiella pneumoniae ATCC 12657	>256	n.d.	n.d.	n.d.	n.d.
Enterobacter aerogenes ATCC 13048	>256	n.d.	n.d.	n.d.	n.d.
Pseudomonas aeruginosa ATCC 27853	>256	n.d.	n.d.	n.d.	n.d.
Acinetobacter baumannii 09987	>256[b]	>256	>256	>256	> 256

[a] Tested bacterial strains reflect the ESKAPE species, i.e., multi-resistant species designated by the World Health Organisation as the target for future antibiotic research efforts with critical and high priority [6].

[b] Although no clear MIC could be determined in these cases due to residual growth of the cultures, growth retardation occurred when *B. subtilis* 168 and *A. baumannii* 09987 were exposed to **1** (see Figure 3 of main text). In contrast, when growth curves of these two strains were recorded in the presence of up to 256 µg/ml of **4**, (*rac*)-(*Z*)-**4**, (*rac*)-(*E*)-**4** or HTM_{222}, growth was not affected (data not shown). N.d., not determined.

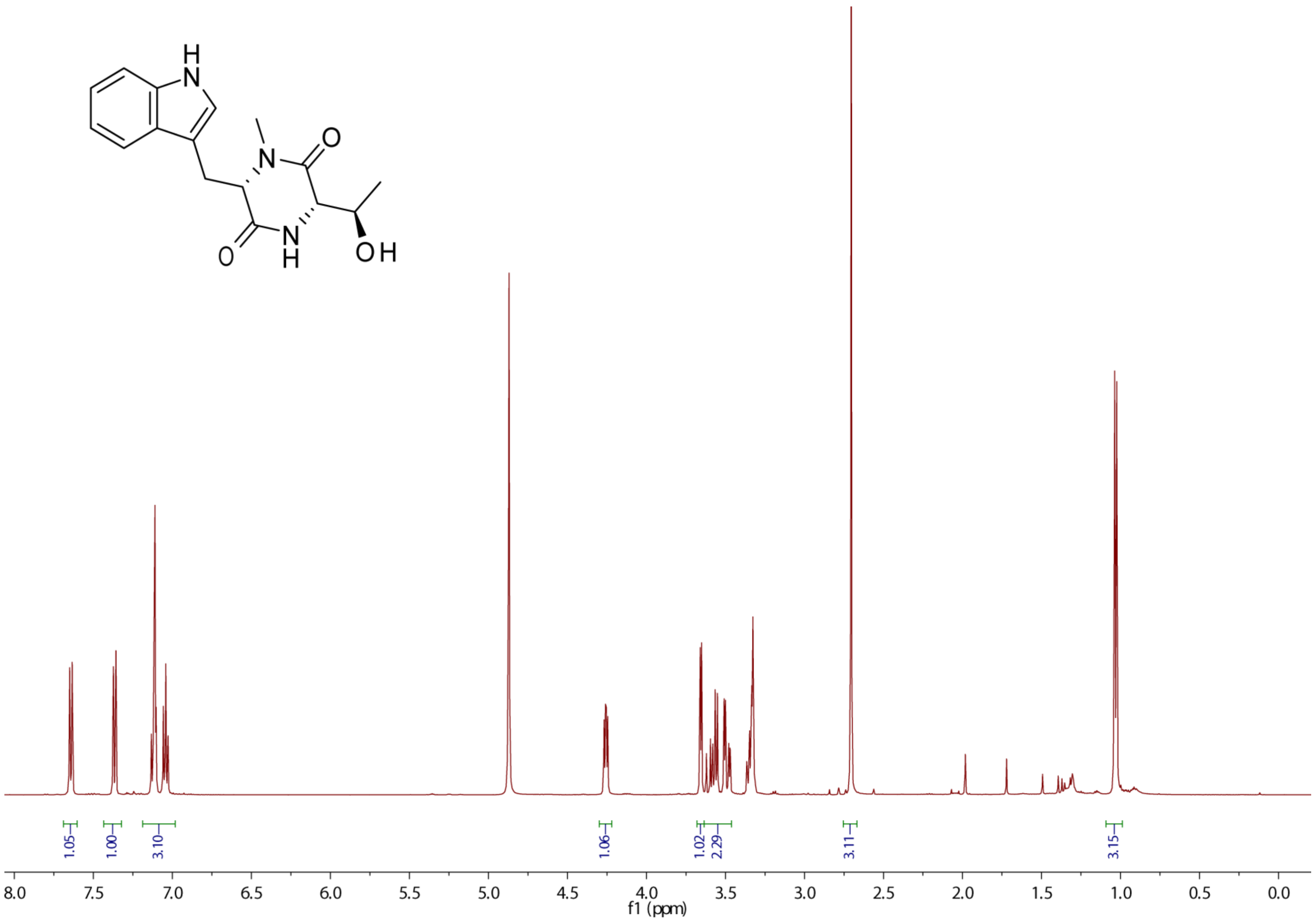

Figure S1. ^{1}H NMR spectrum (500 MHz, CD_3OD) of **10**.

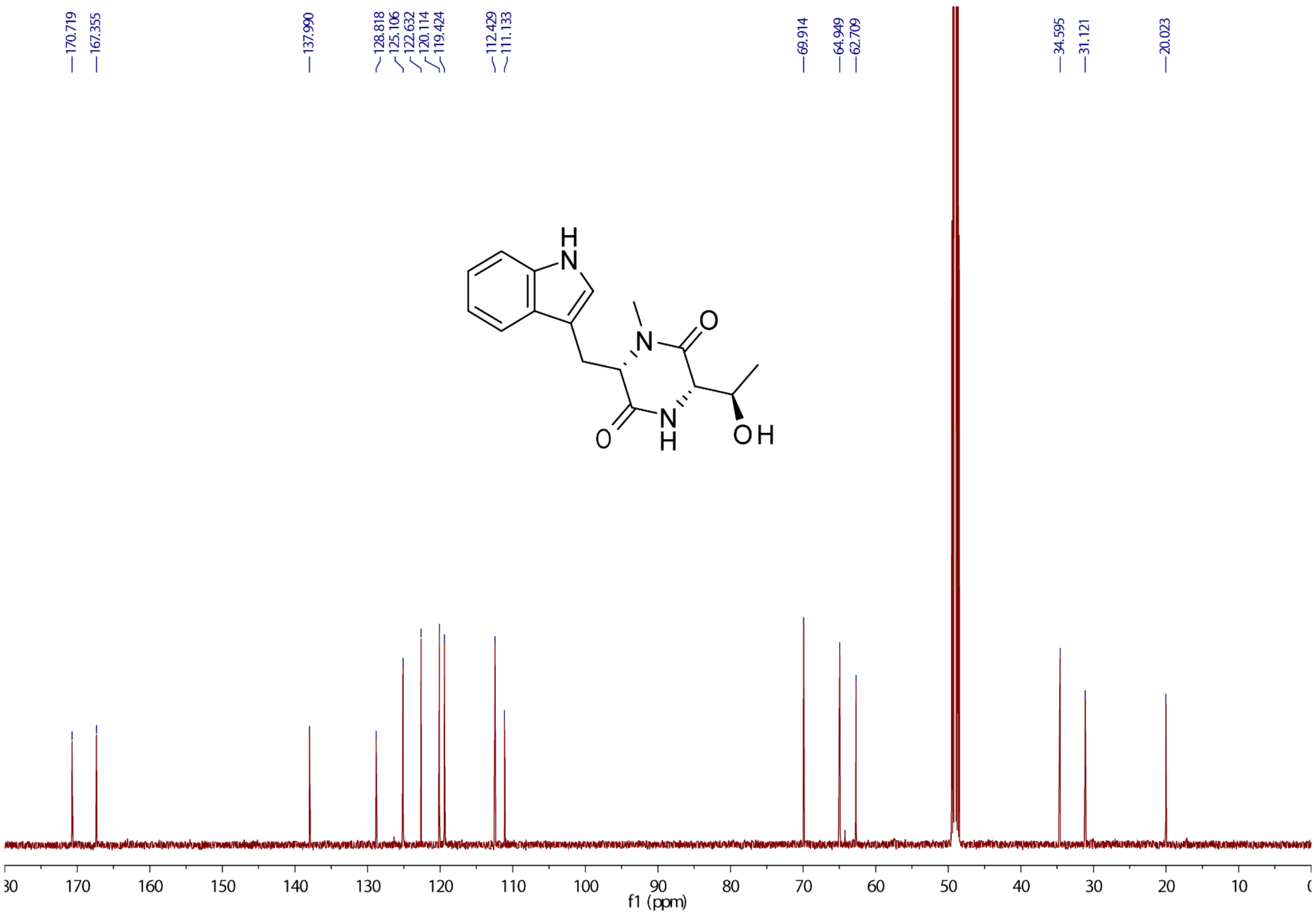

Figure S2. ^{13}C NMR spectrum (126 MHz, CD_3OD) of **10**.

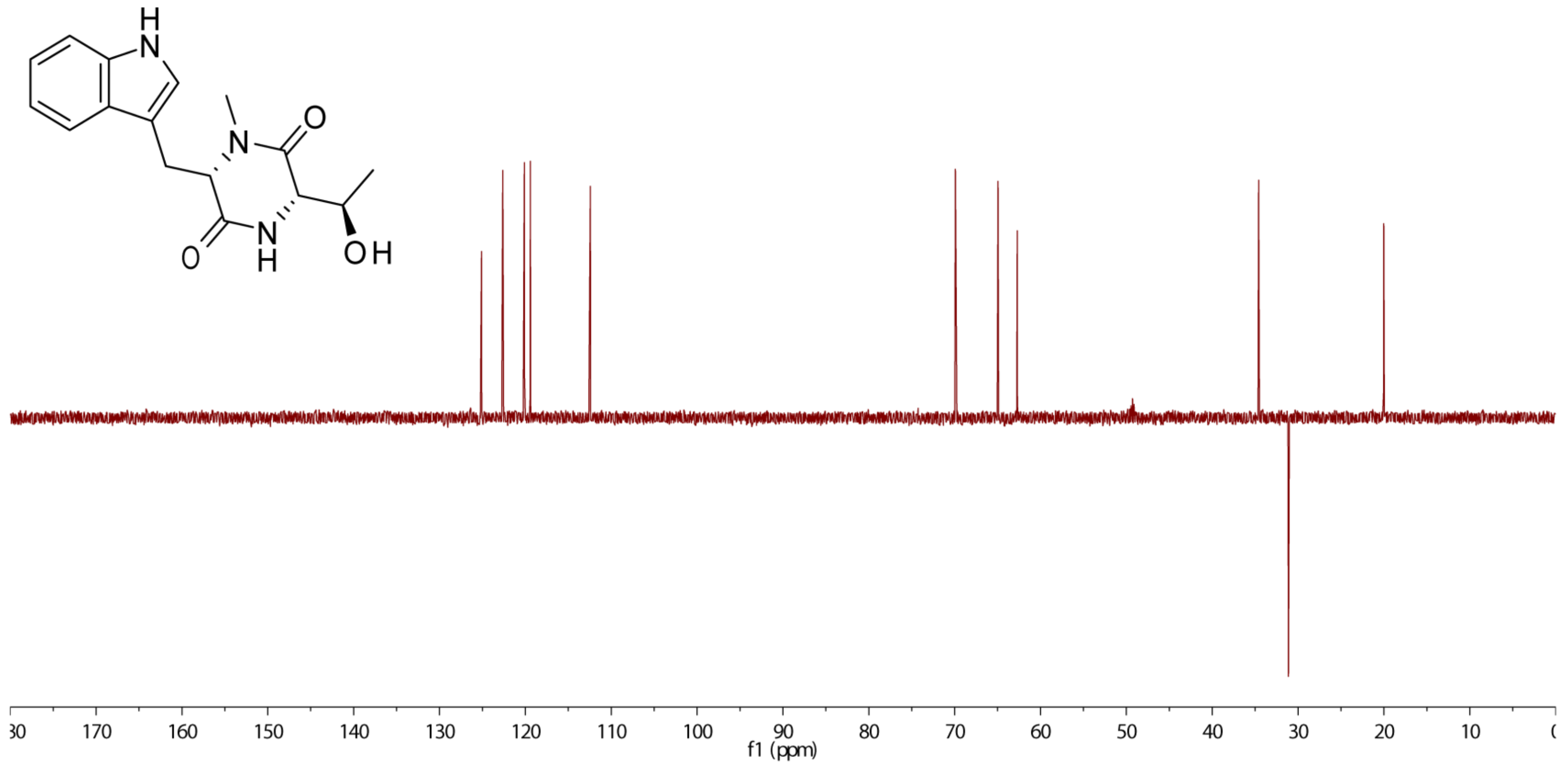

Figure S3. ^{13}C-DEPT-135 NMR spectrum (126 MHz, CD_3OD) of **10**.

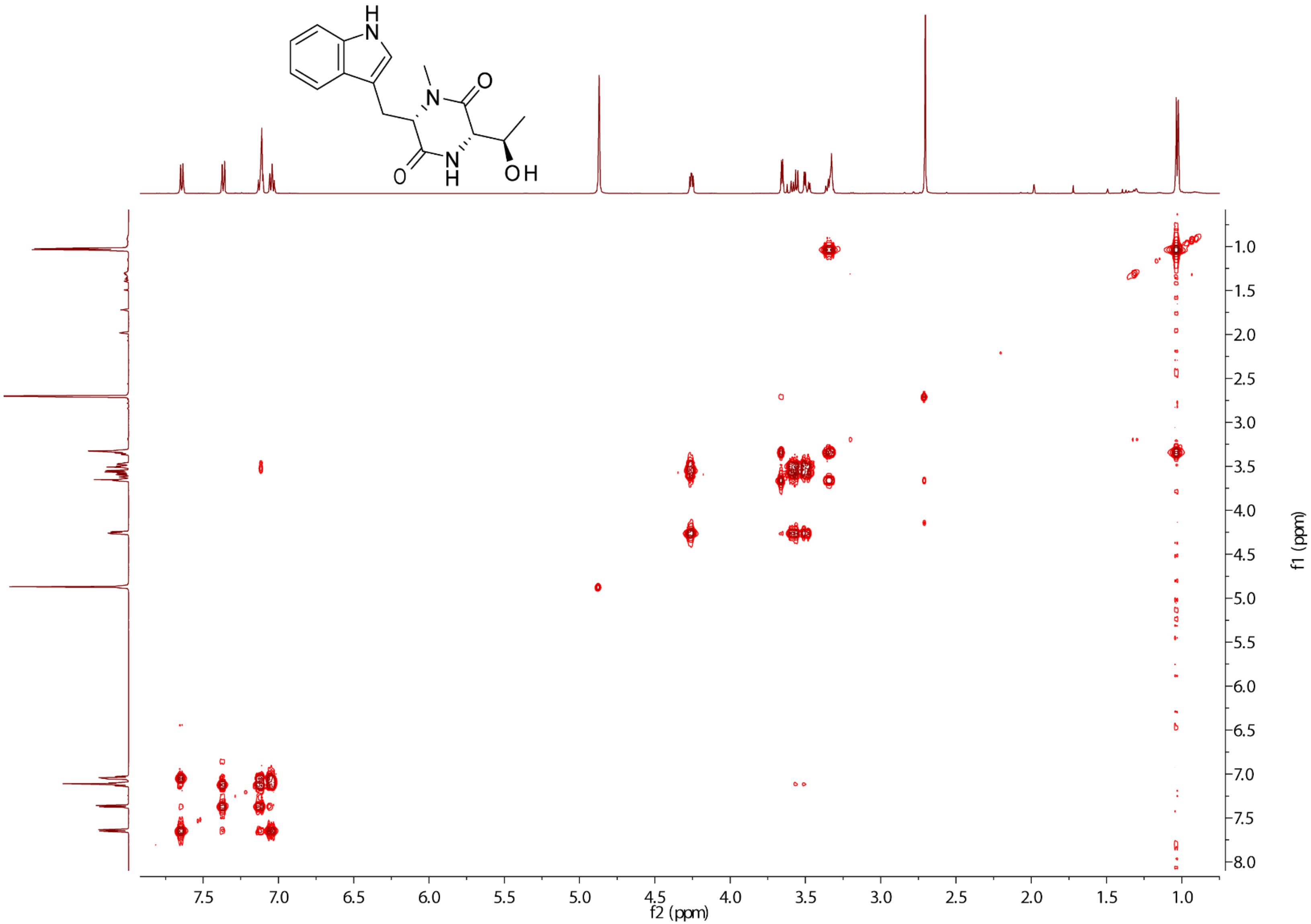

Figure S4. ^{1}H-^{1}H COSY spectrum (500 MHz, CD_3OD) of **10**.

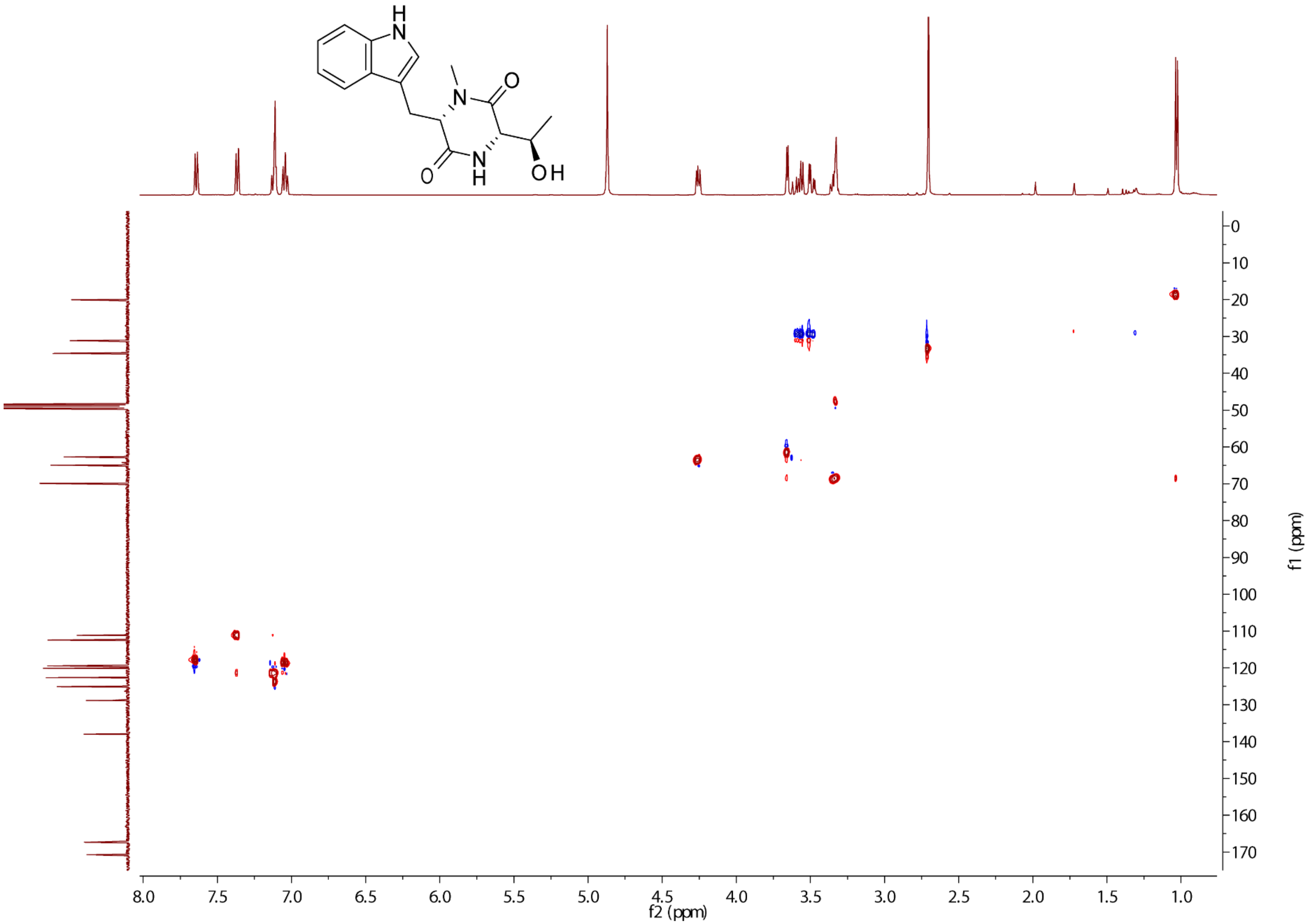

Figure S5. HSQC spectrum (CD_3OD) of **10**.

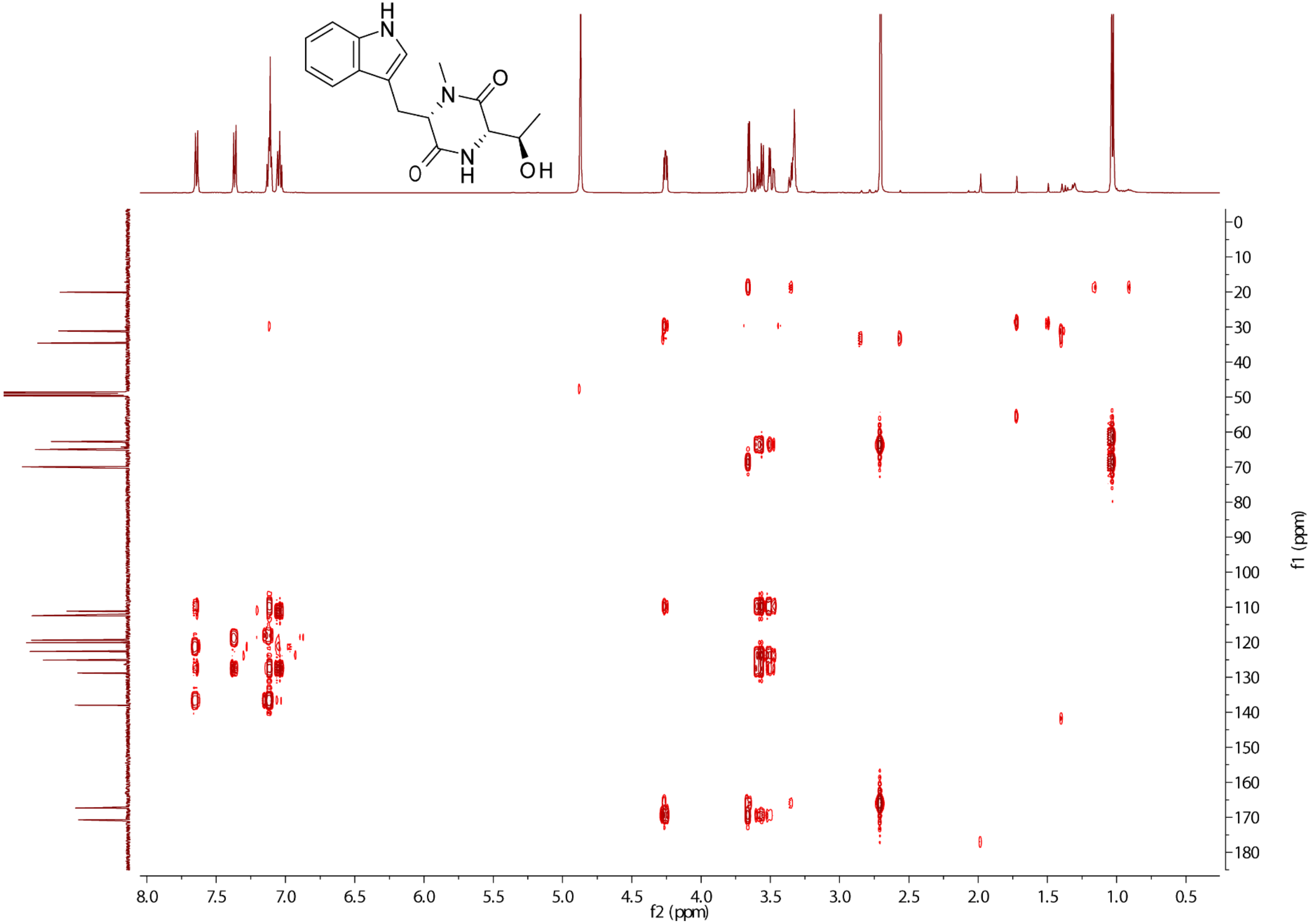

Figure S6. HMBC spectrum (CD_3OD) of **10**.

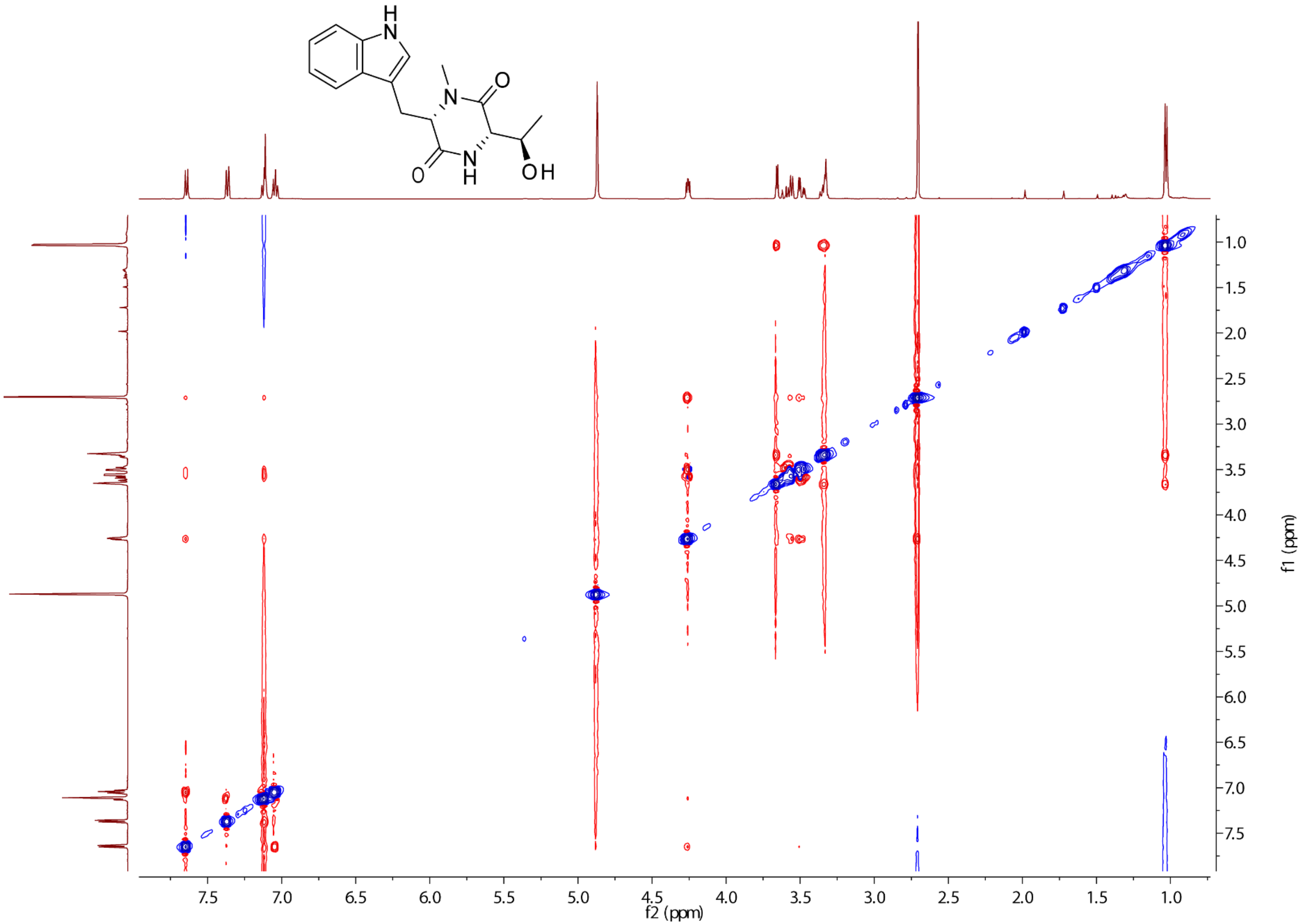

Figure S7. NOESY spectrum (CD_3OD) of **10**.

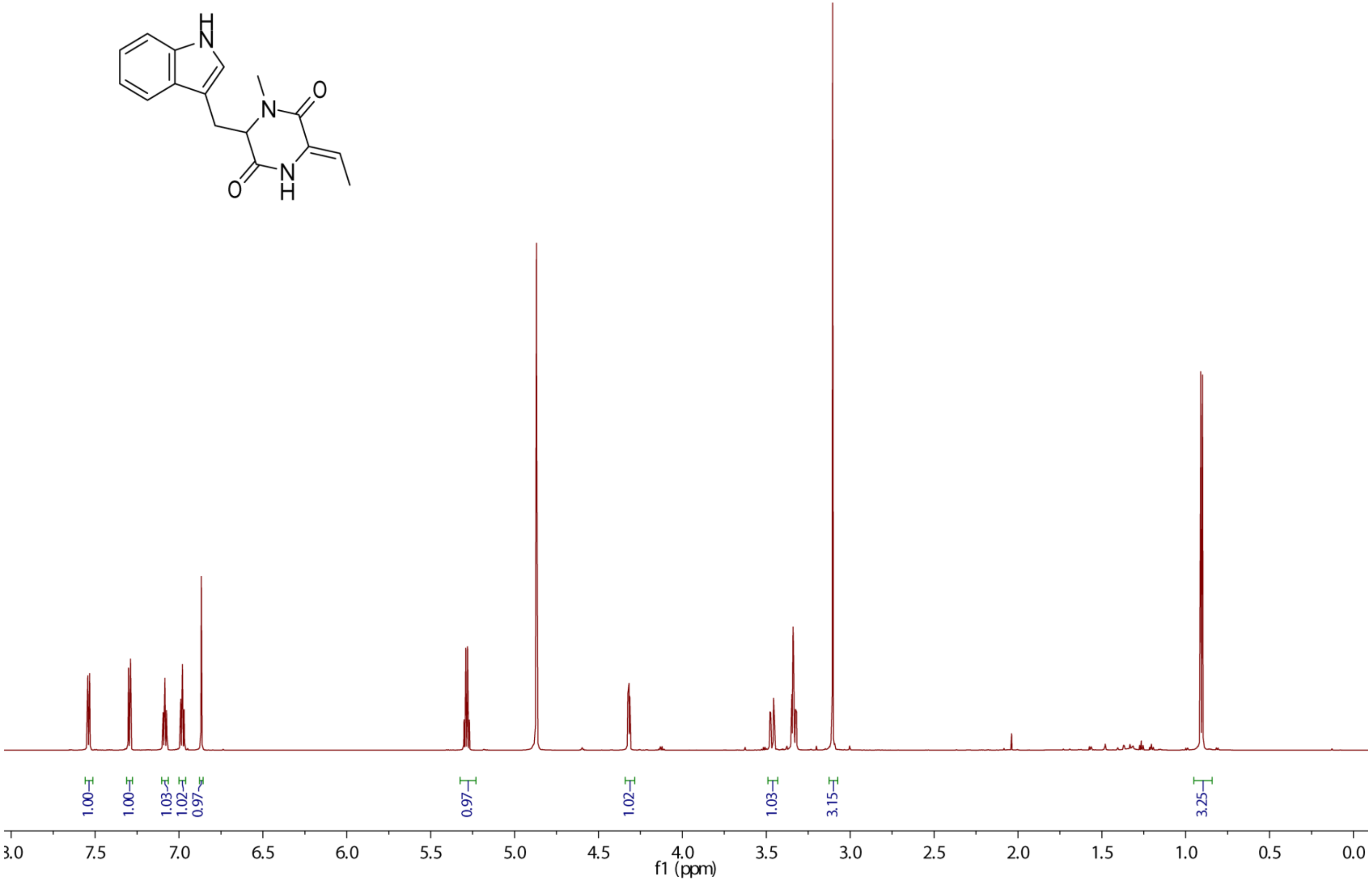

Figure S8. ^{1}H NMR spectrum (700 MHz, CD_3OD) of (*Z*)-**4**.

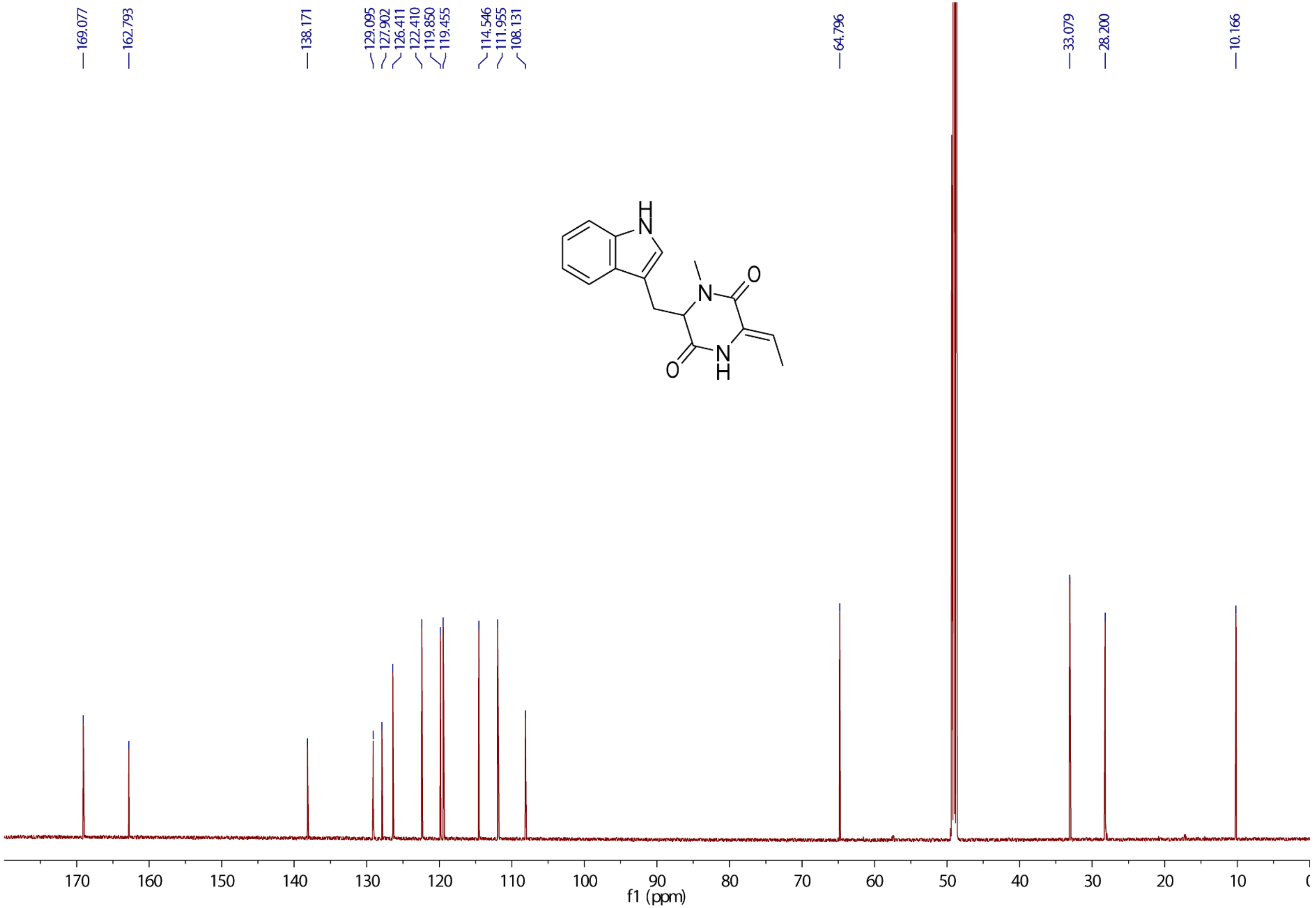

Figure S9. ^{13}C NMR spectrum (176 MHz, CD_3OD) of (*Z*)-**4**.

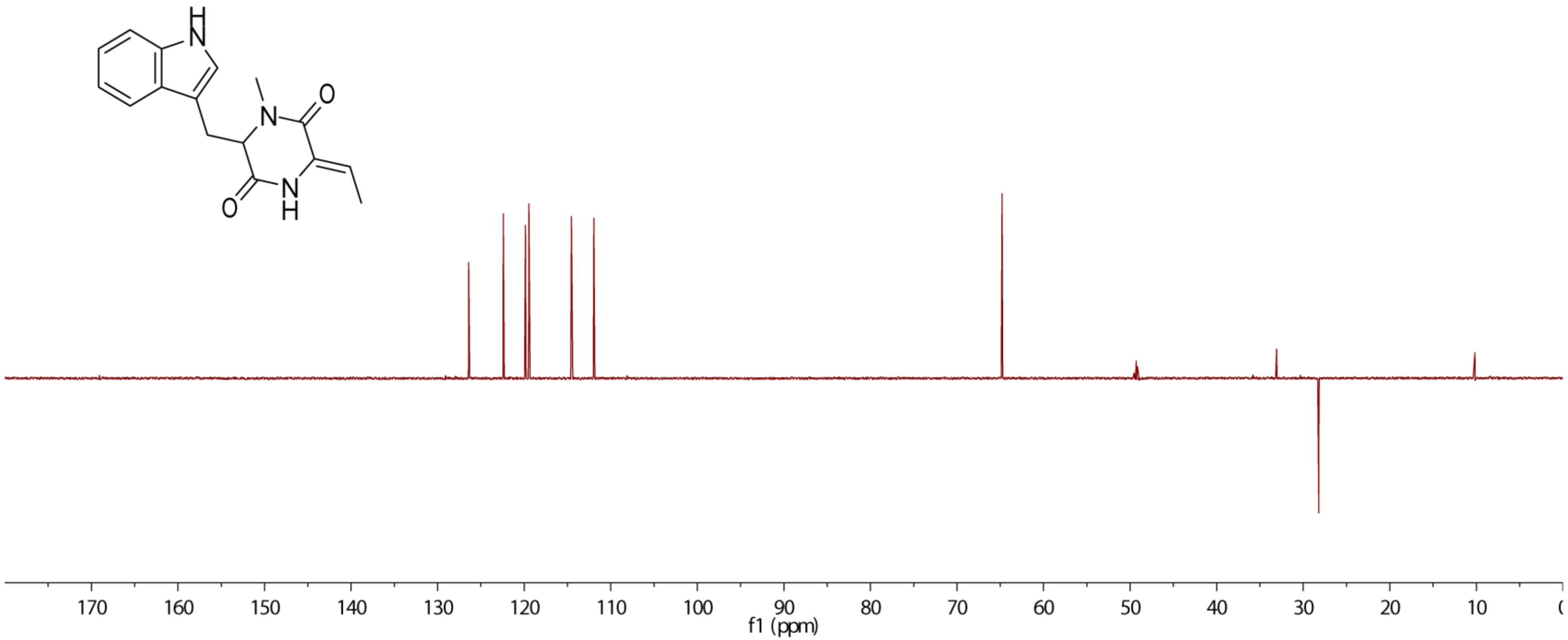

Figure S10. ^{13}C-DEPT-135 NMR spectrum (176 MHz, CD_3OD) of (*Z*)-**4**.

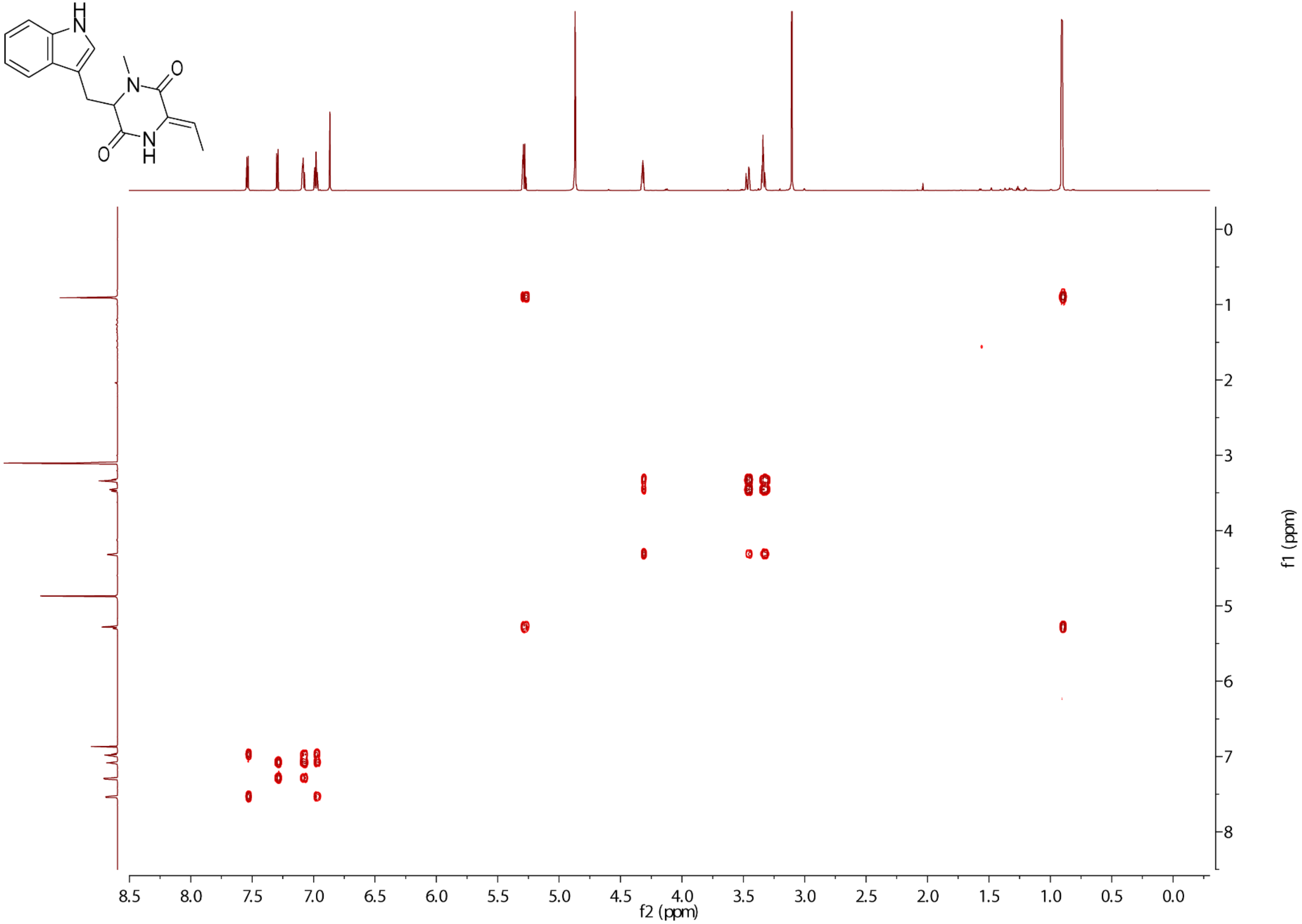

Figure S11. ^{1}H-^{1}H COSY spectrum (700 MHz, CD_3OD) of (*Z*)-**4**.

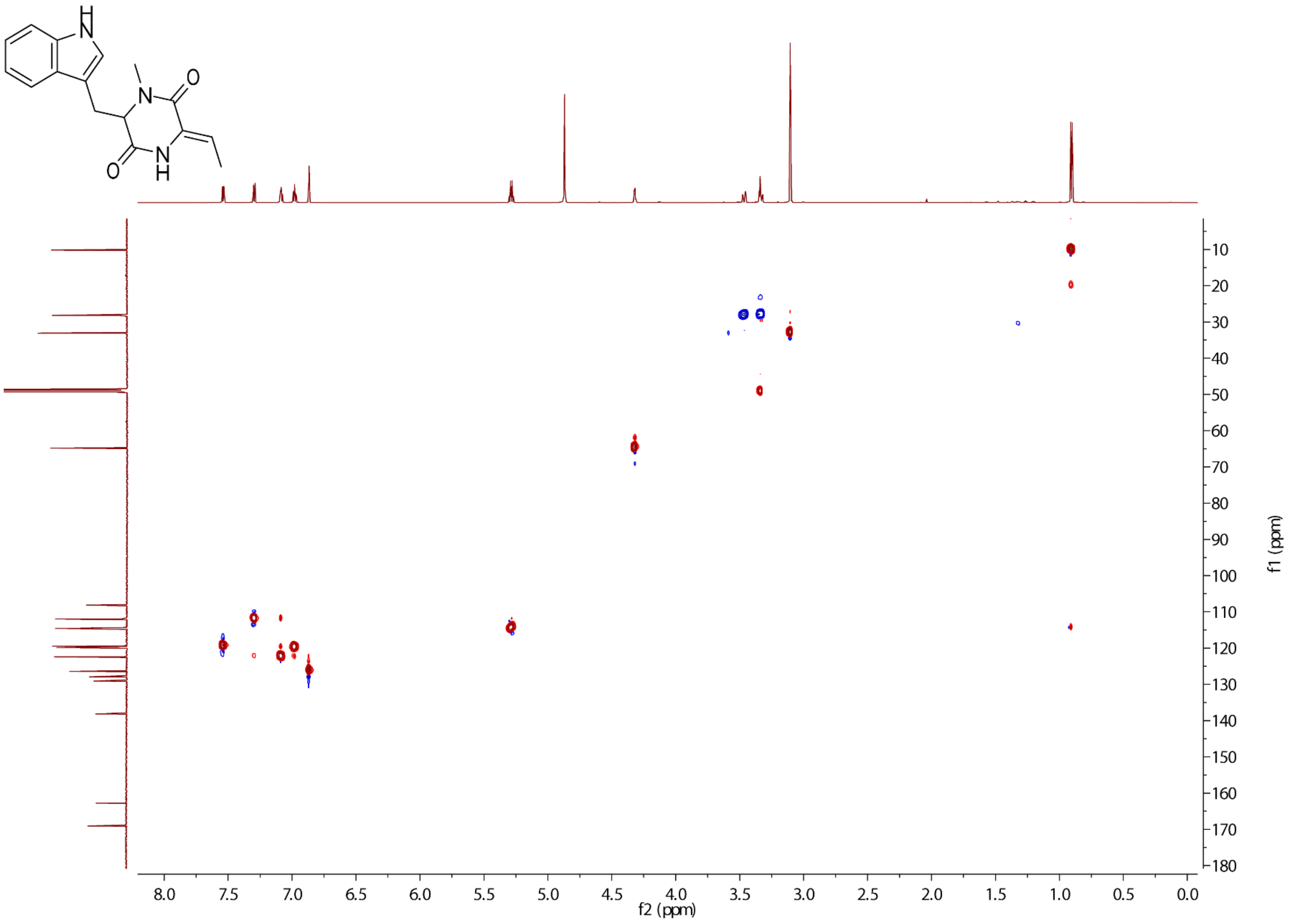

Figure S12. HSQC spectrum (CD_3OD) of (*Z*)-**4**.

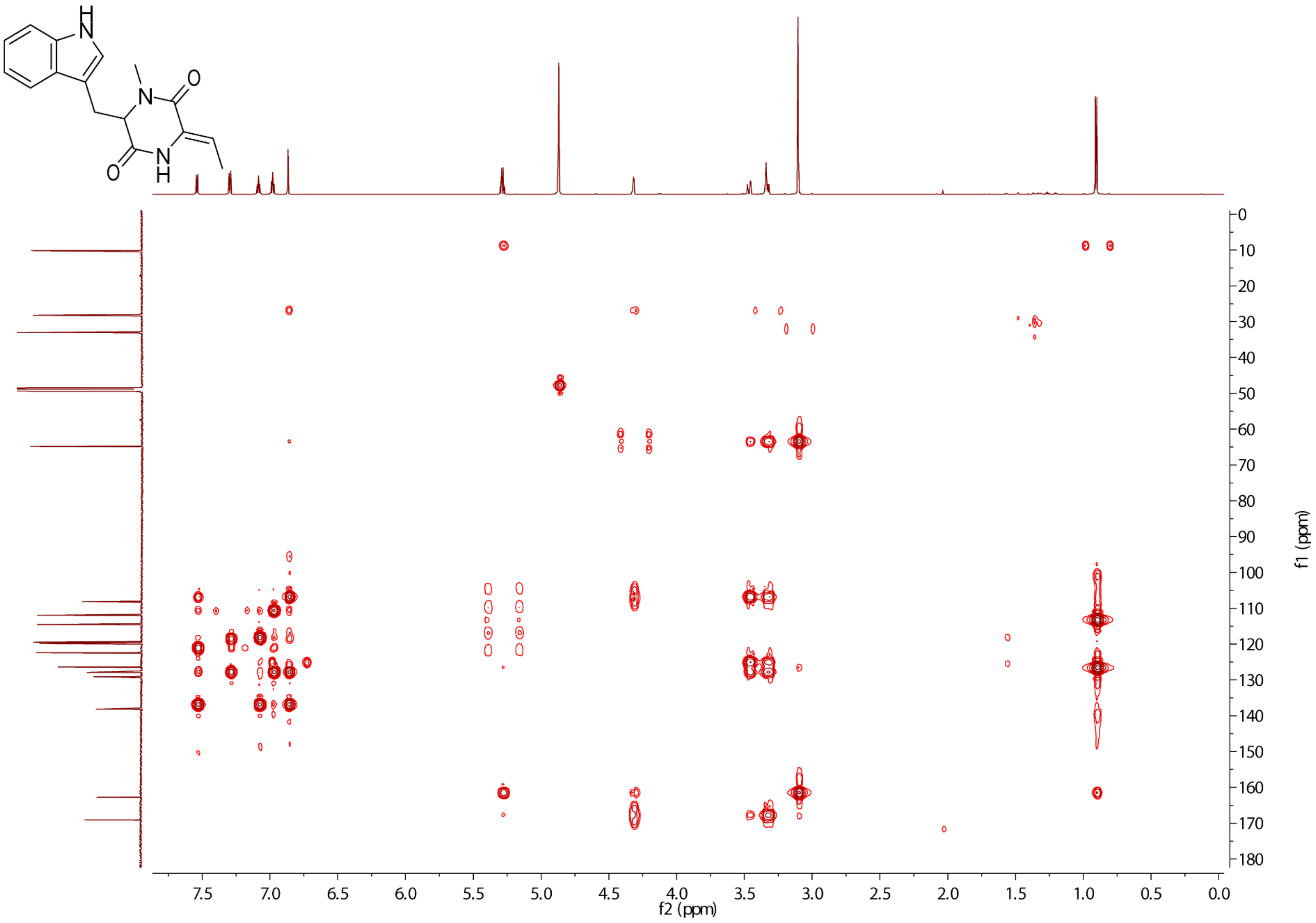

Figure S13. HMBC spectrum (CD_3OD) of (*Z*)-**4**.

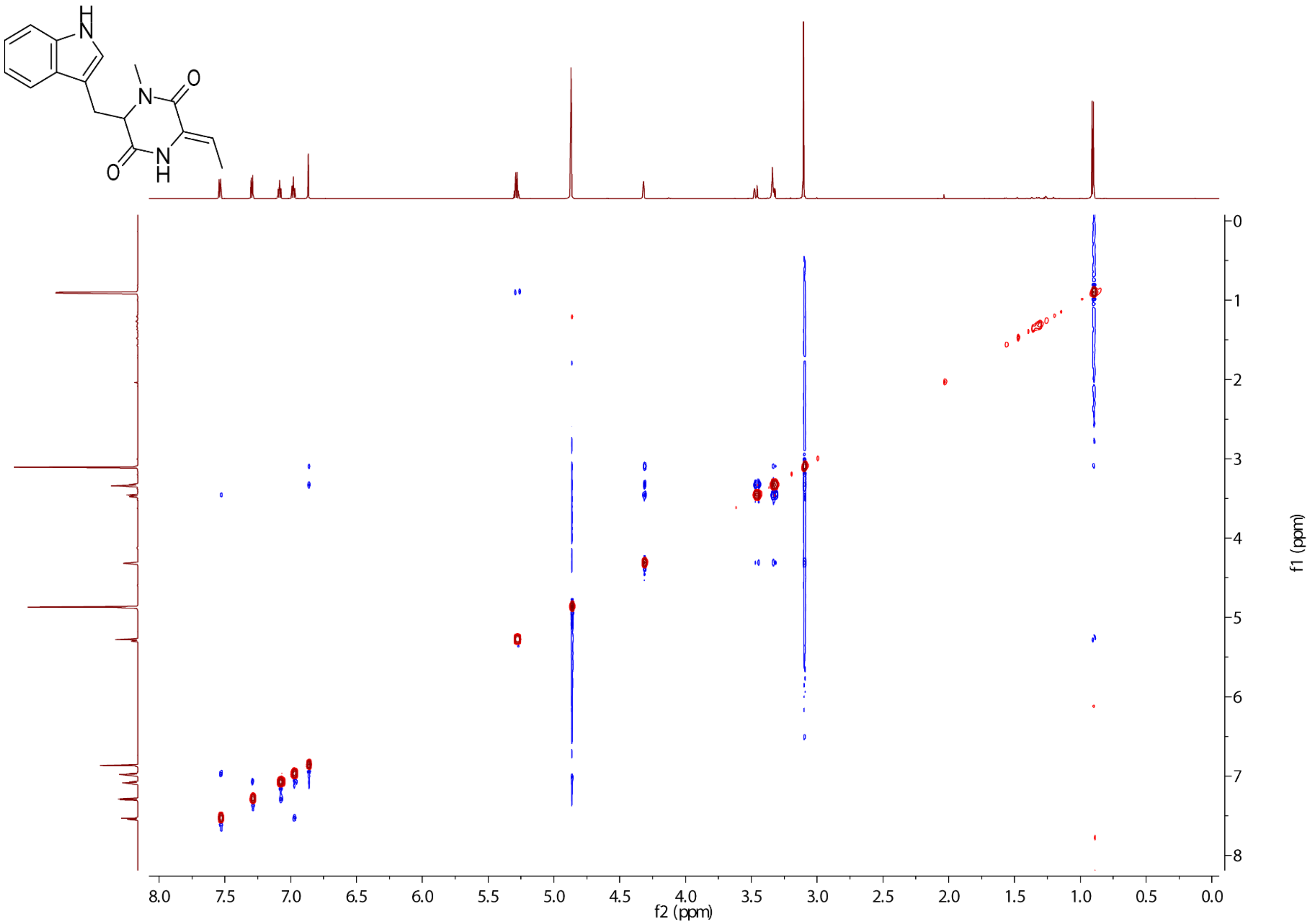

Figure S14. NOESY spectrum (CD_3OD) of (*Z*)-**4** .

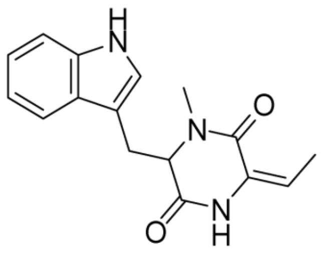

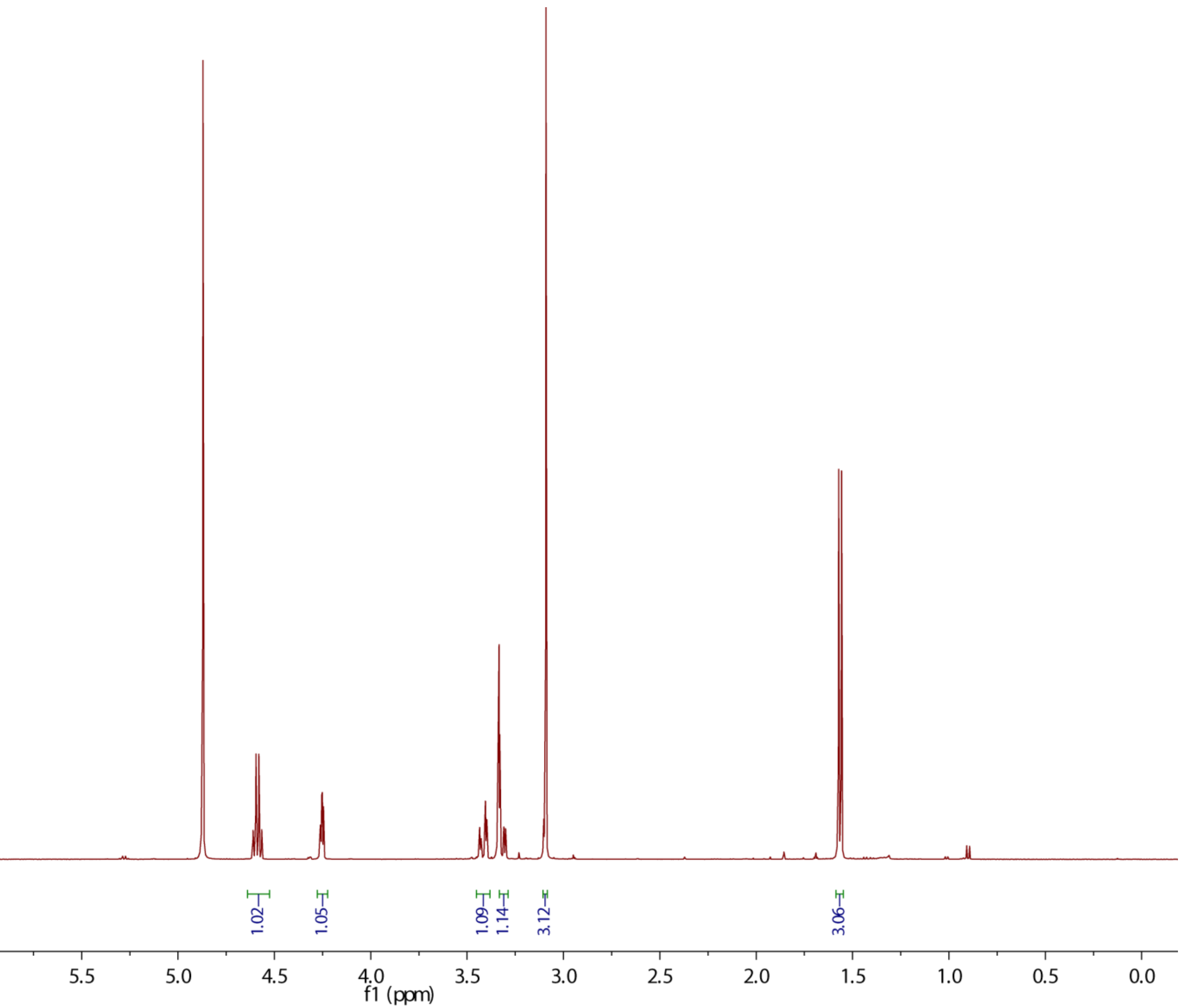

Figure S15. ^1H NMR spectrum (500 MHz, CD_3OD) of (*E*)-**4**

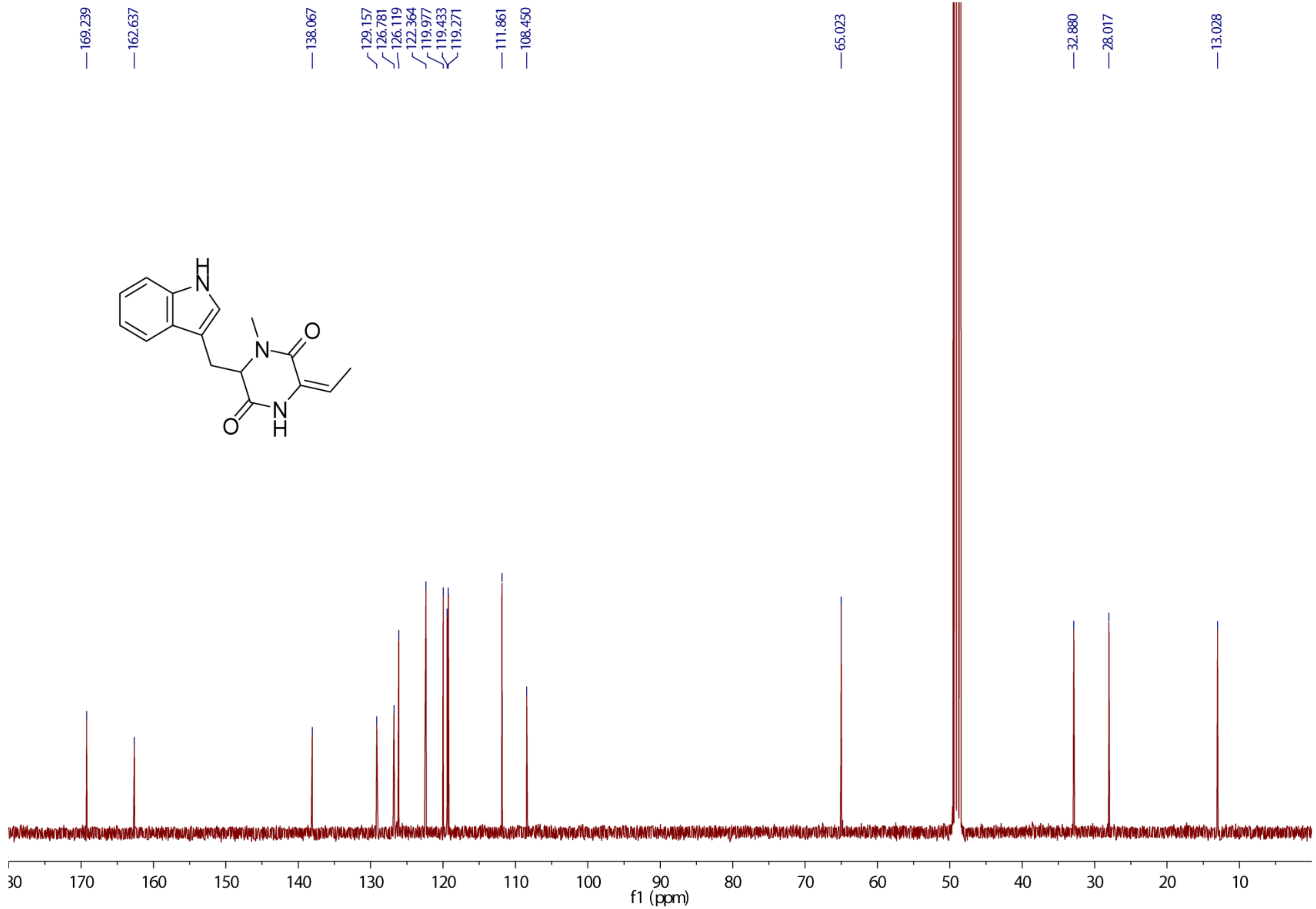

Figure S16. ^{13}C NMR spectrum (126 MHz, CD_3OD) of (*E*)-**4**.

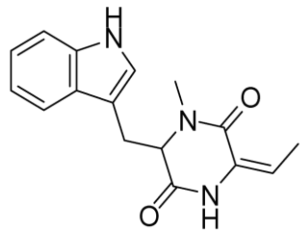

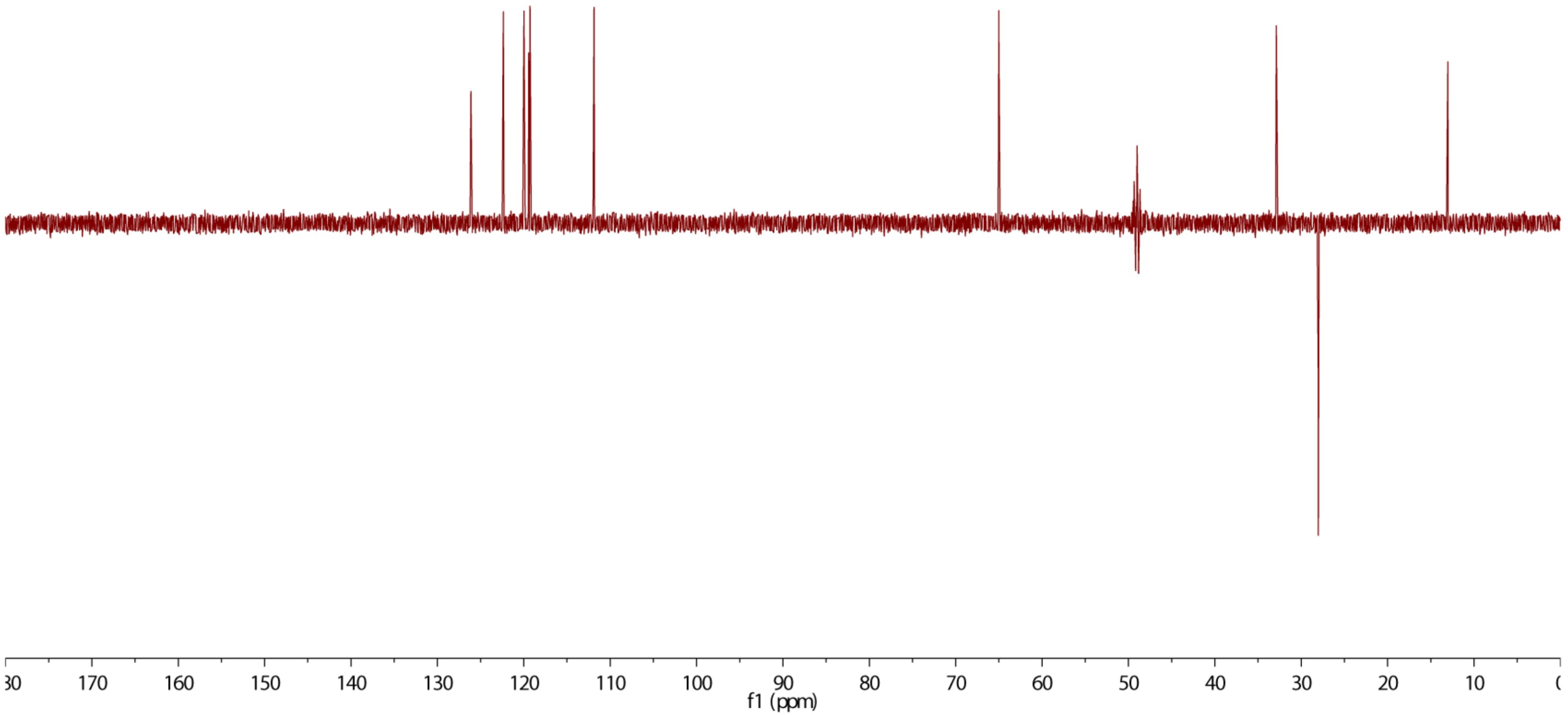

Figure S17. ^{13}C-DEPT-135 NMR spectrum (126 MHz, CD_3OD) of (*E*)-**4**.

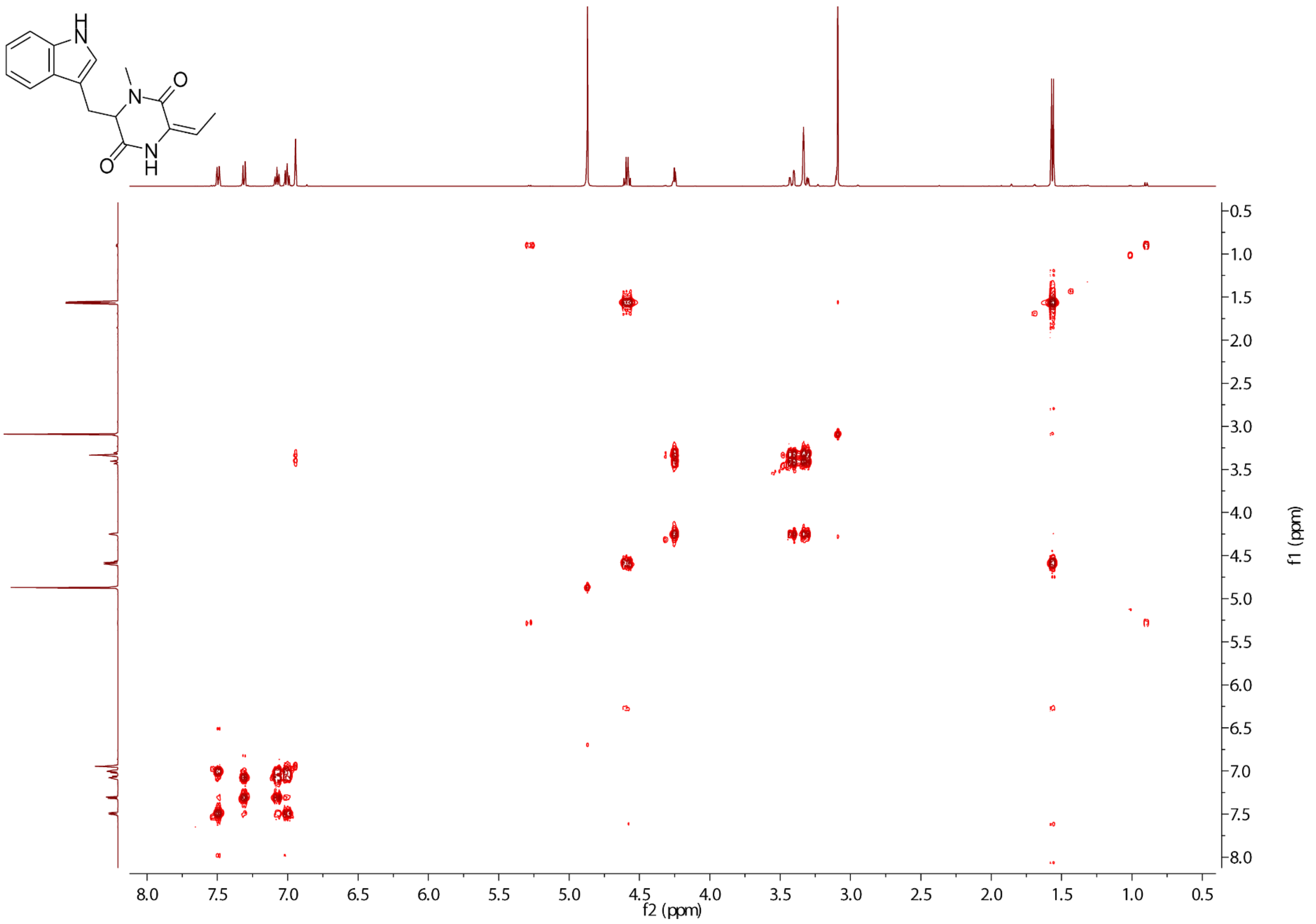

Figure S18. ^1H-^1H COSY spectrum (500 MHz, CD_3OD) of (*E*)-**4**.

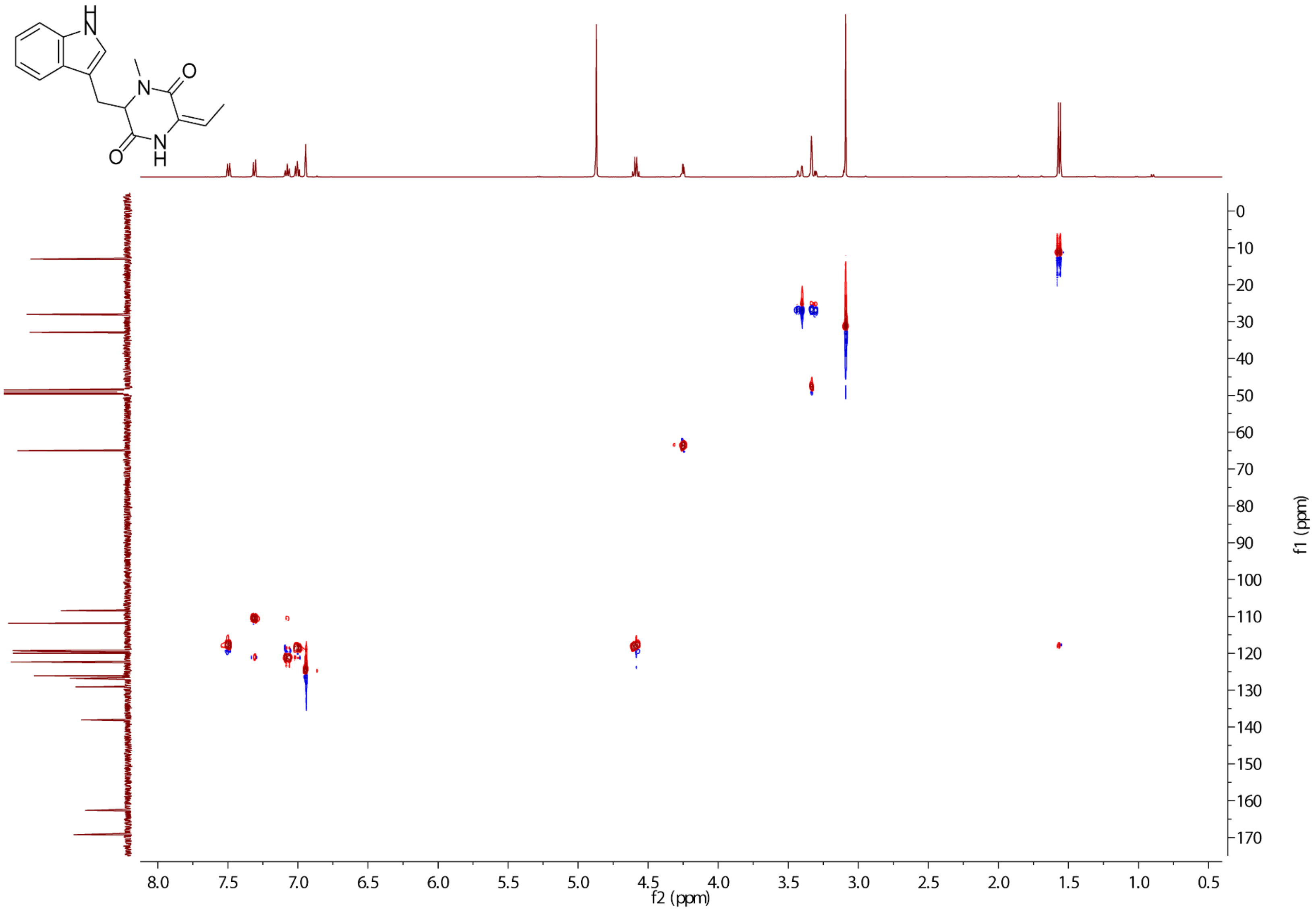

Figure S19. HSQC spectrum (CD_3OD) of (*E*)-**4**.

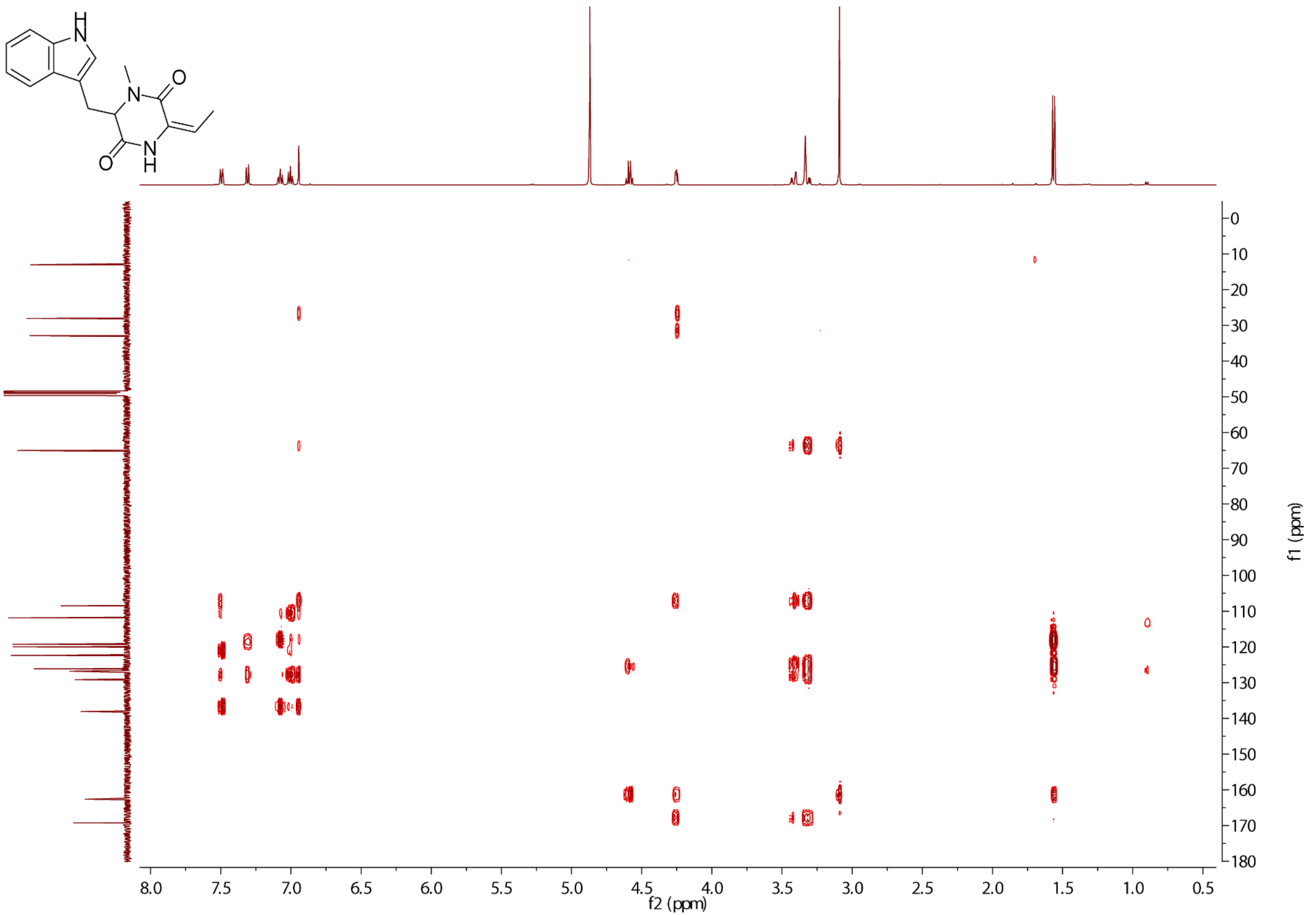

Figure S20. HMBC spectrum (CD_3OD) of (*E*)-**4**.

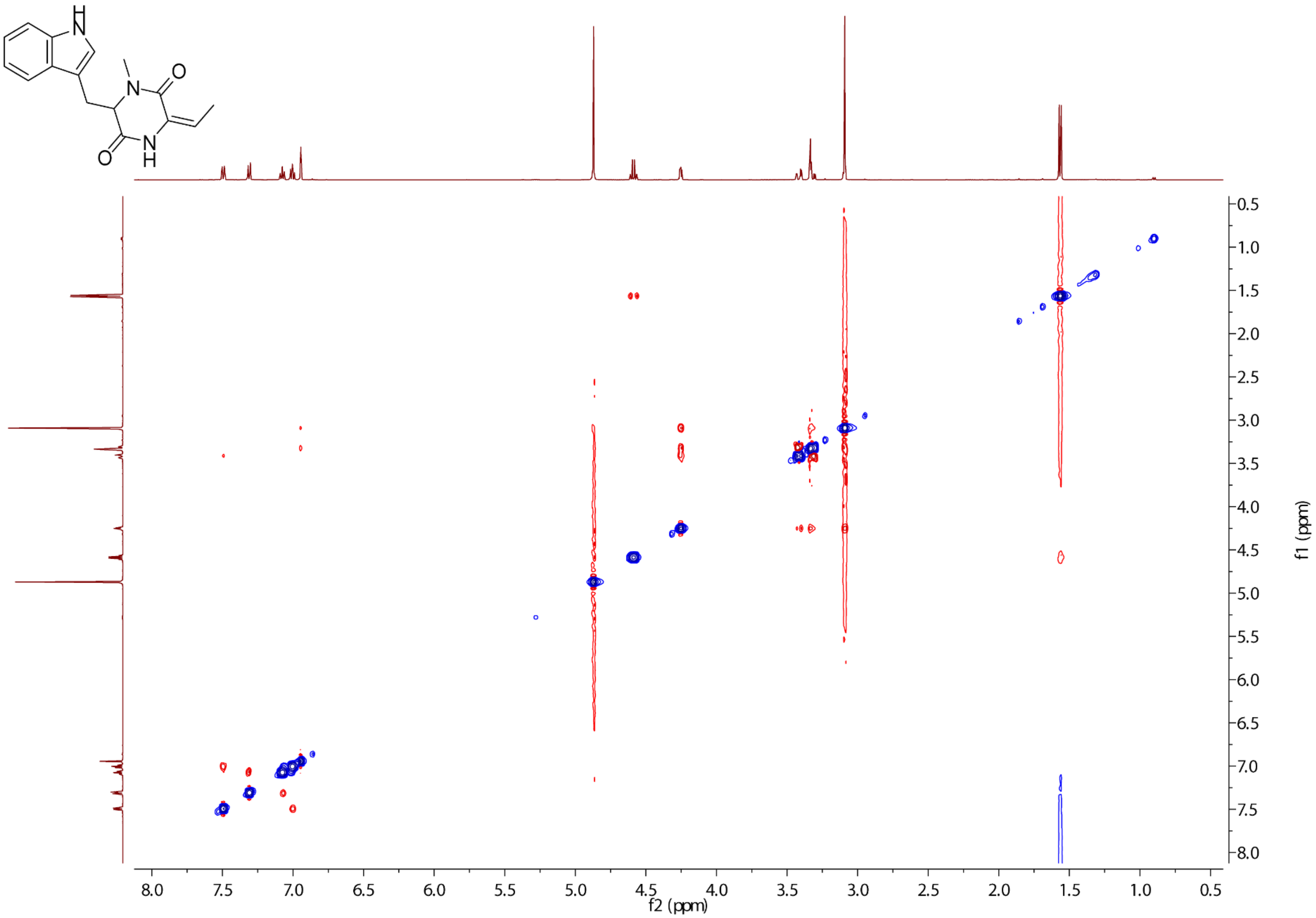

Figure S21. NOESY spectrum (CD3OD) of (*E*)-**4** .

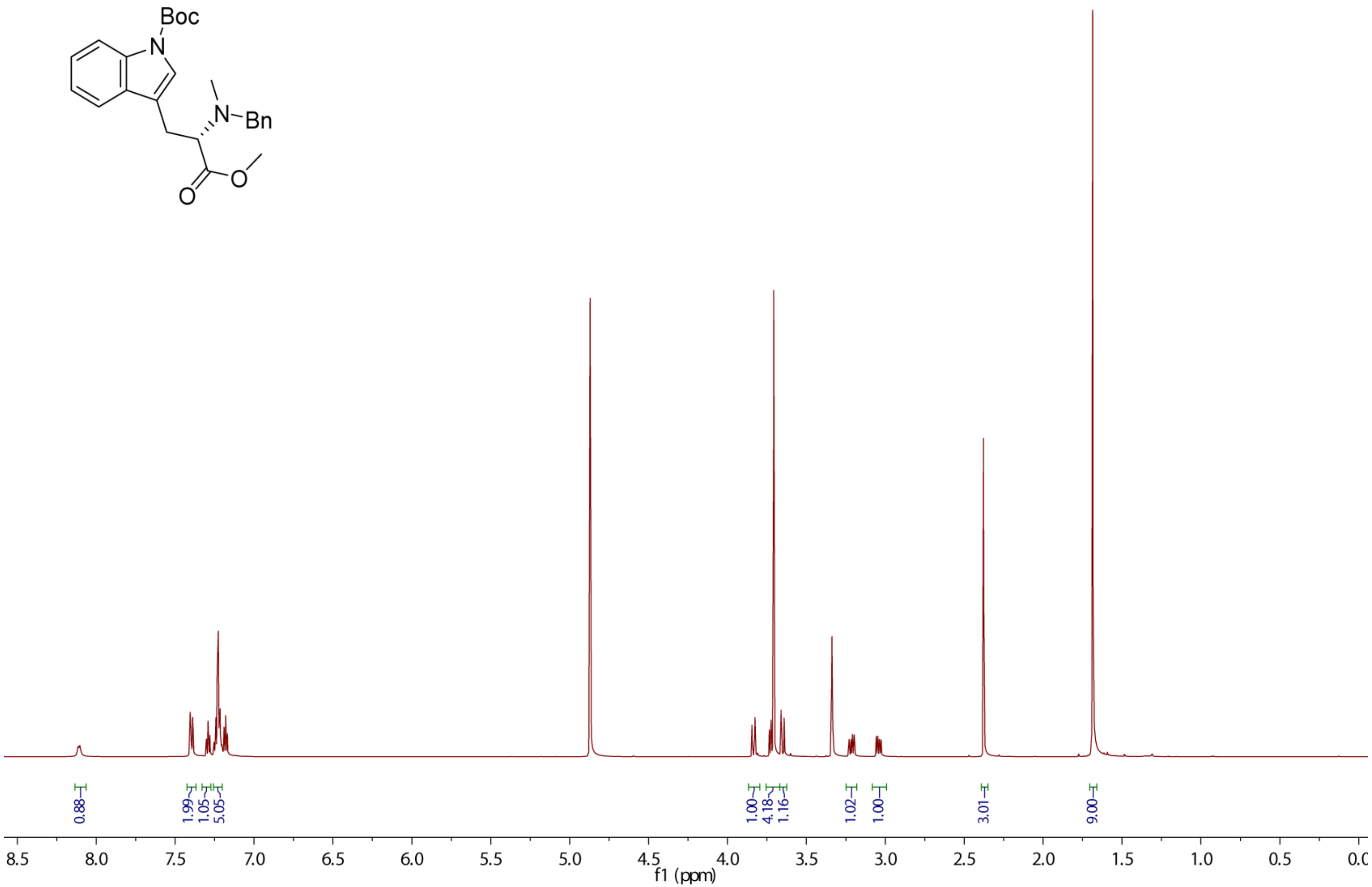

Figure S22. ^{1}H NMR spectrum (700 MHz, CD_3OD) of **11**.

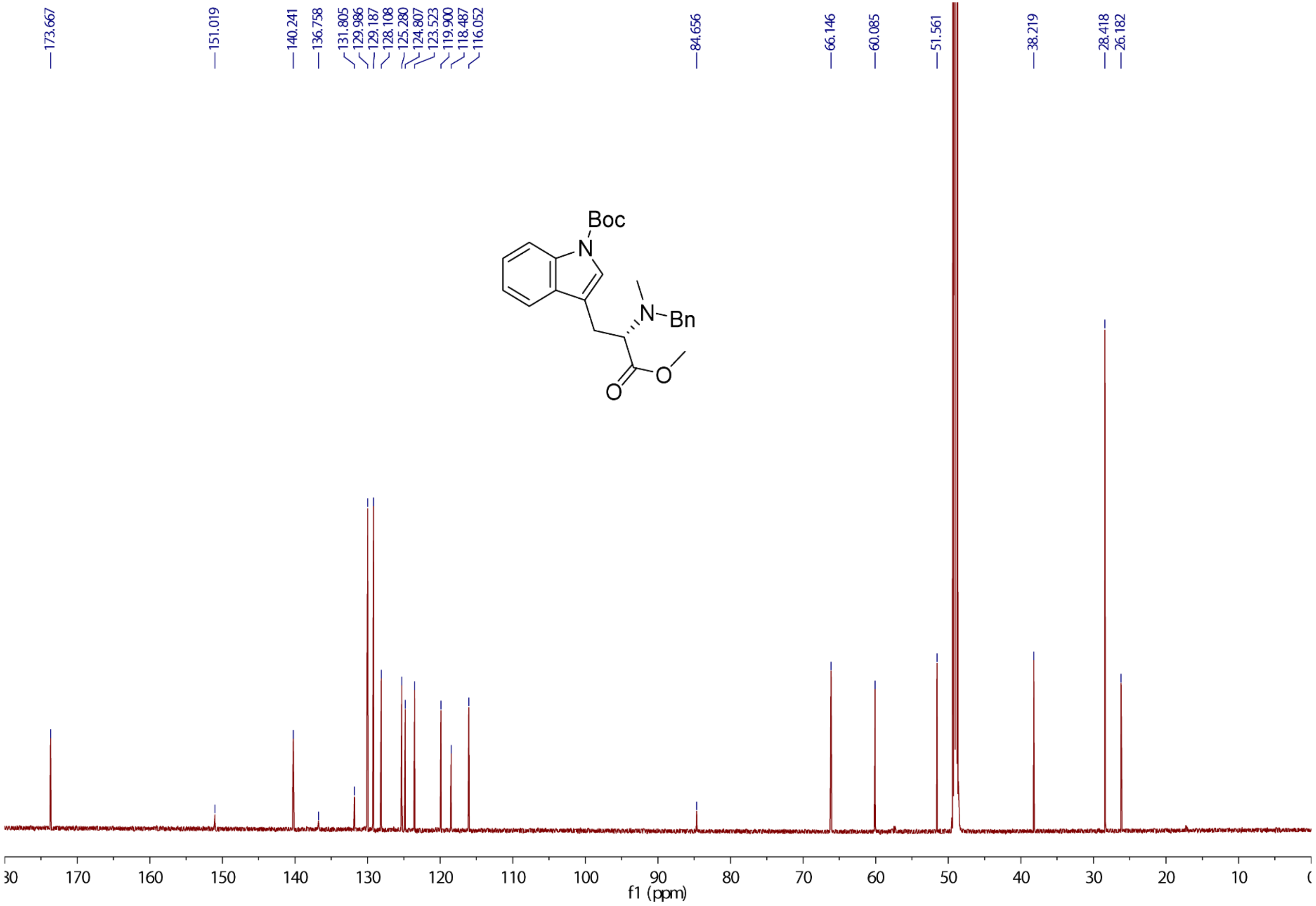

Figure S23. ^{13}C NMR spectrum (176 MHz, CD_3OD) of **11**.

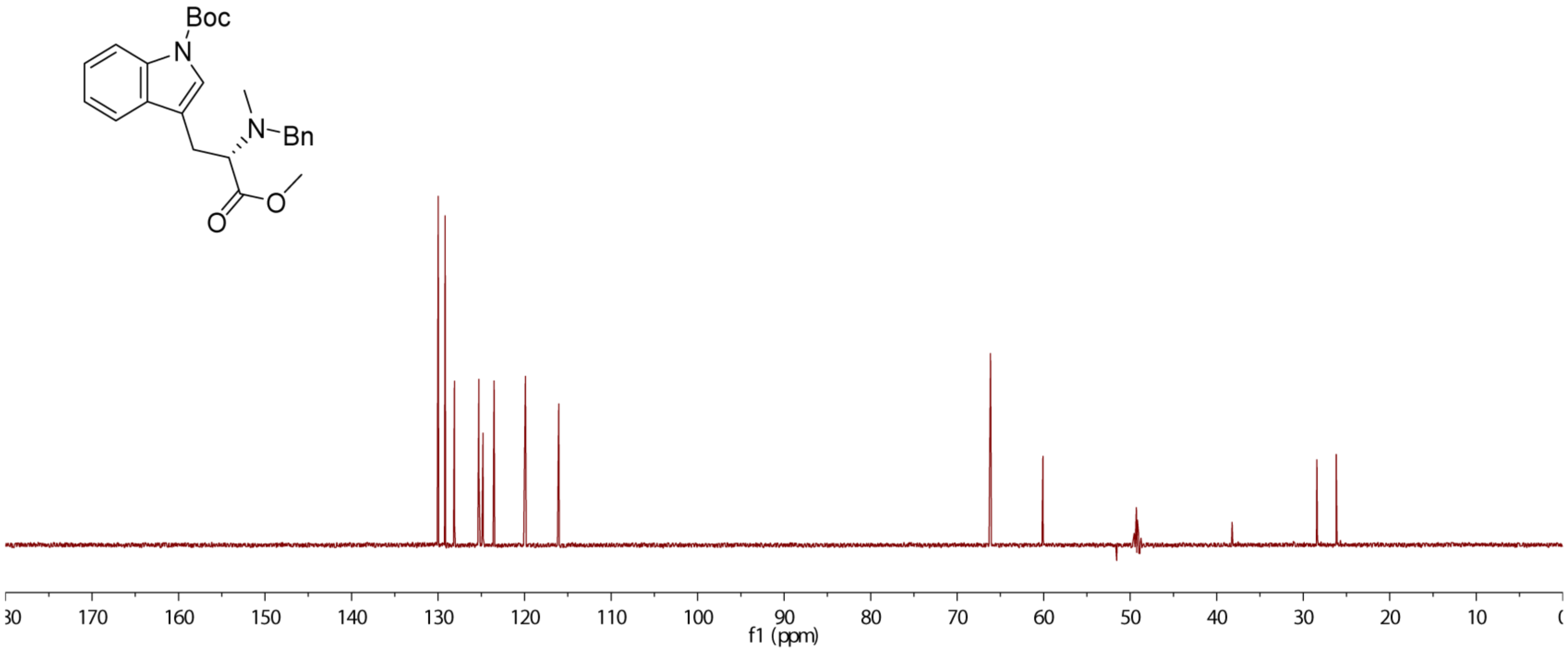

Figure S24. ^{13}C-DEPT-135 NMR spectrum (176 MHz, CD_3OD) of **11**.

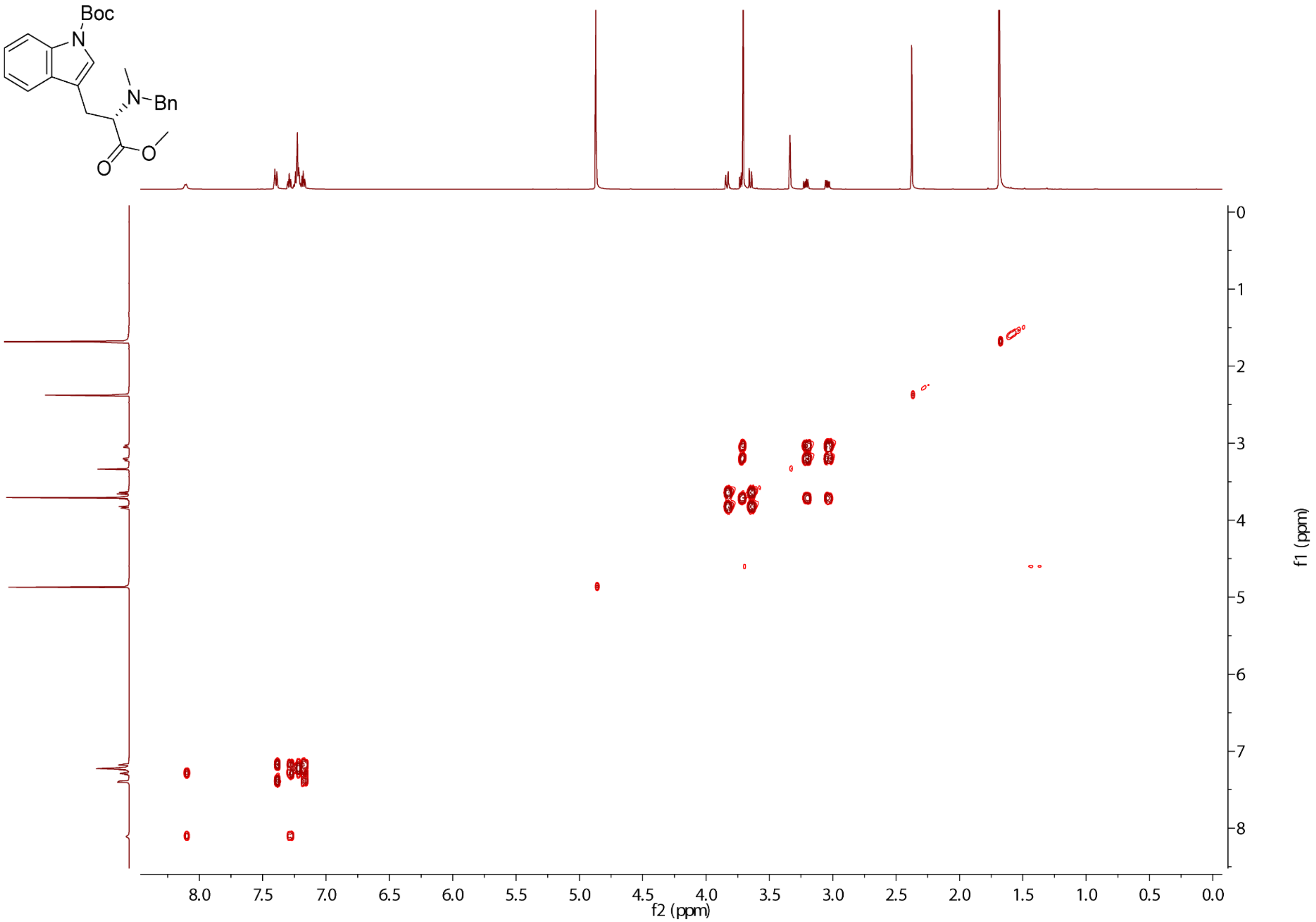

Figure S25. ^1H-^1H COSY spectrum (700 MHz, CD_3OD) of **11**.

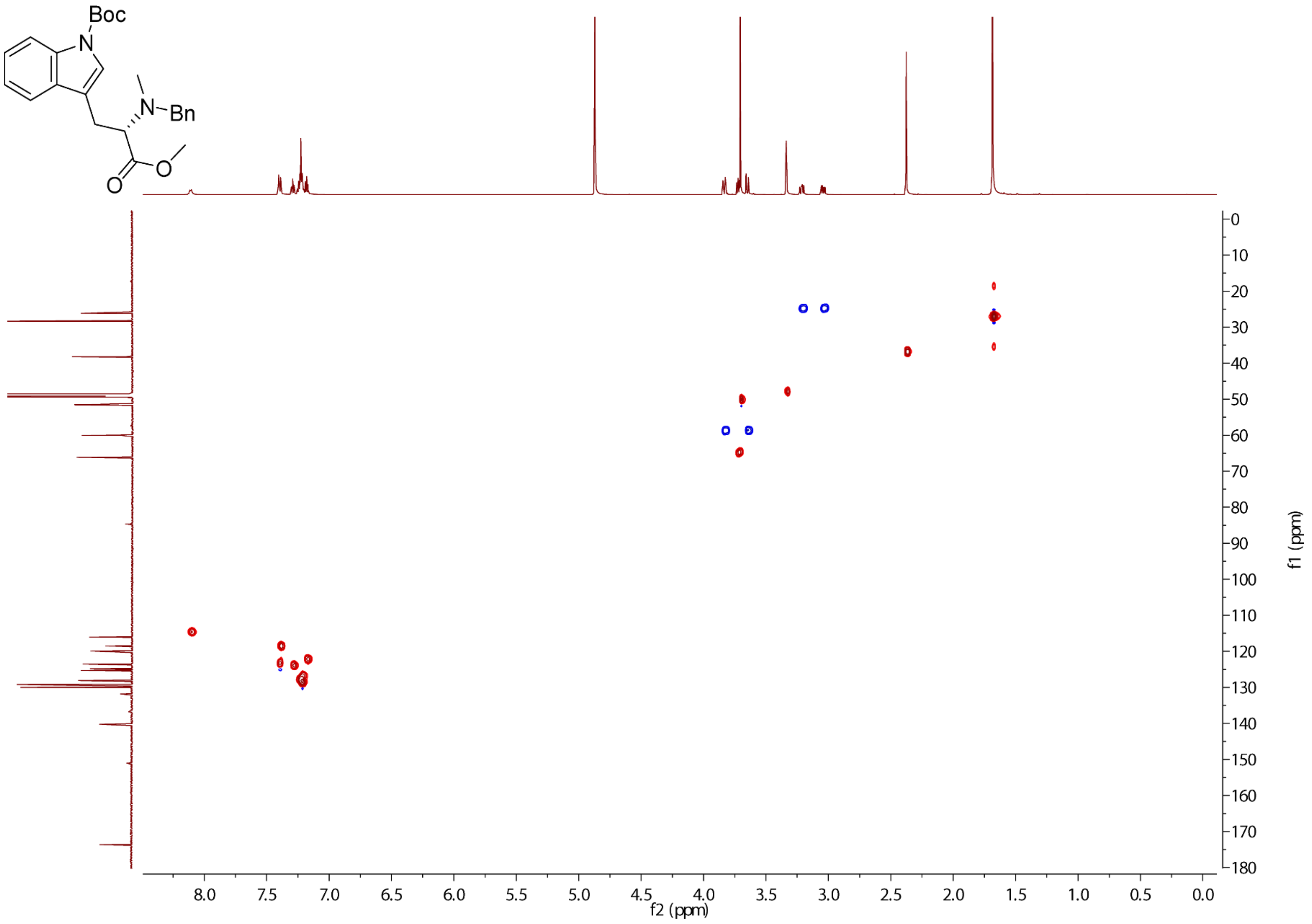

Figure S26. HSQC spectrum (CD_3OD) of **11**.

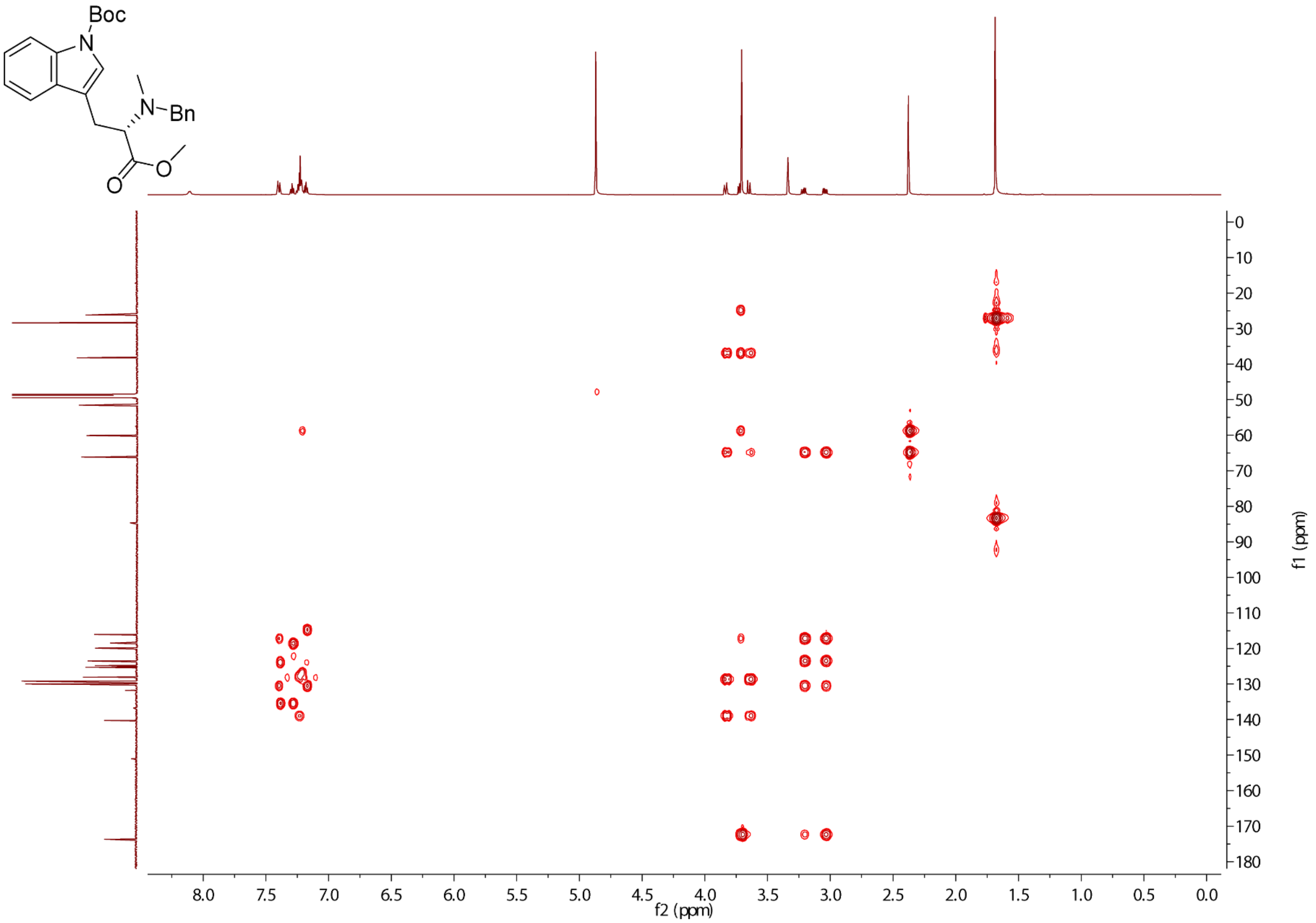

Figure S27. HMBC spectrum (CD_3OD) of **11**.

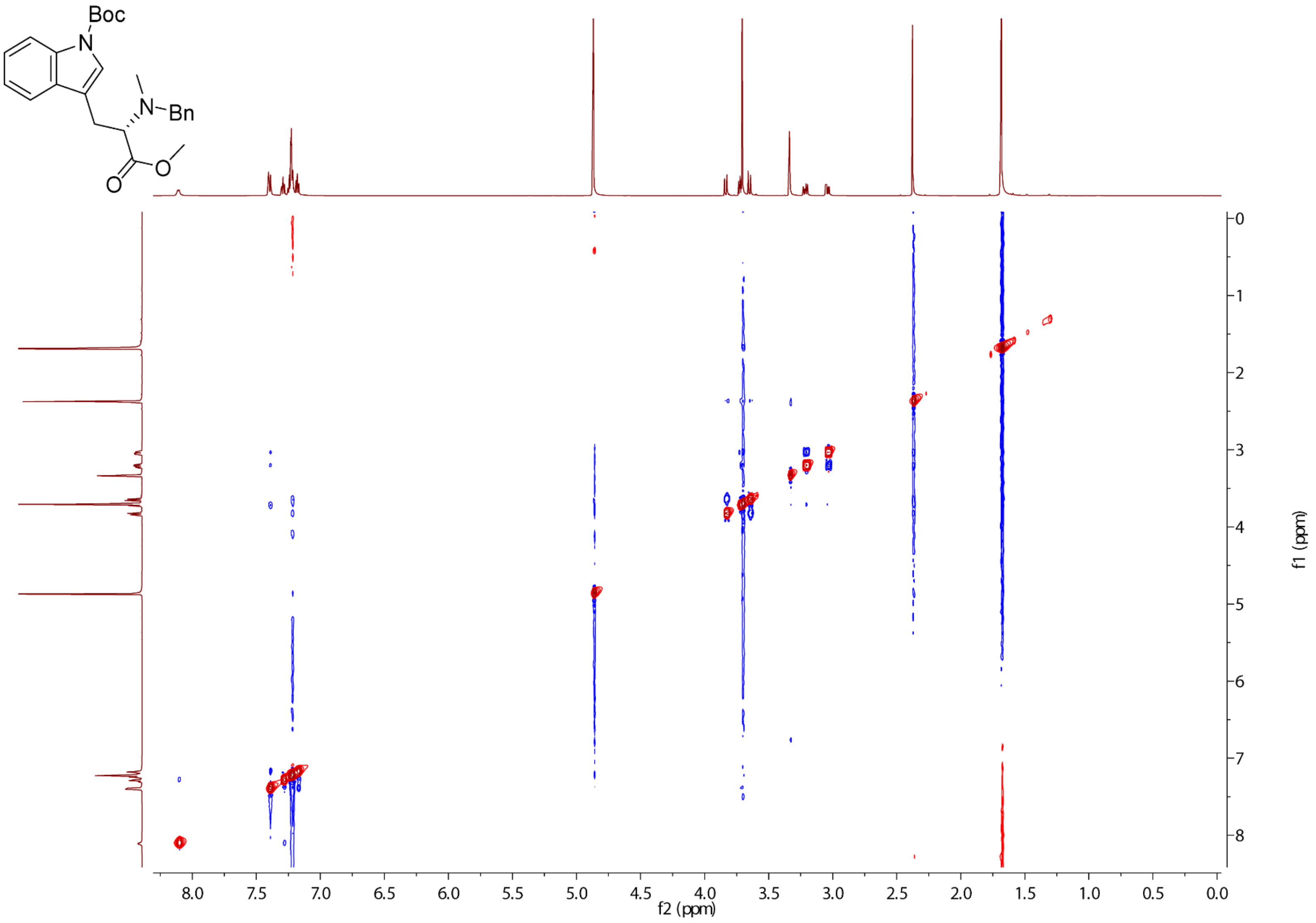

Figure S28. NOESY spectrum (CD_3OD) of **11** .

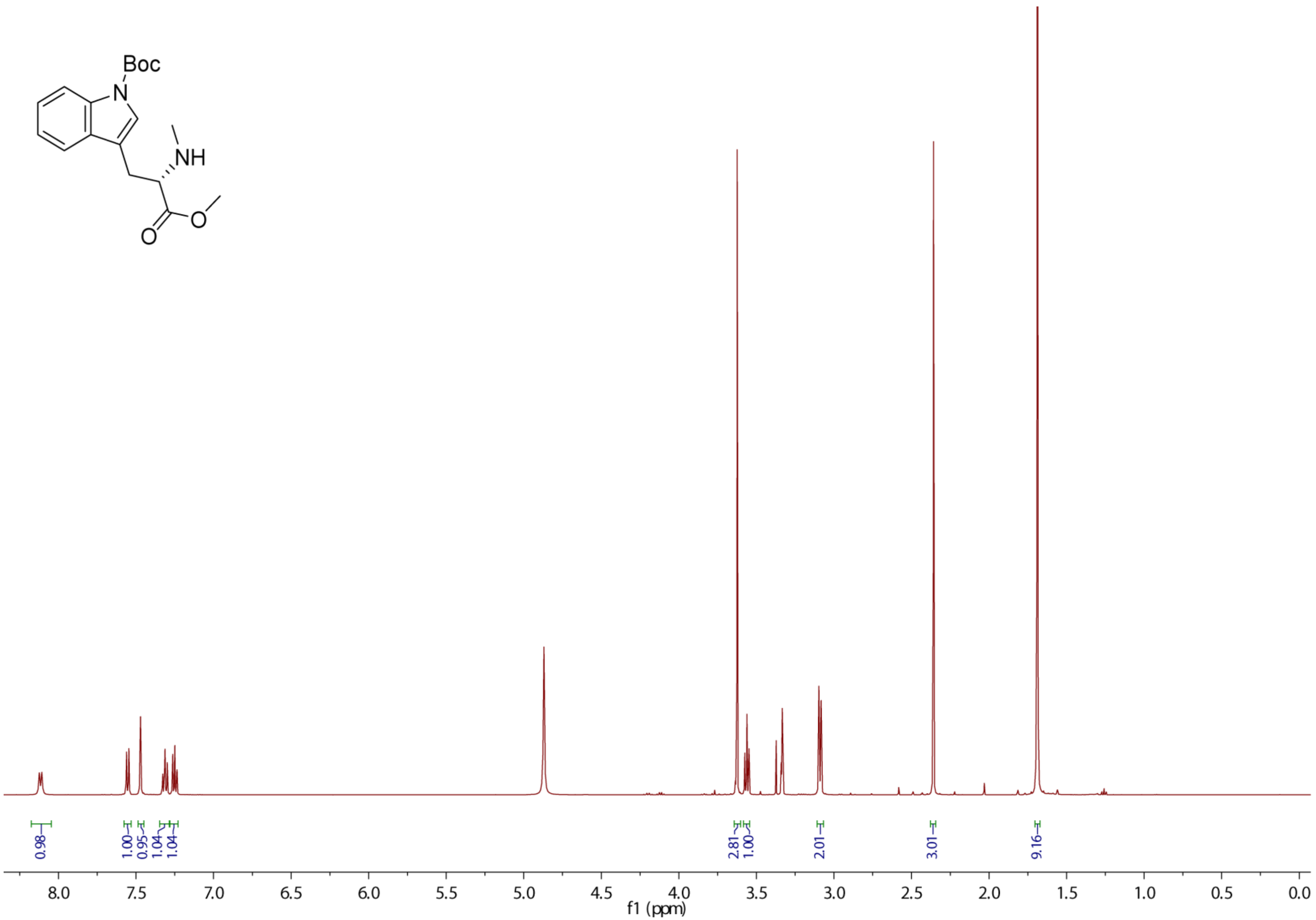

Figure S29. ^{1}H NMR spectrum (500 MHz, CD3OD) of **12**.

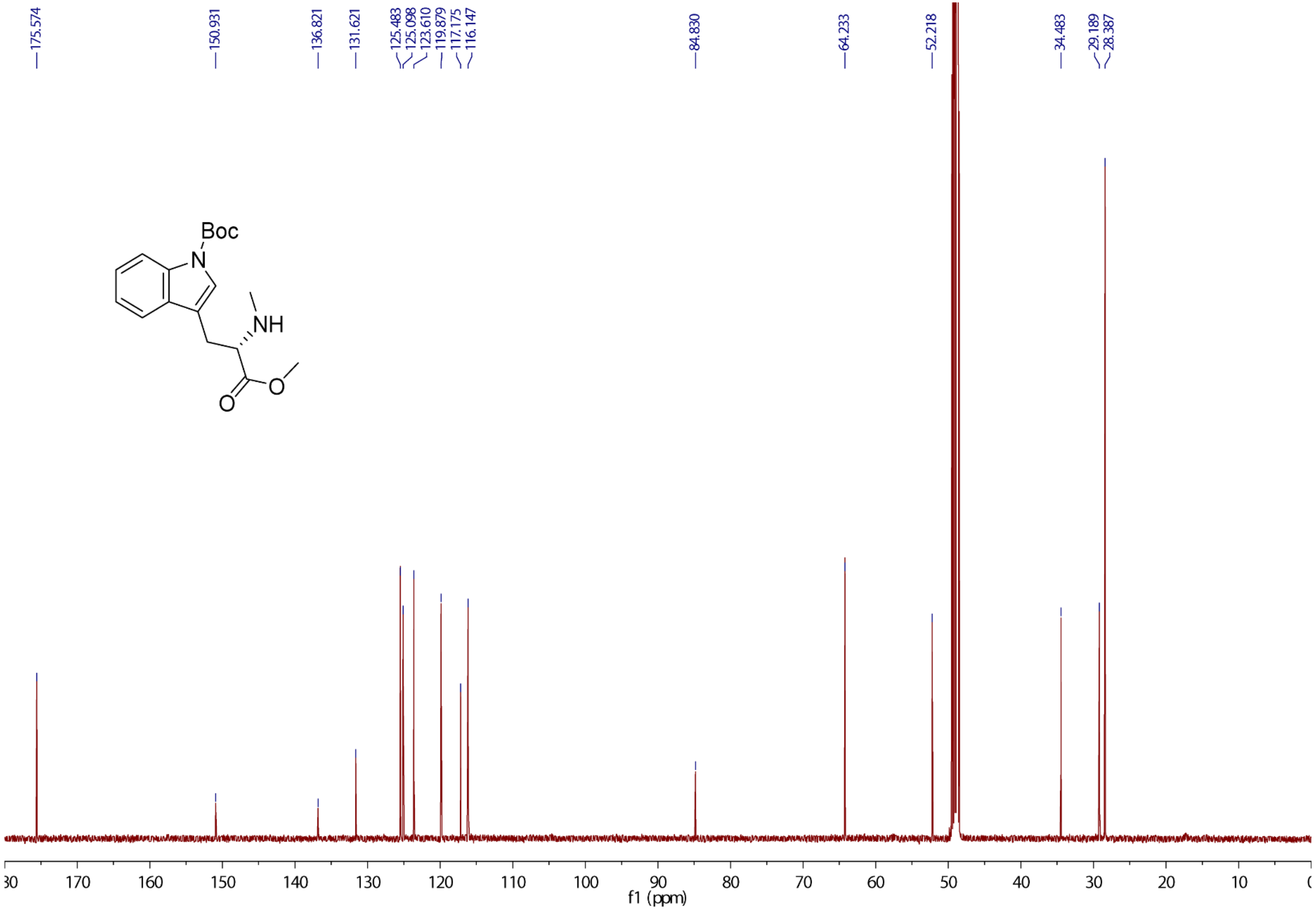

Figure S30. ^{13}C NMR spectrum (126 MHz, CD_3OD) of **12**.

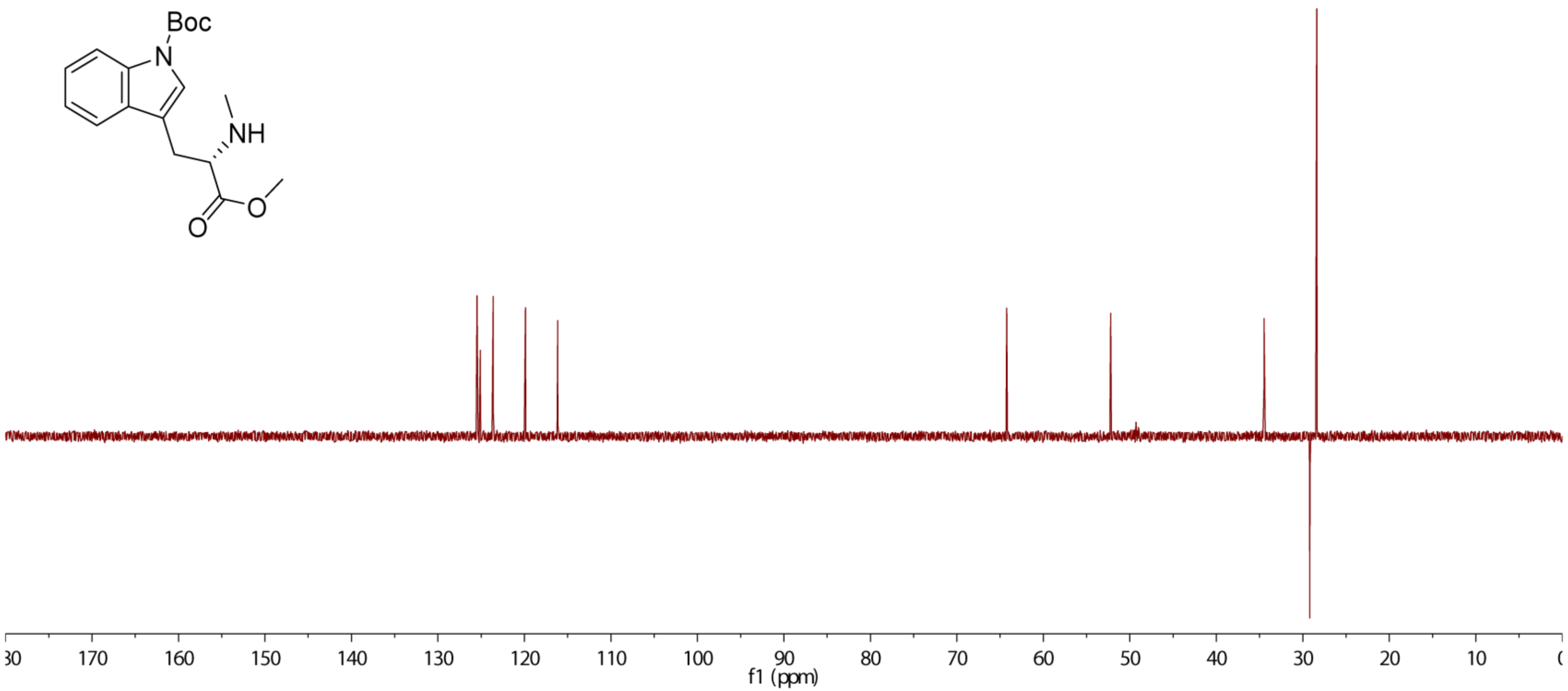

Figure S31. ^{13}C-DEPT-135 NMR spectrum (126 MHz, CD_3OD) of **12**.

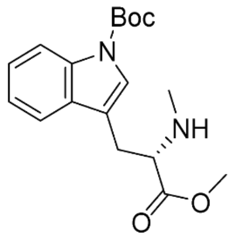

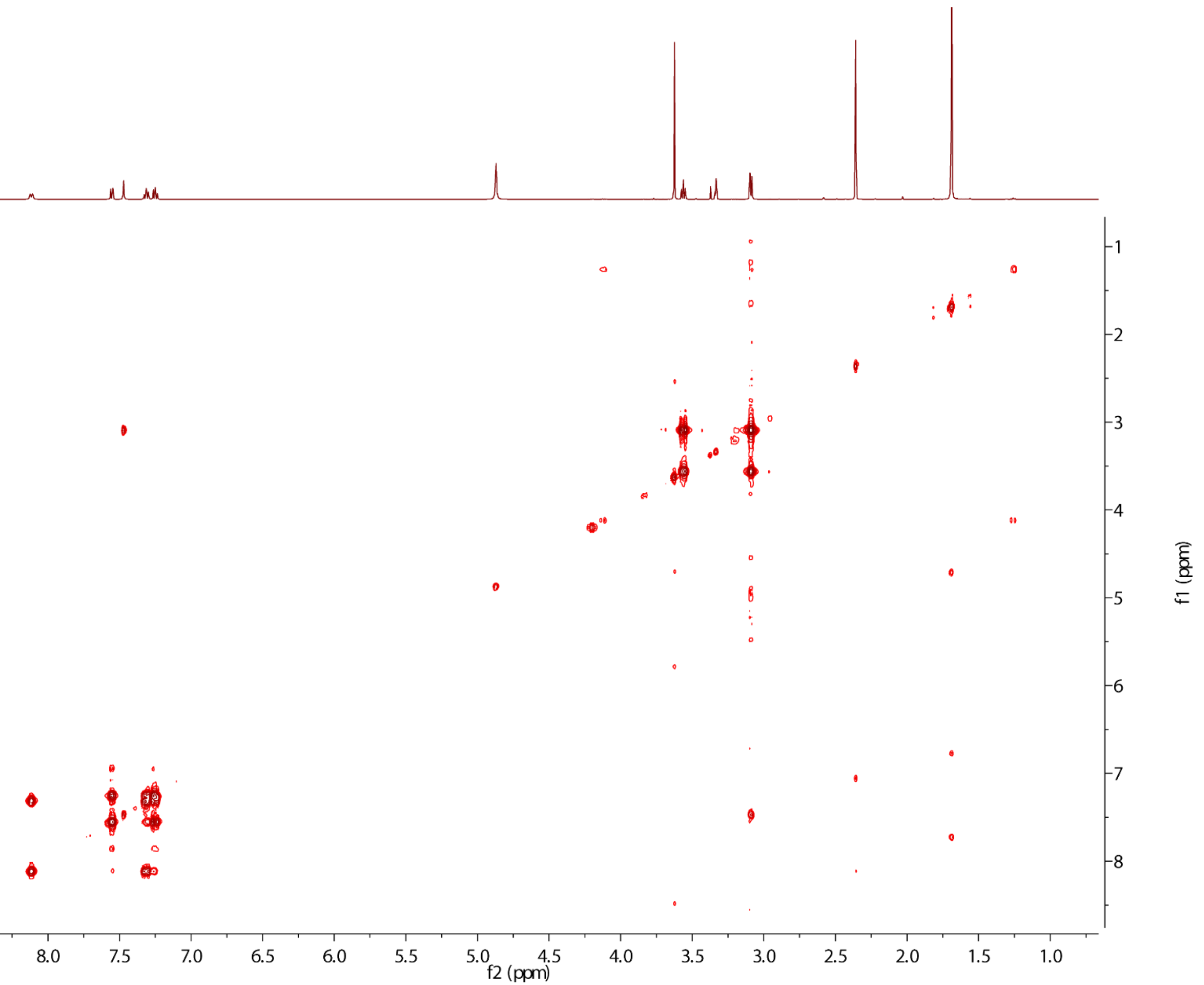

Figure S32. ^{1}H-^{1}H COSY spectrum (500 MHz, CD_3OD) of **12**.

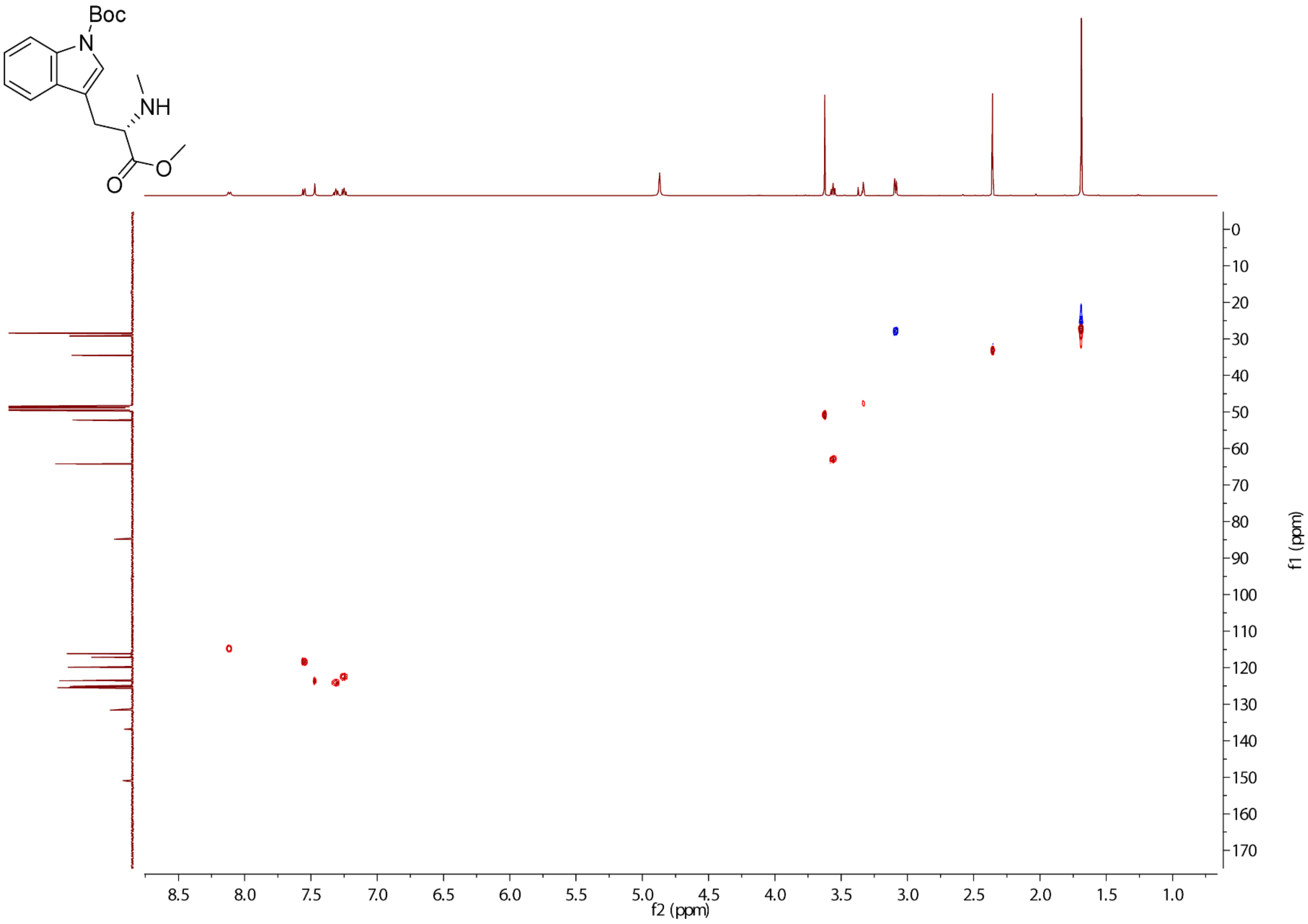

Figure S33. HSQC P (CD_3OD) of **12**.

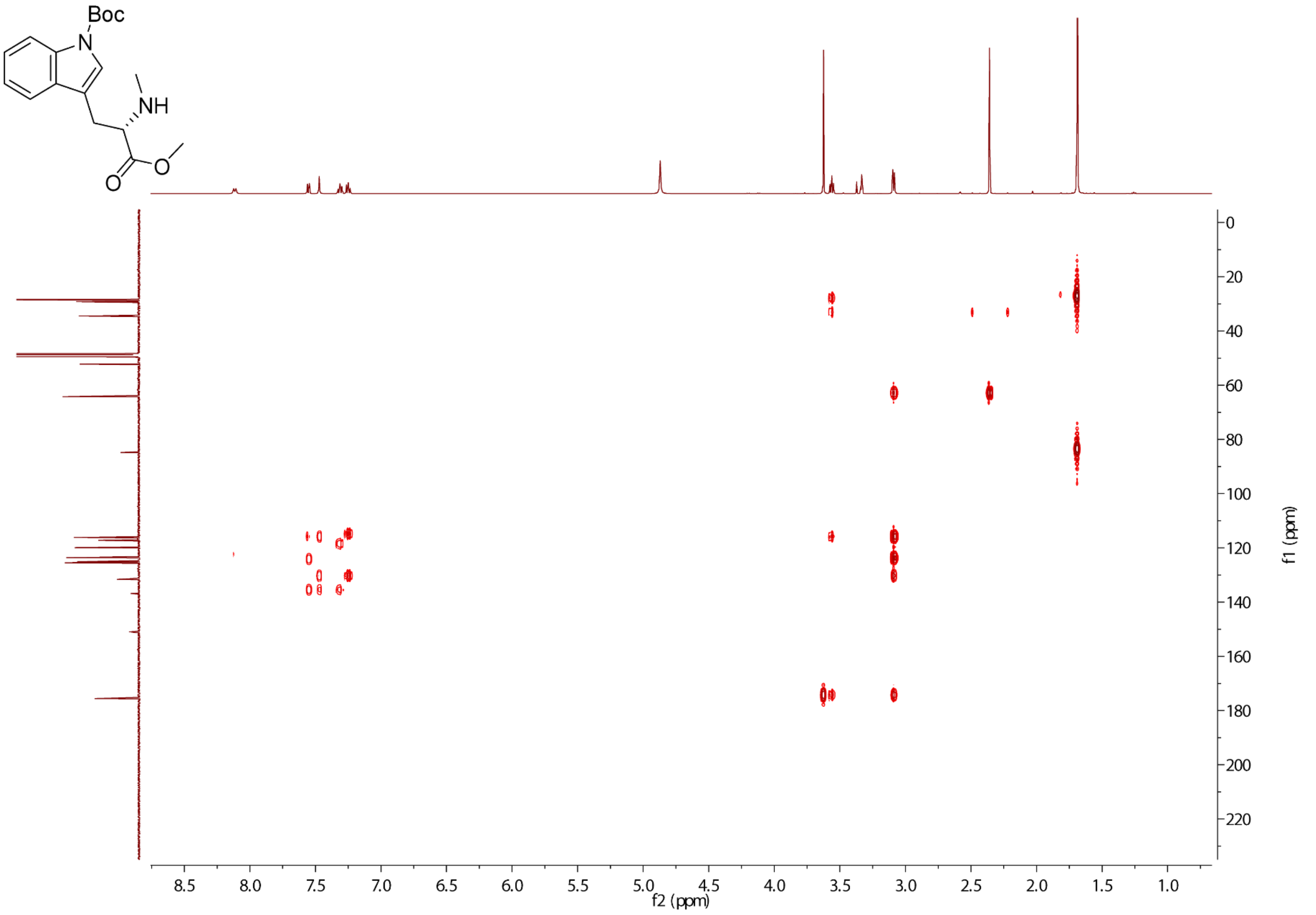

Figure S34. HMBC spectrum (CD_3OD) of **12**.

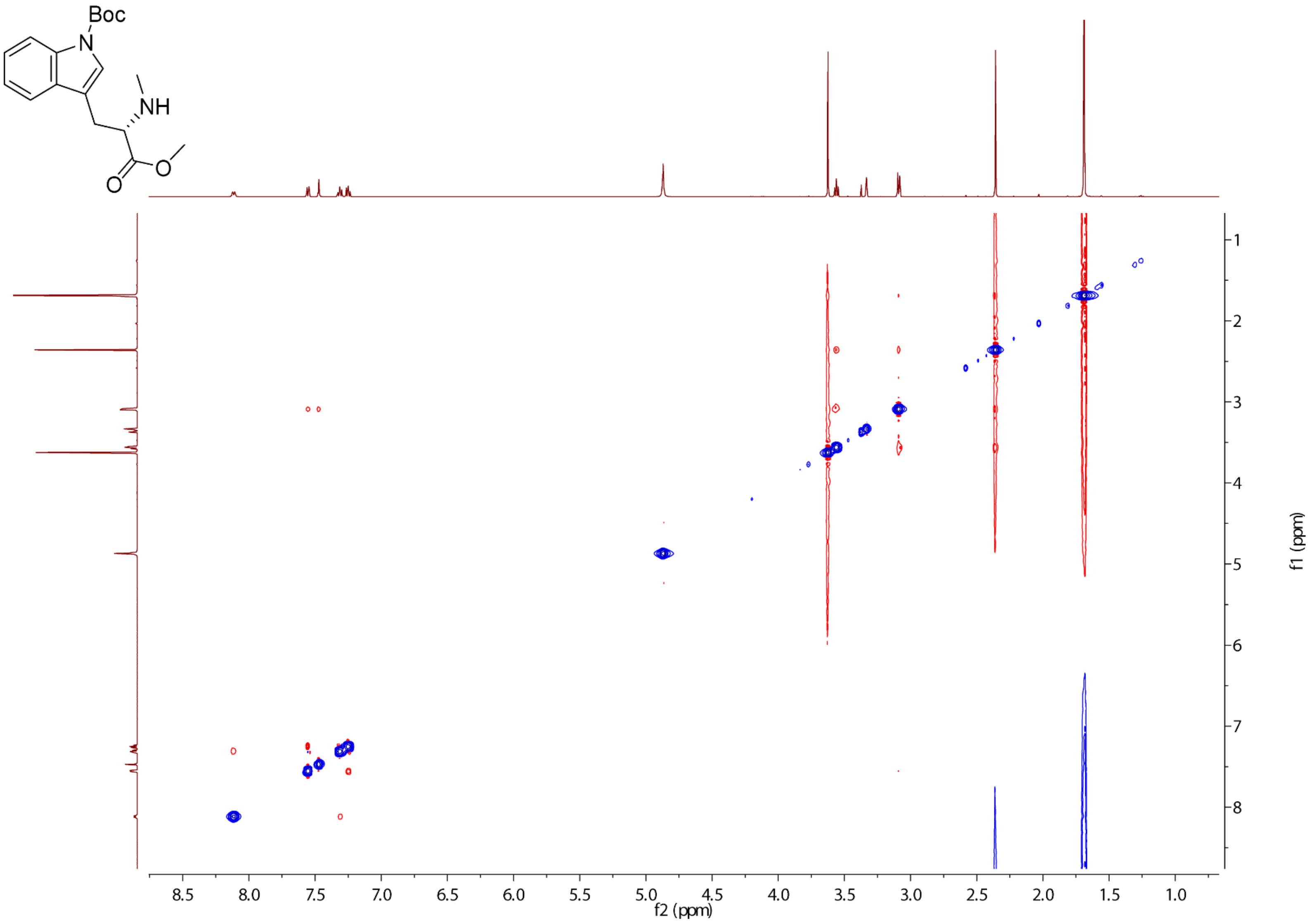

Figure S35. NOESY spectrum (CD_3OD) of **12** .

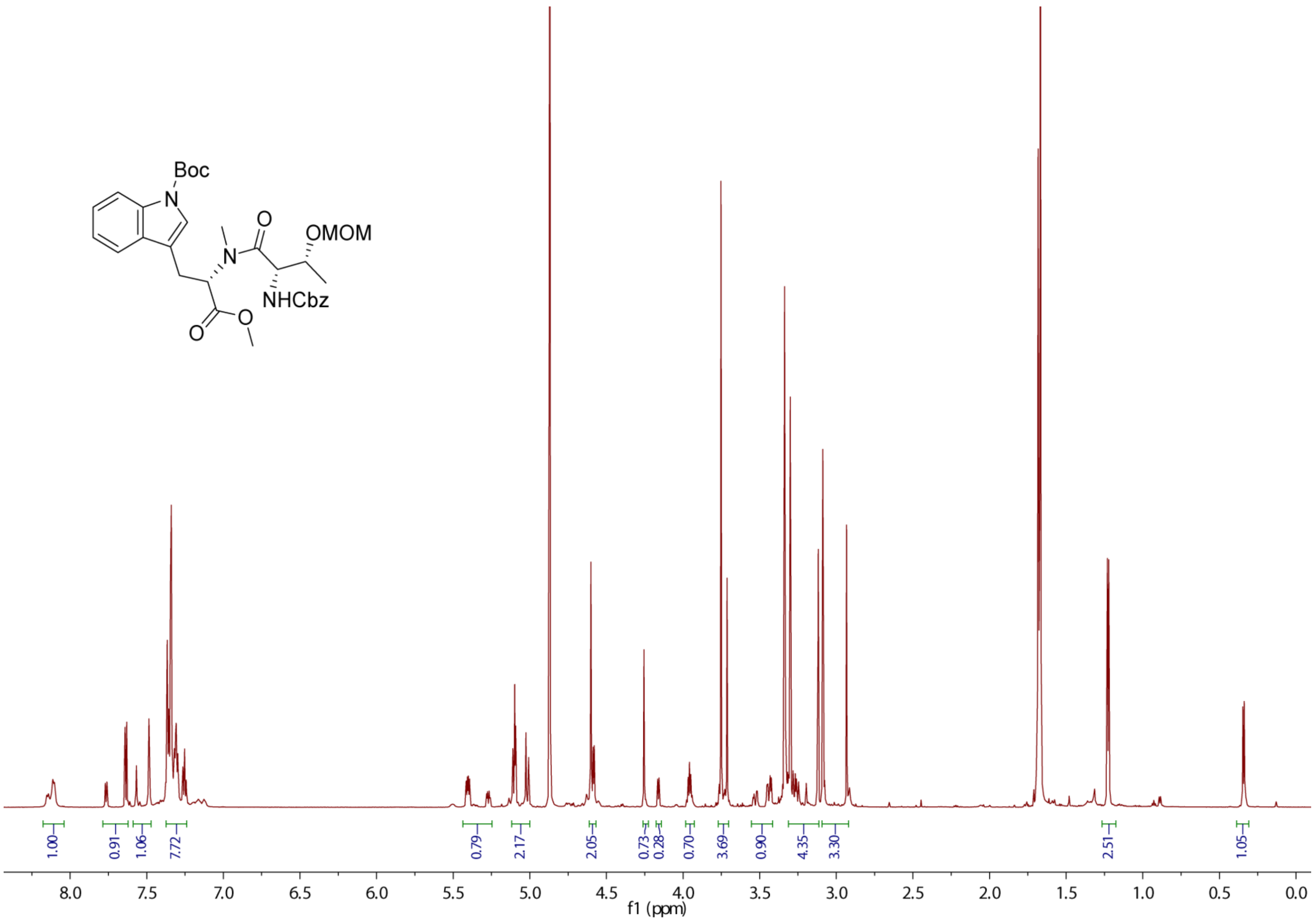

Figure S36. ^{1}H NMR spectrum (700 MHz, CD_3OD) of **13**.

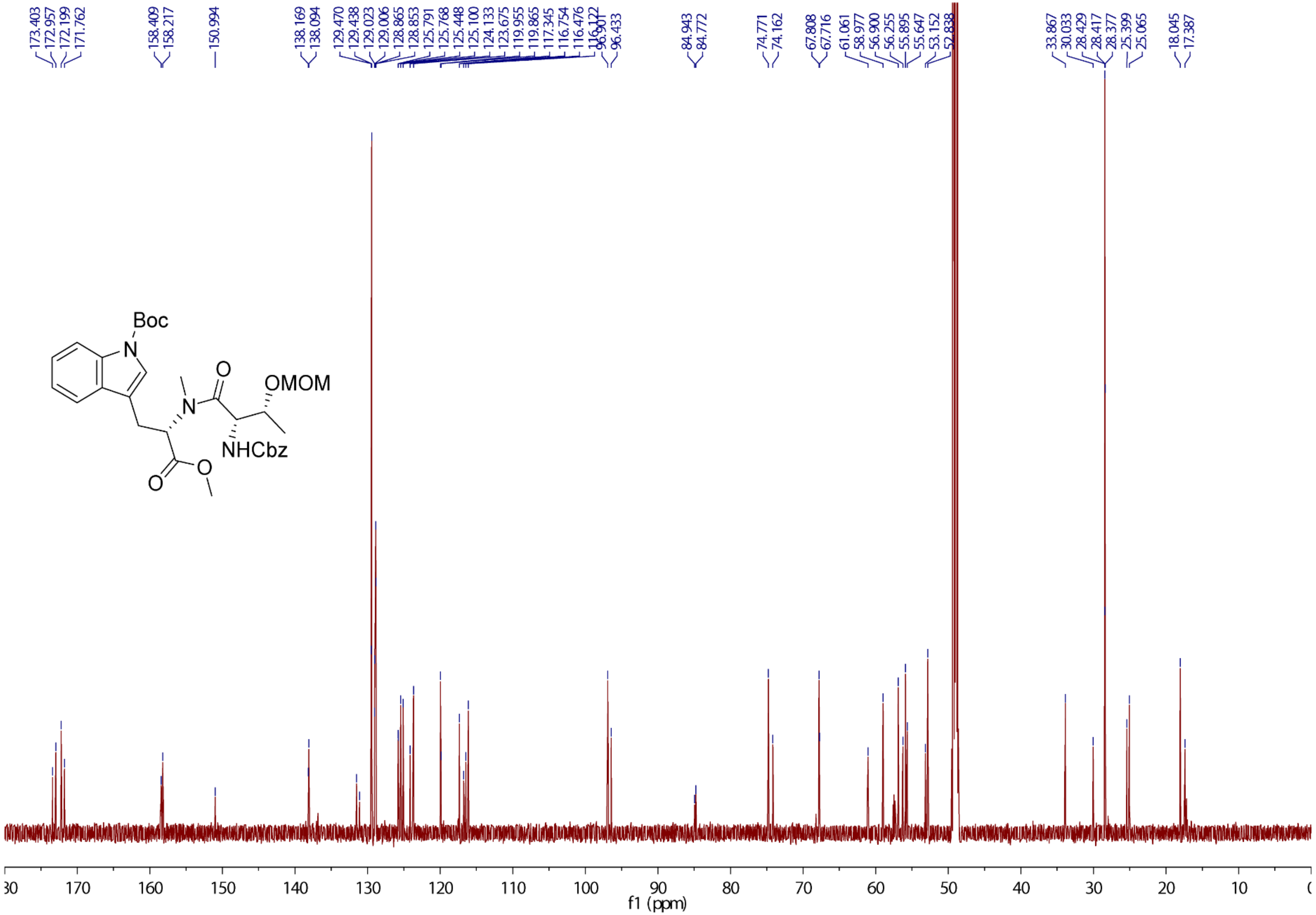

Figure S37. ^{13}C NMR spectrum (176 MHz, CD_3OD) of **13**.

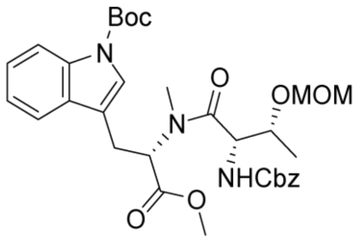

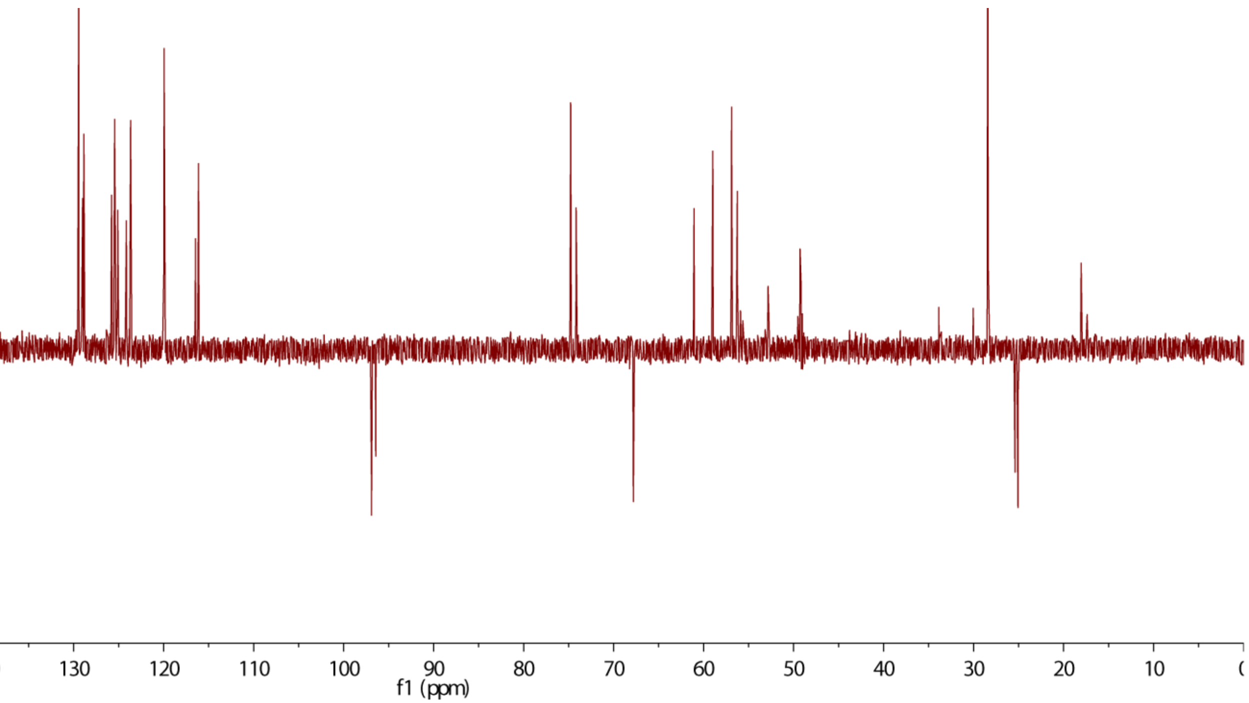

Figure S38. ^{13}C-DEPT-135 NMR spectrum (176 MHz, CD_3OD) of **13**.

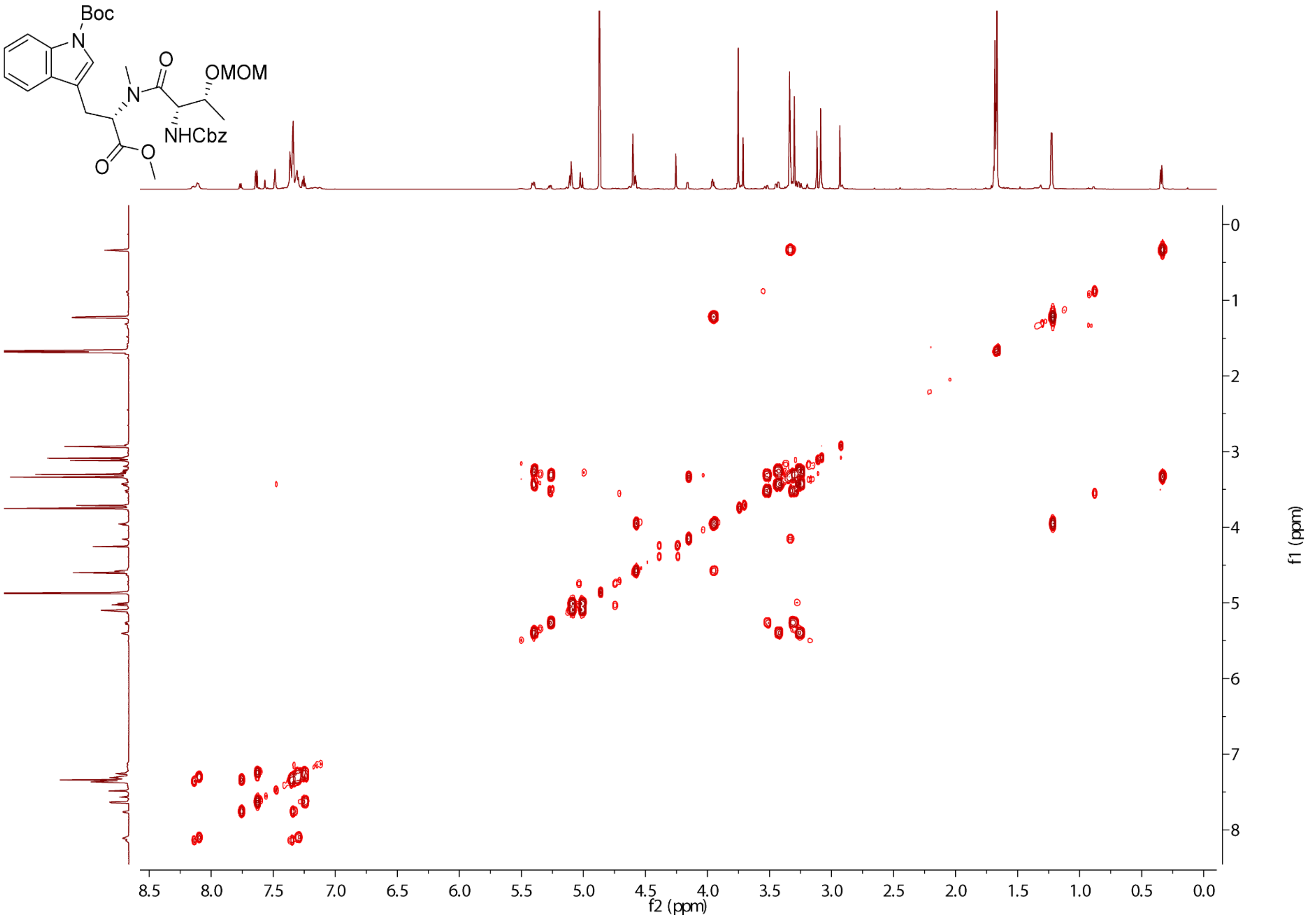

Figure S39. ^{1}H-^{1}H COSY spectrum (700 MHz, CD_3OD) of **13**.

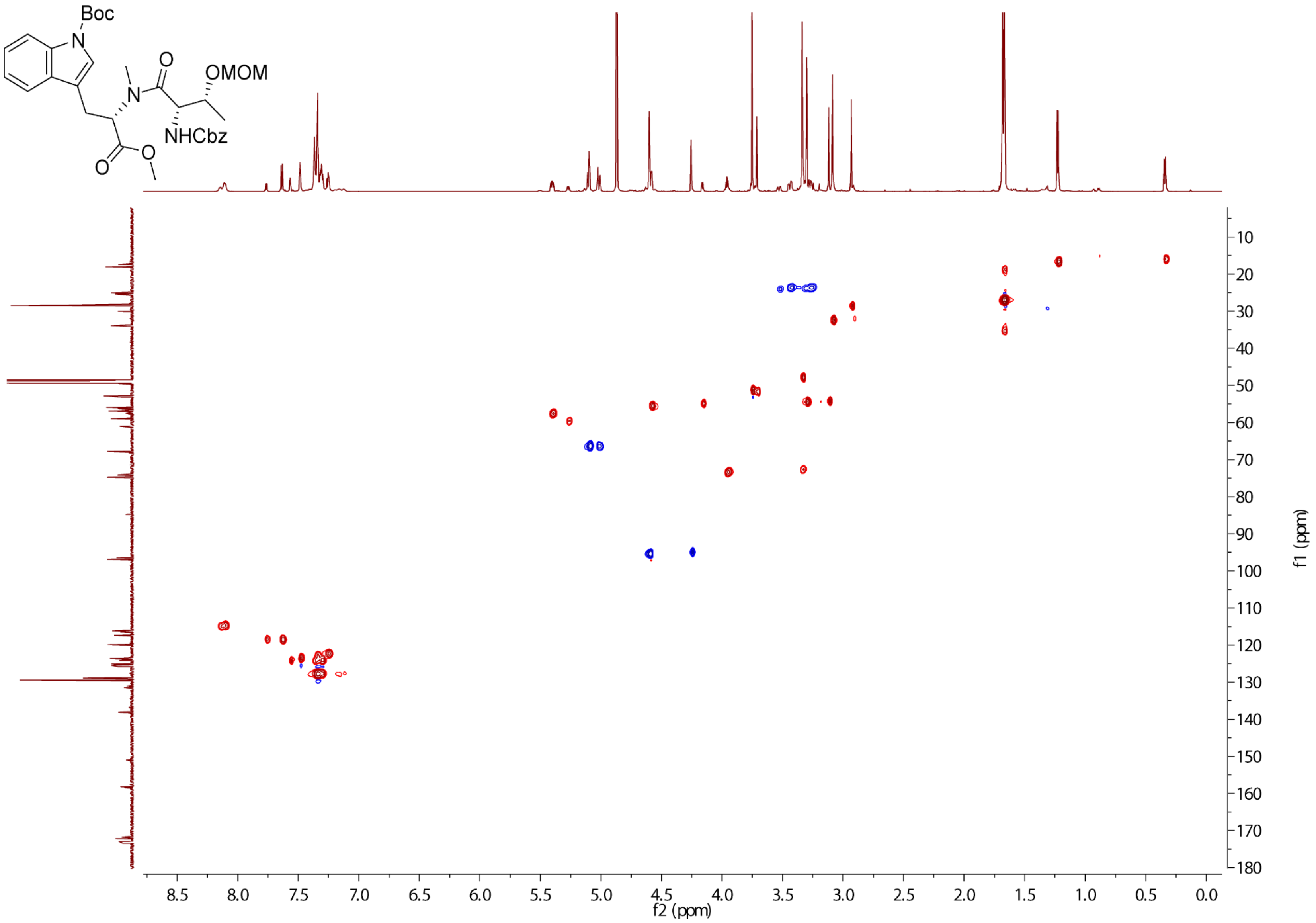

Figure S40. HSQC spectrum (CD_3OD) of **13**.

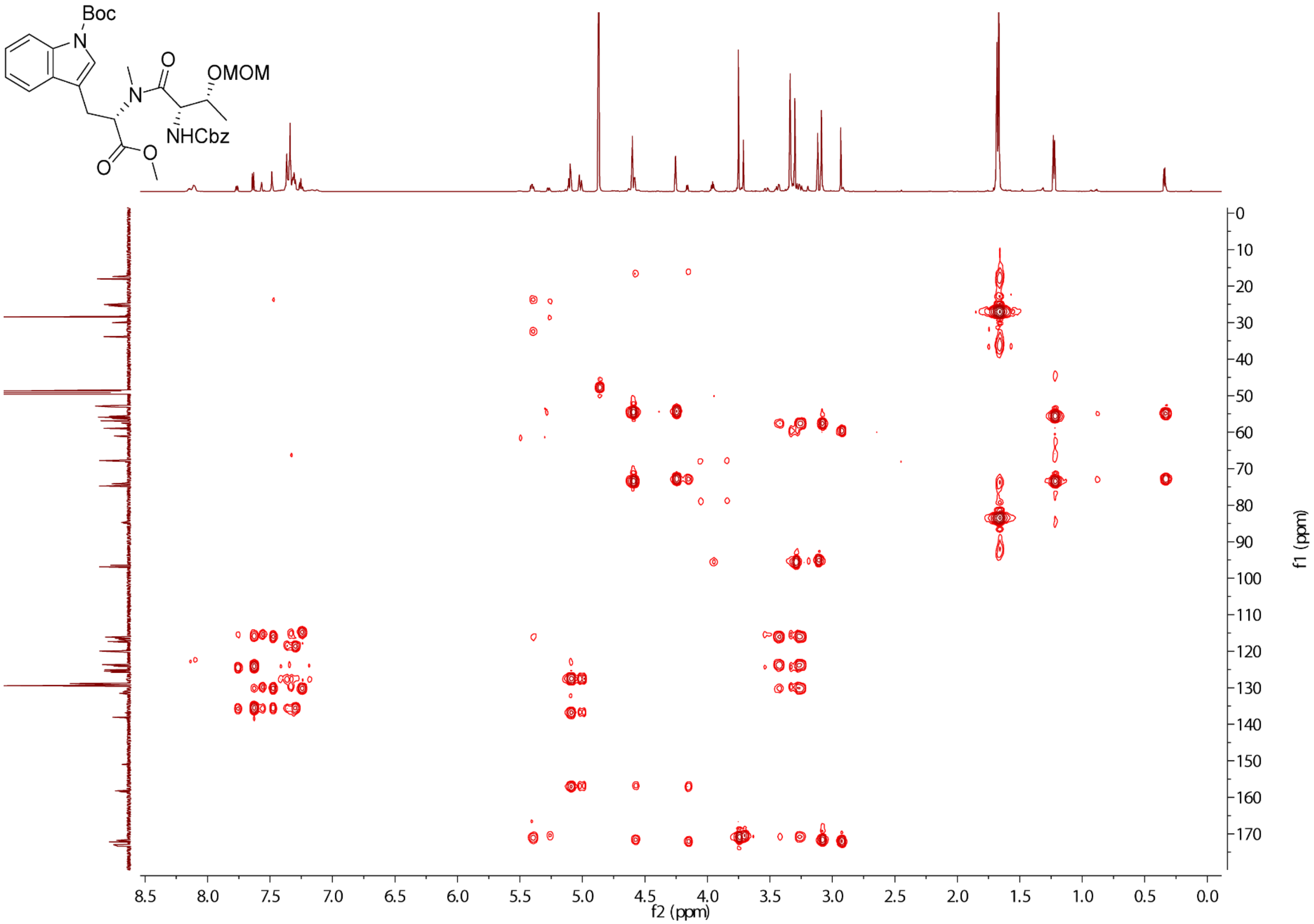

Figure S41. HMBC spectrum (CD_3OD) of **13**.

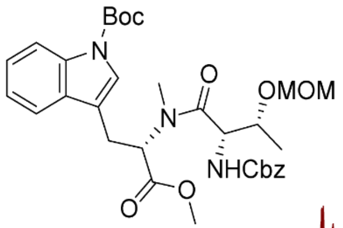

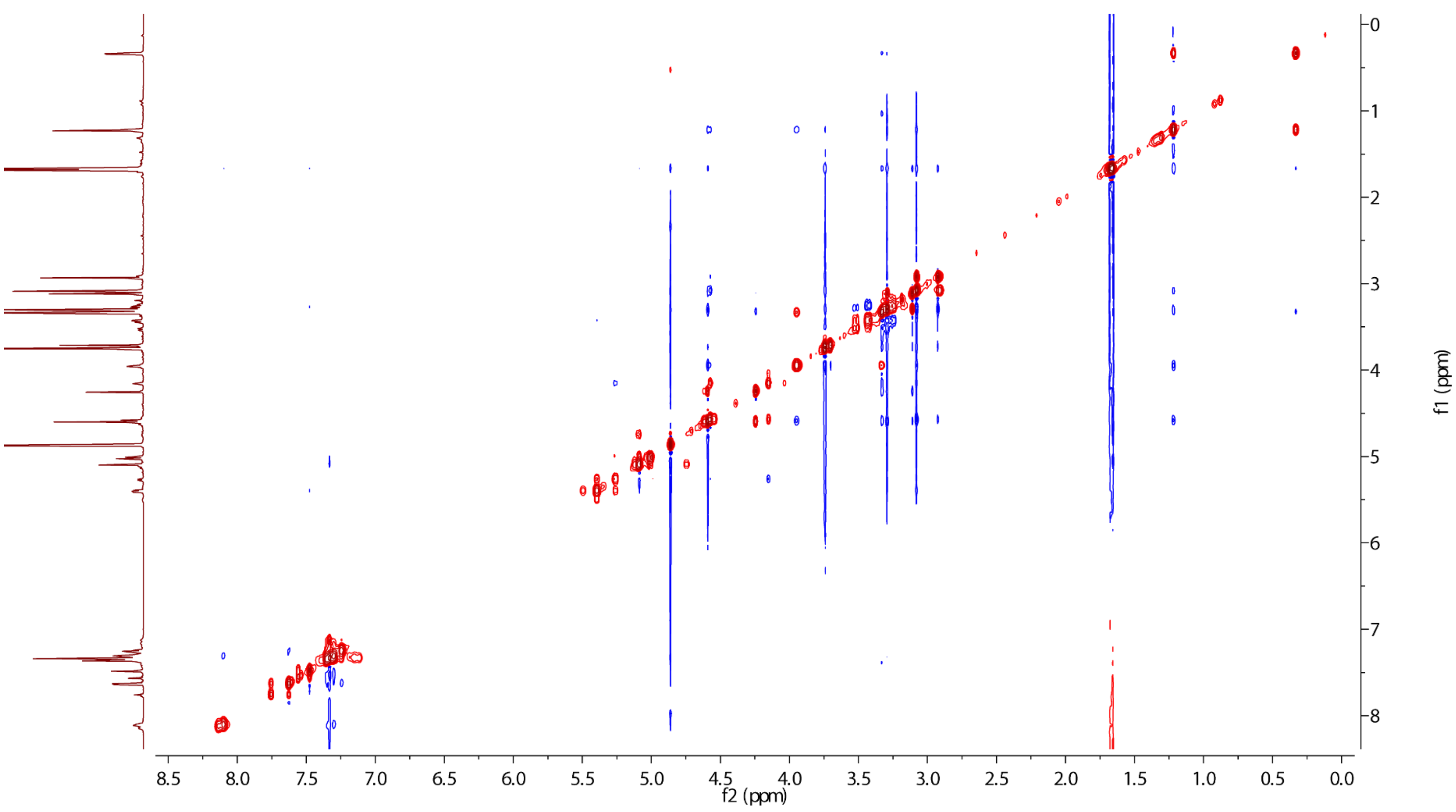

Figure S42. NOESY spectrum (CD_3OD) of **13**.

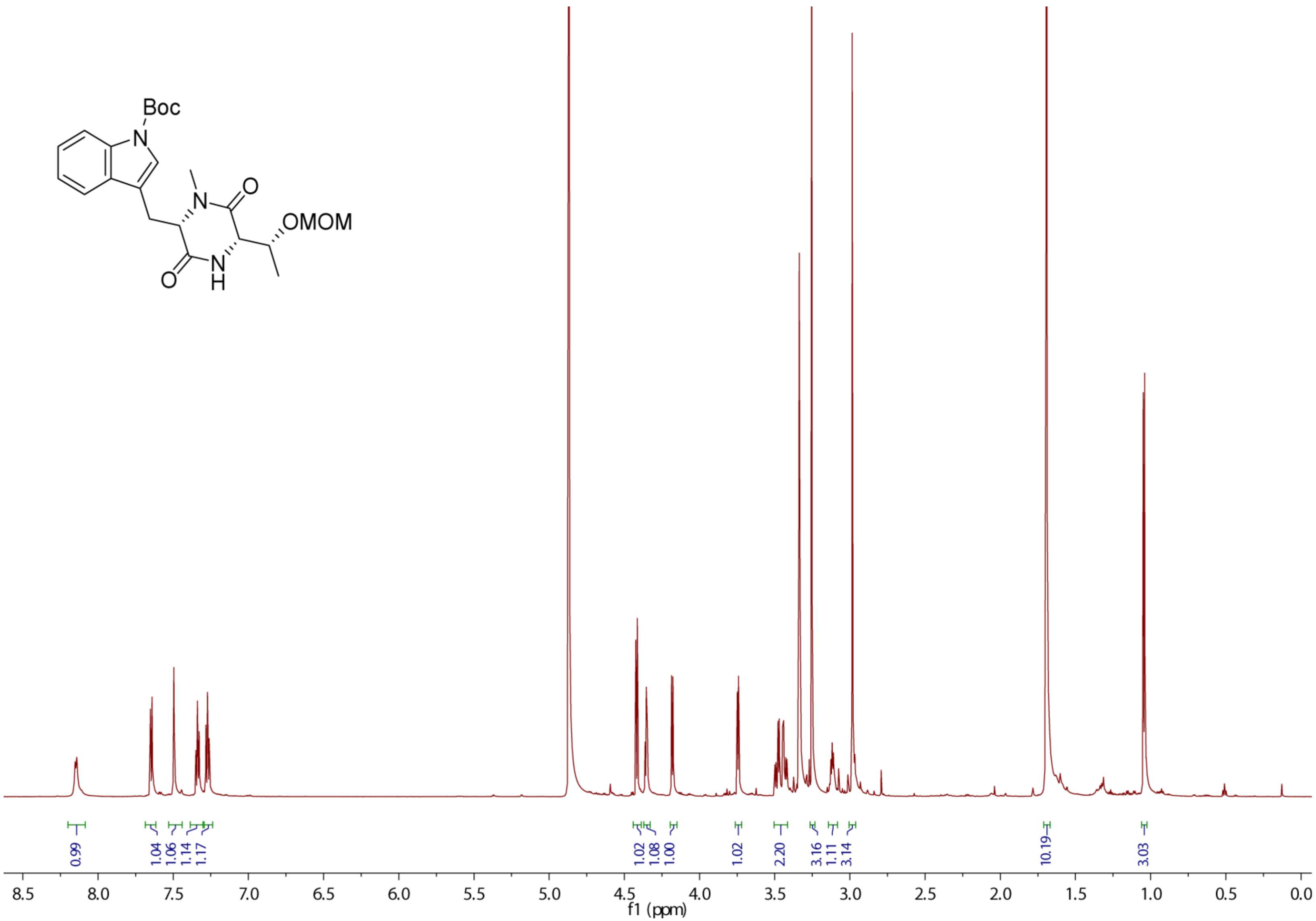

Figure S43. ^{1}H NMR spectrum (700 MHz, CD_3OD) of **14**.

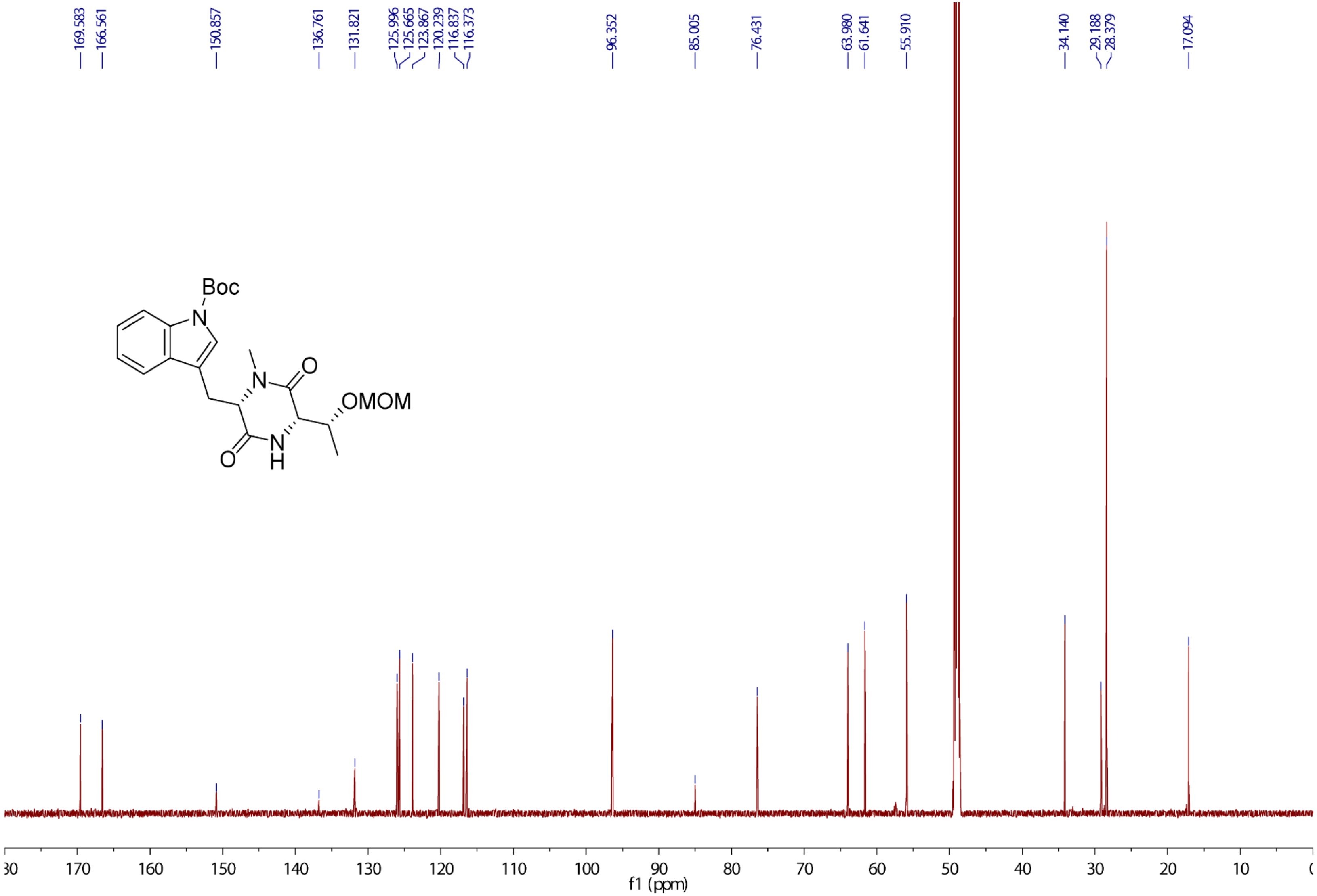

Figure S44. ^{13}C NMR spectrum (176 MHz, CD_3OD) of **14**.

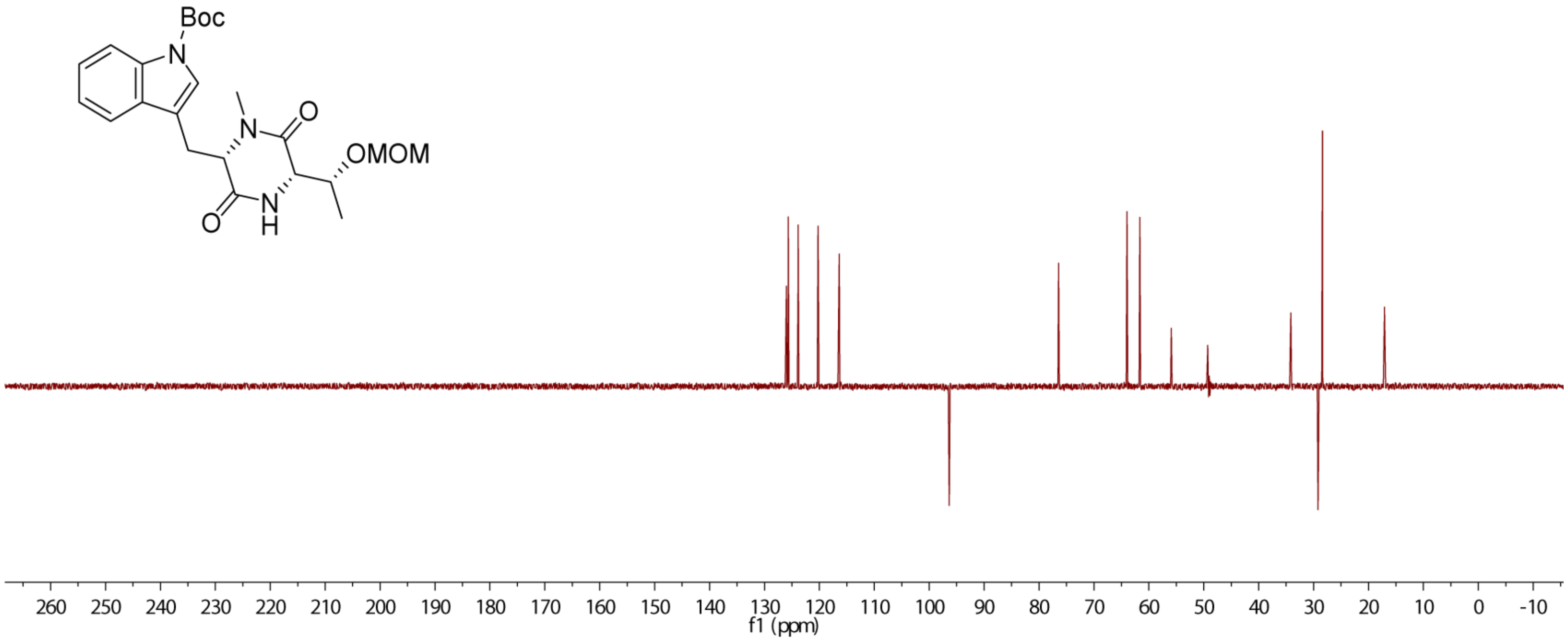

Figure S45. ^{13}C-DEPT-135 NMR spectrum (176 MHz, CD_3OD) of **14**.

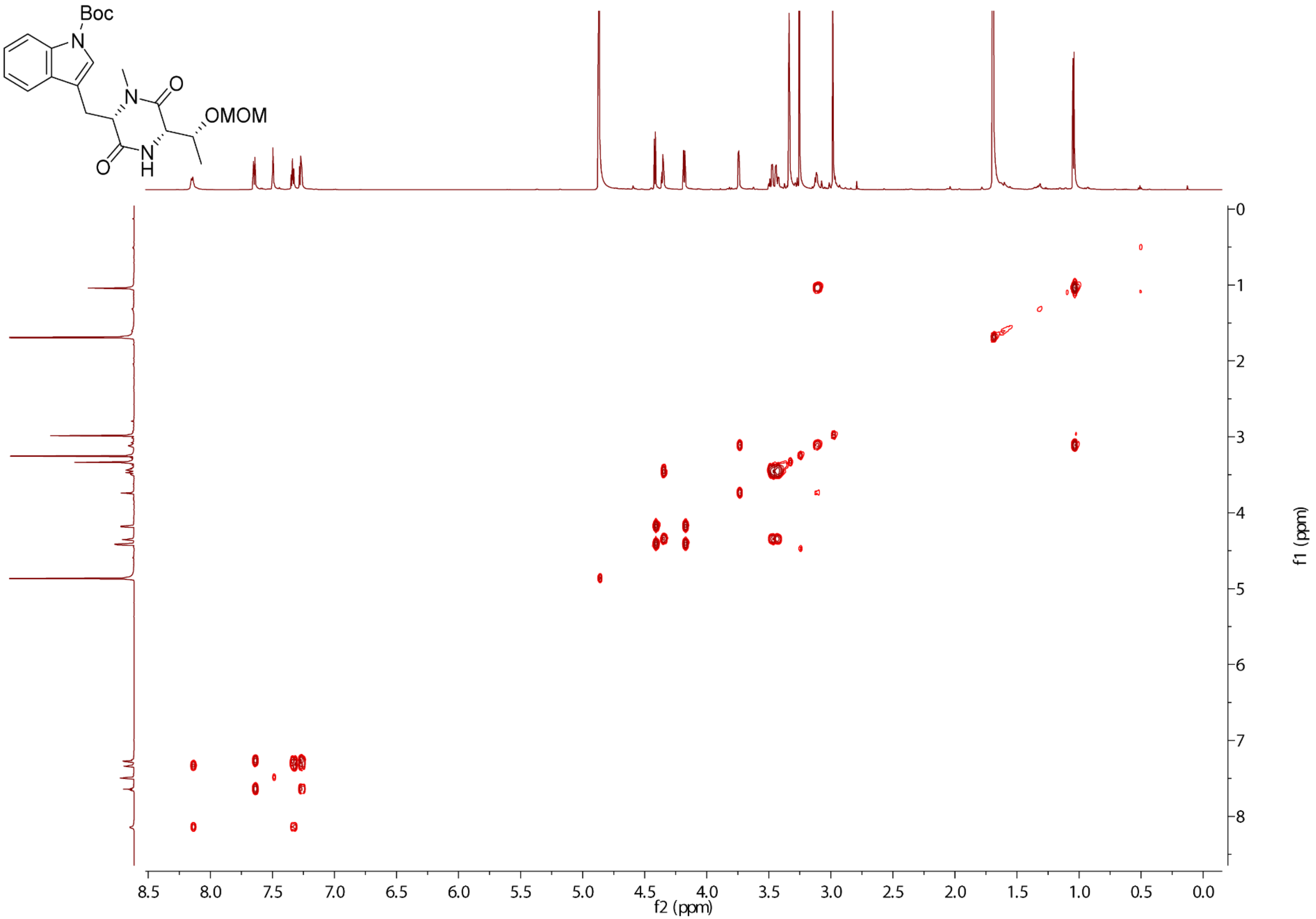

Figure S46. ^1H-^1H COSY spectrum (700 MHz, CD_3OD) of **14**.

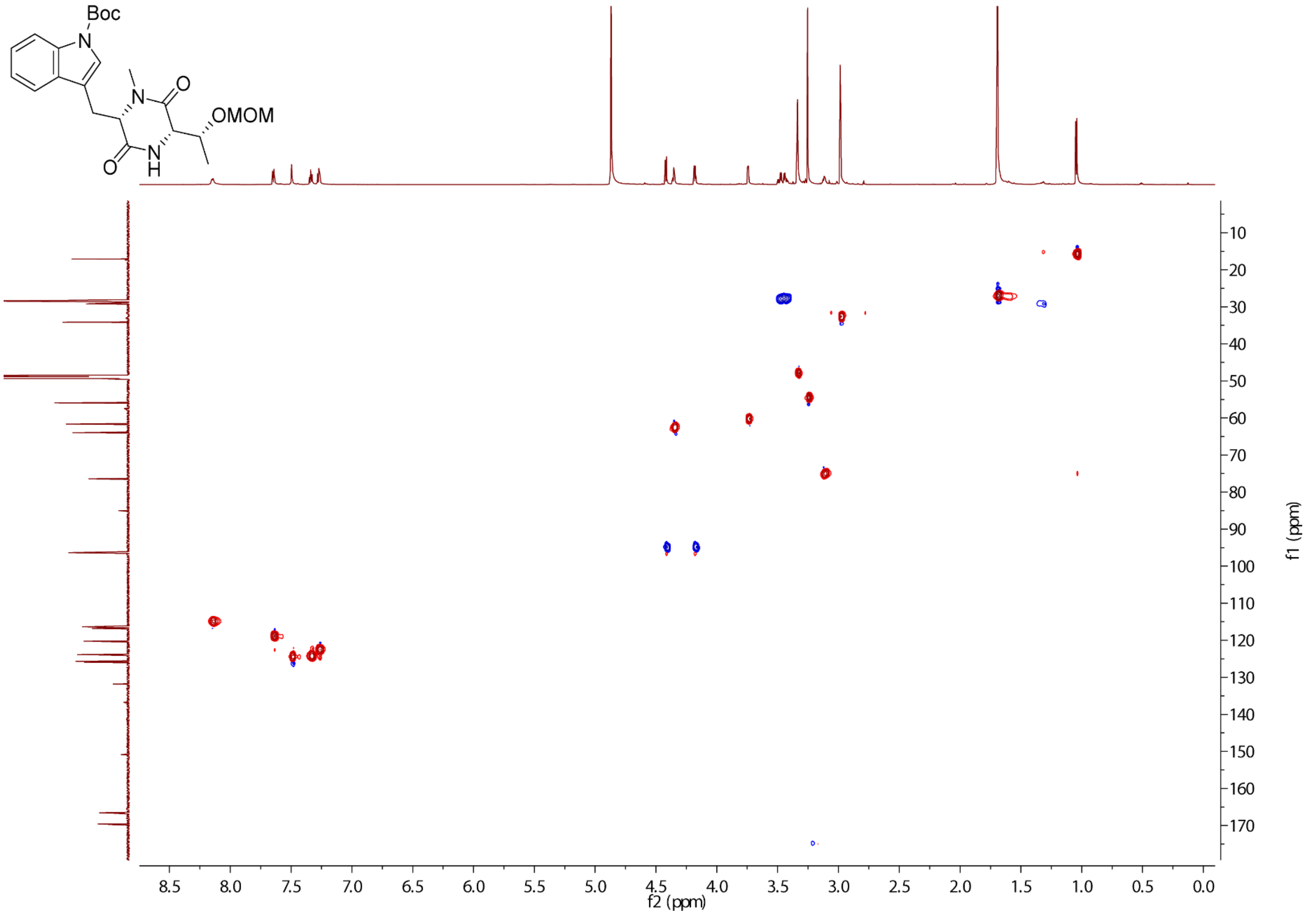

Figure S47. HSQC spectrum (CD_3OD) of **14**.

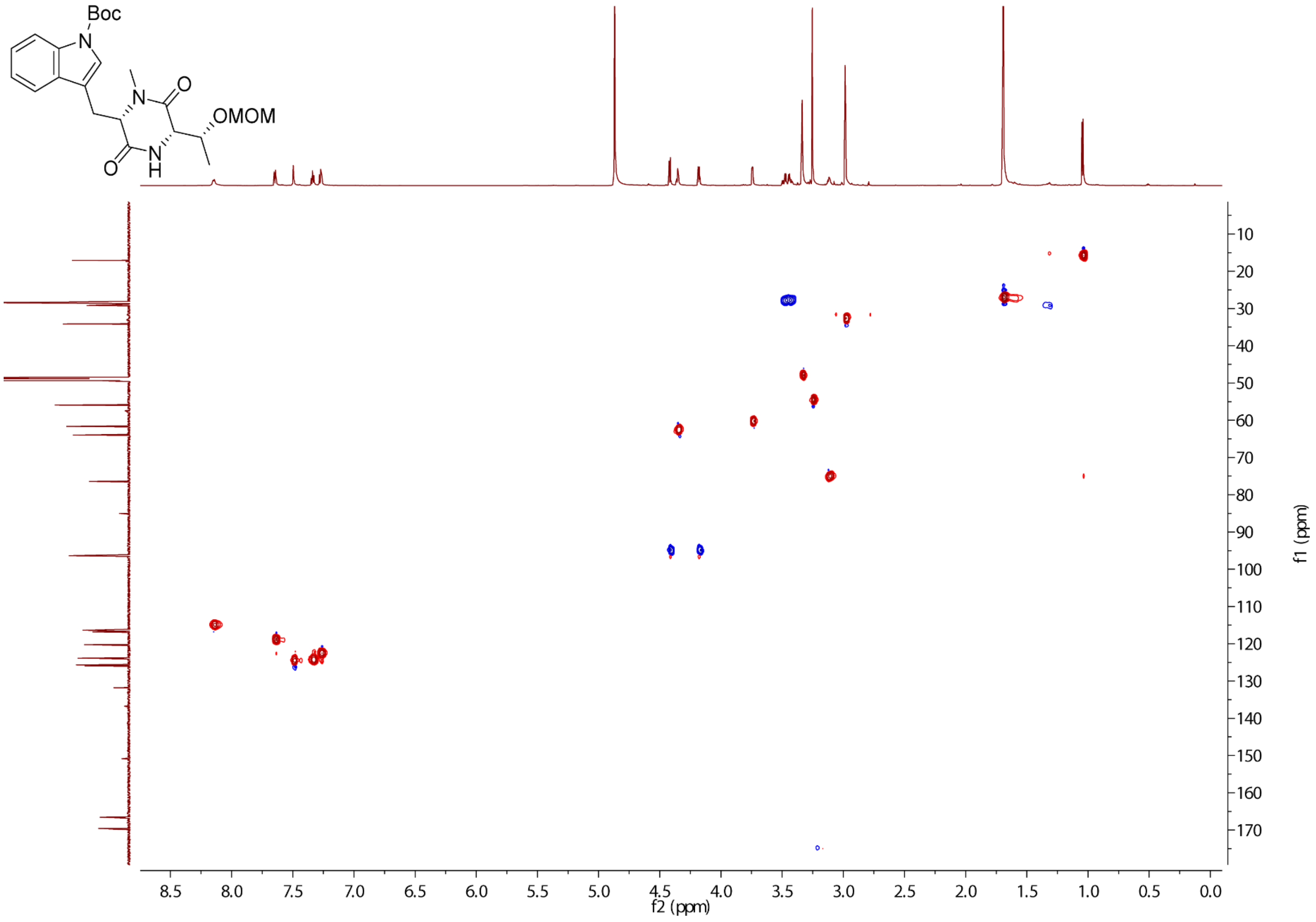

Figure S48. HMBC spectrum (CD_3OD) of **14**.

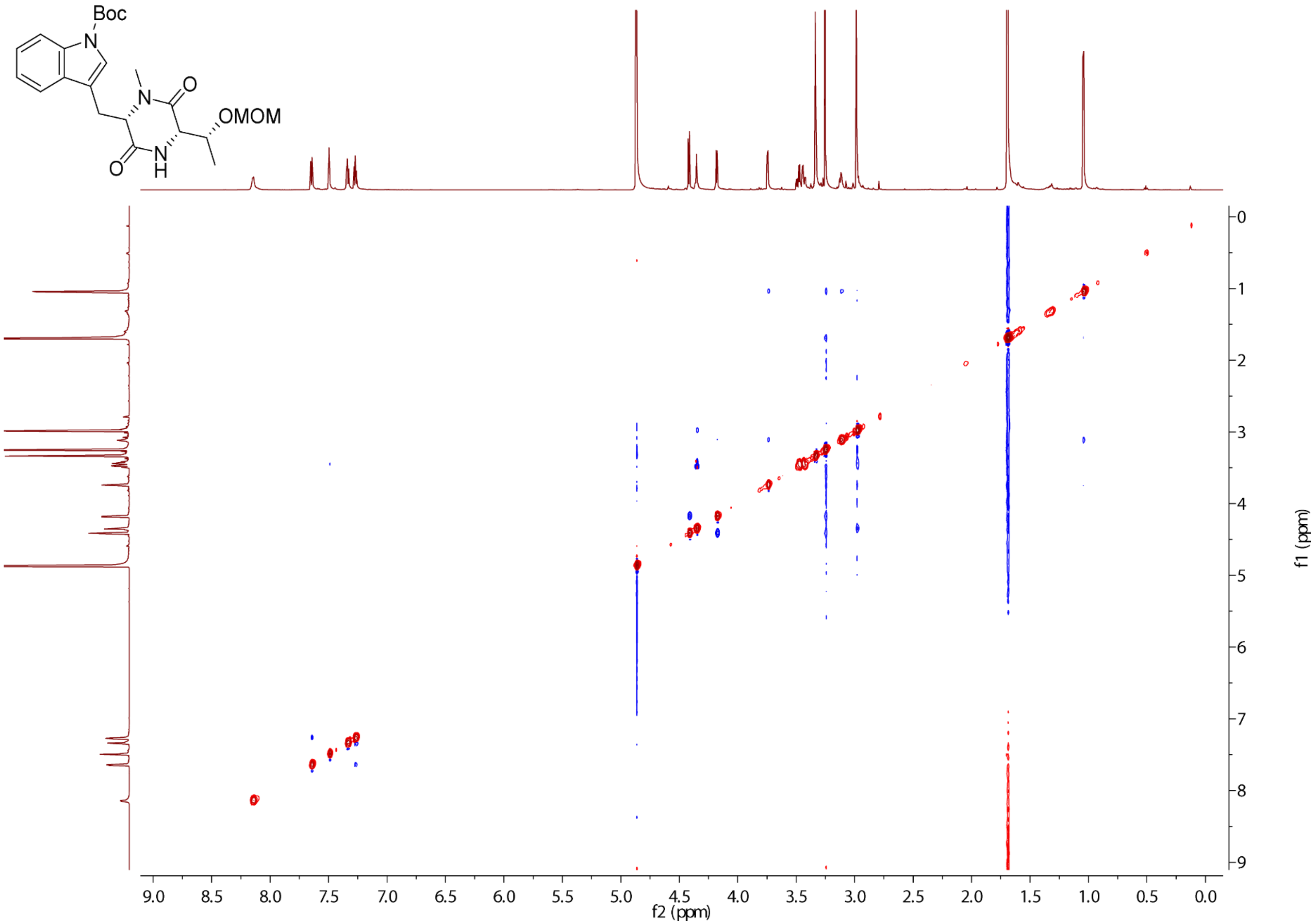

Figure S49. NOESY spectrum (CD_3OD) of **14**.

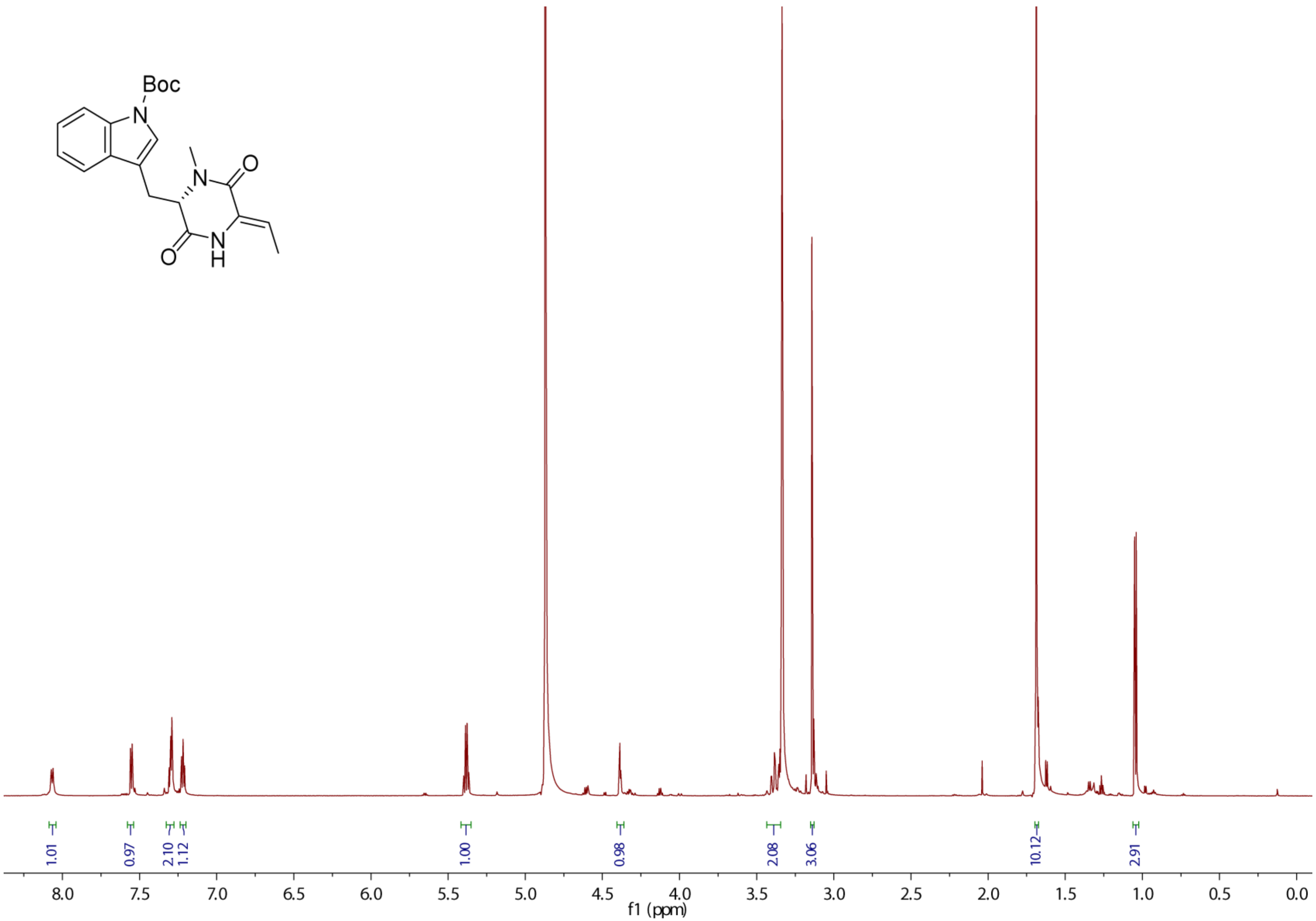

Figure S50. ^{1}H NMR spectrum (700 MHz, CD_3OD) of **15**.

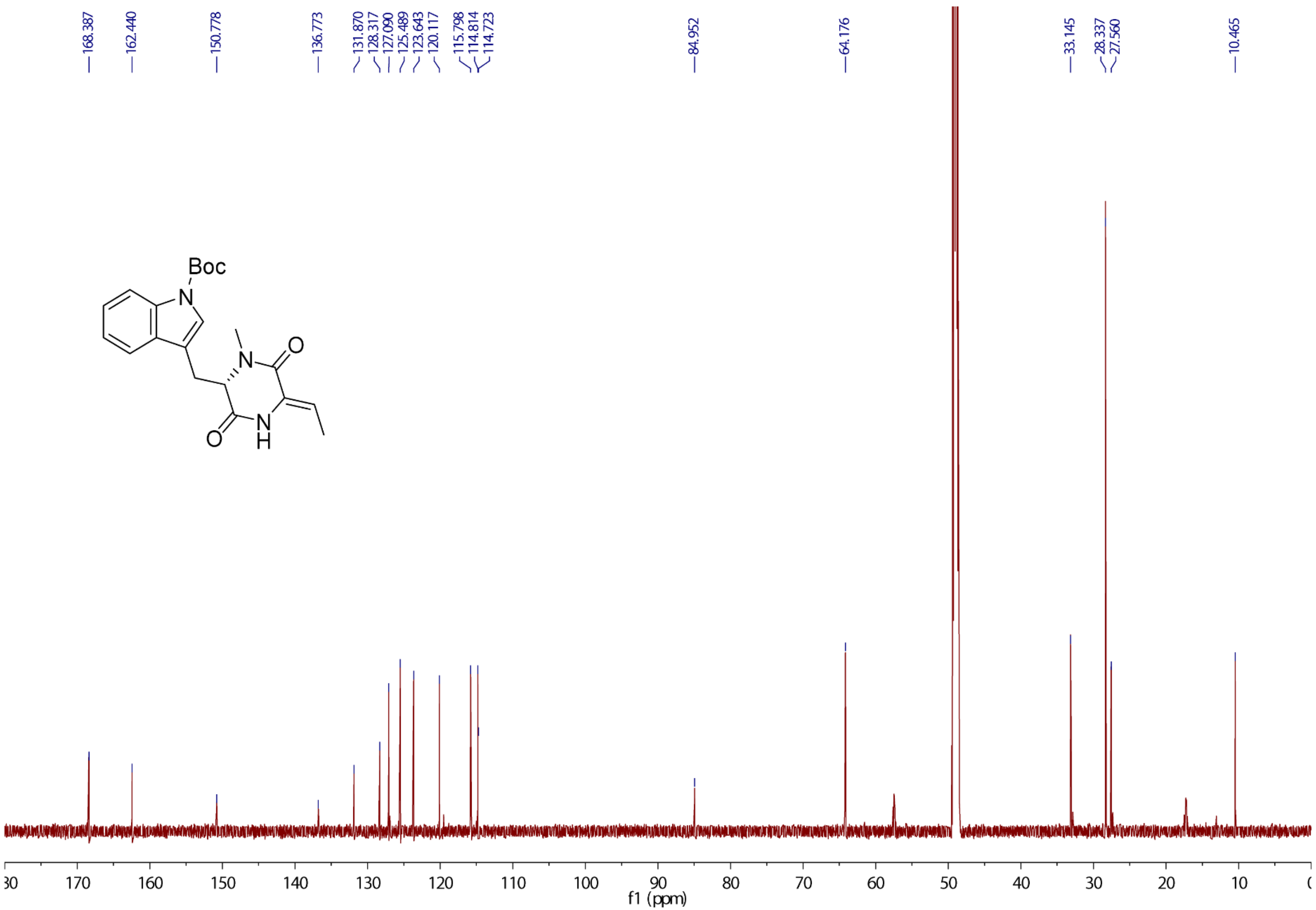

Figure S51. ^{13}C NMR spectrum (176 MHz, CD_3OD) of **15**.

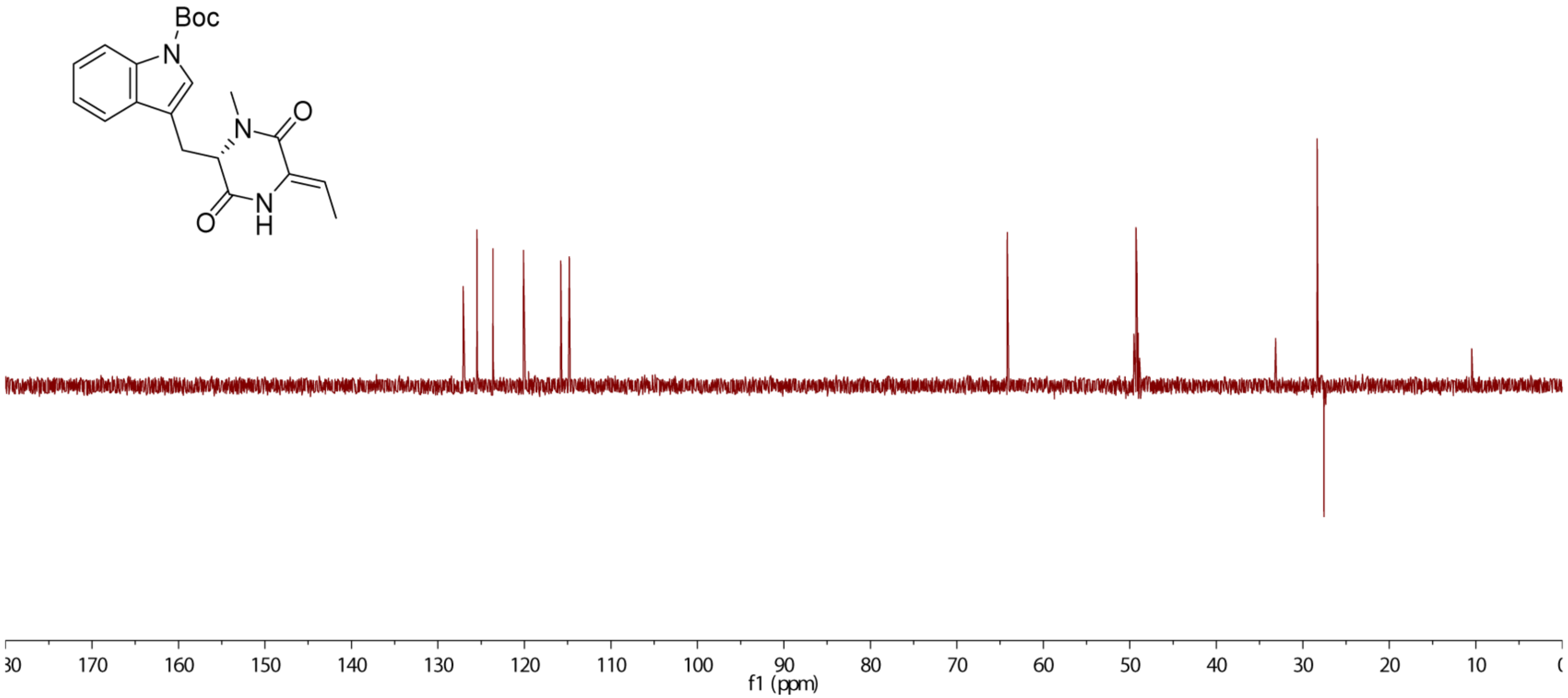

Figure S52. ^{13}C-DEPT-135 NMR spectrum (176 MHz, CD_3OD) of **15**.

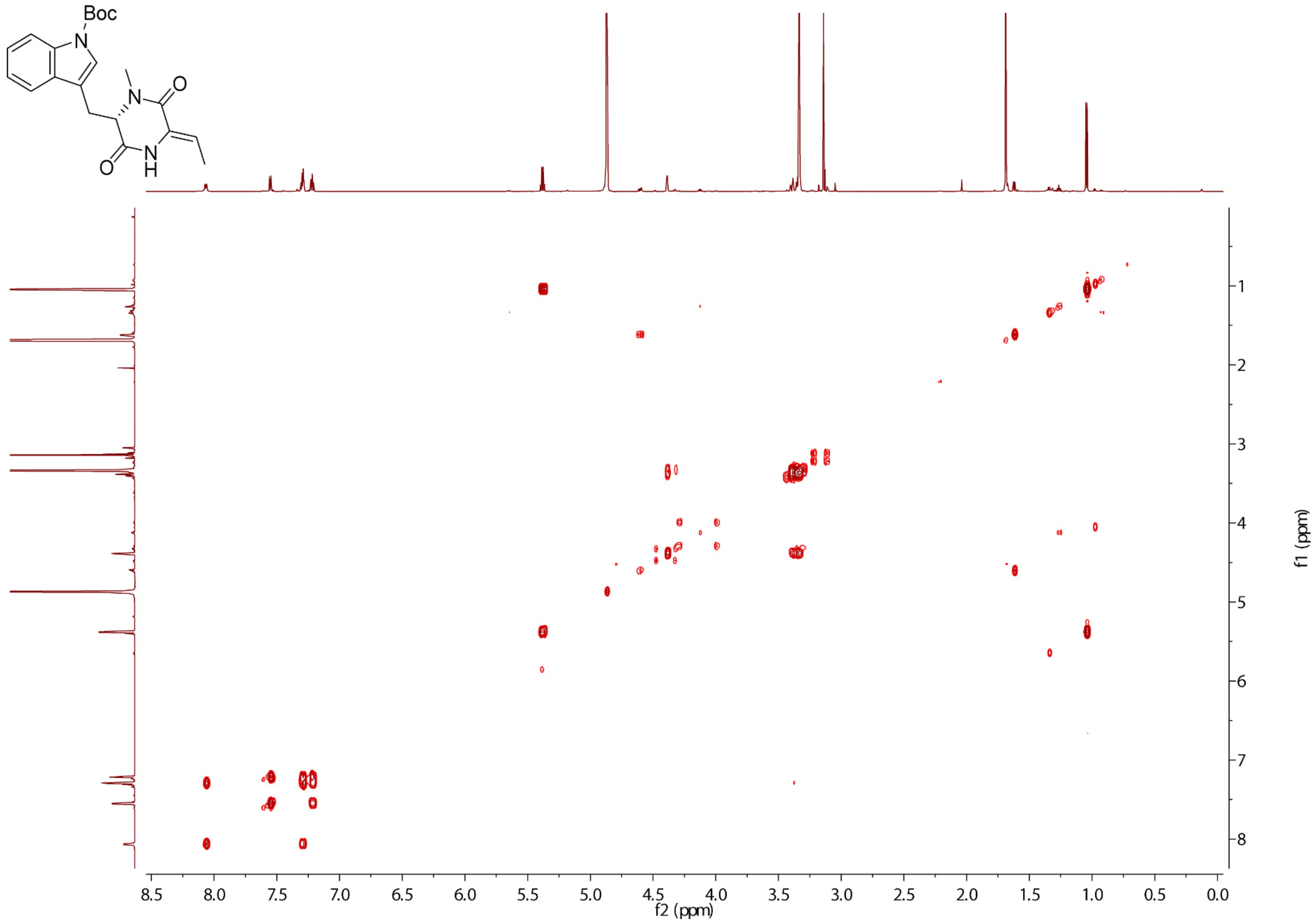

Figure S53. ^{1}H-^{1}H COSY spectrum (700 MHz, CD_3OD) of **15**.

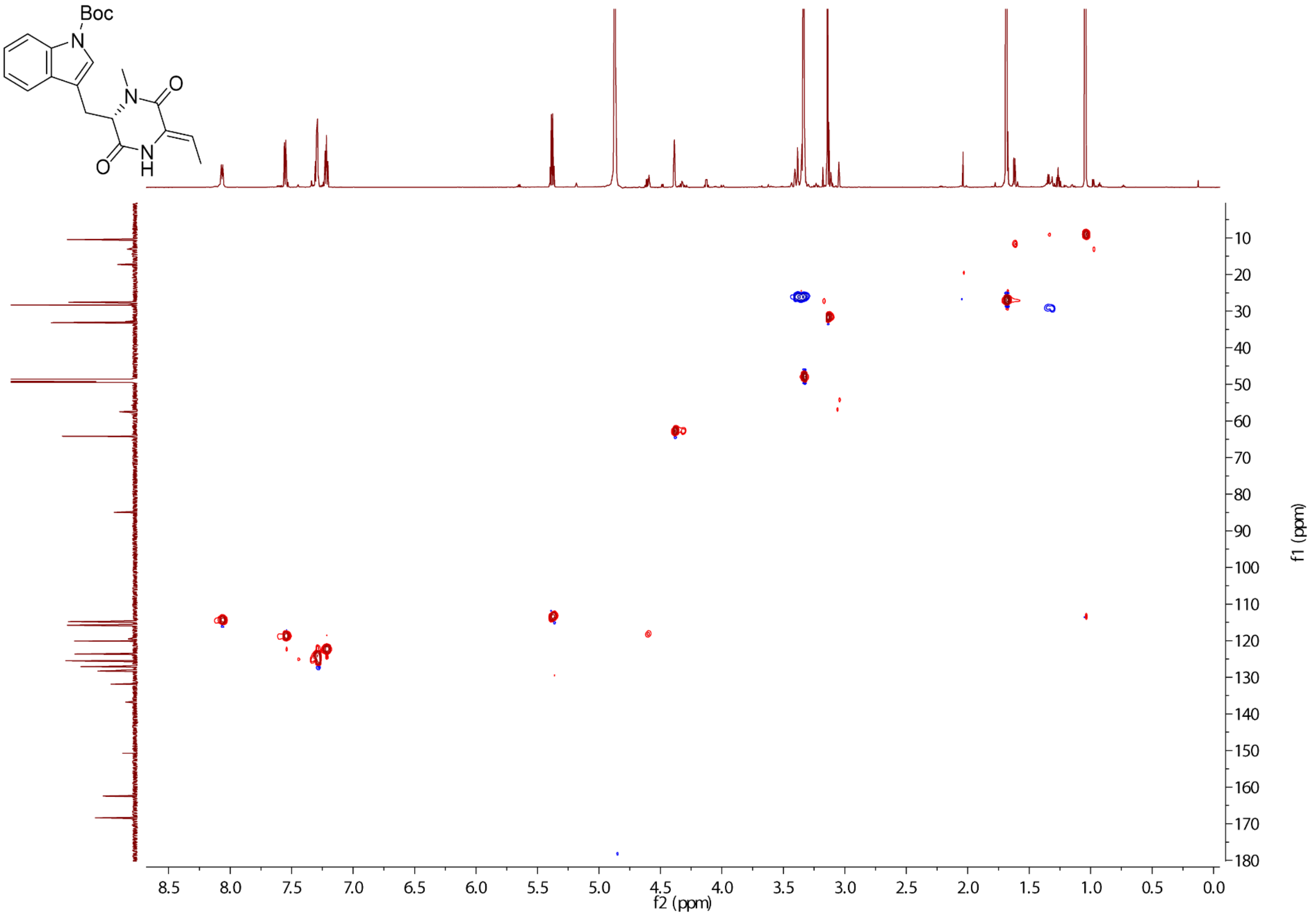

Figure S54. HSQC spectrum (CD_3OD) of **15**.

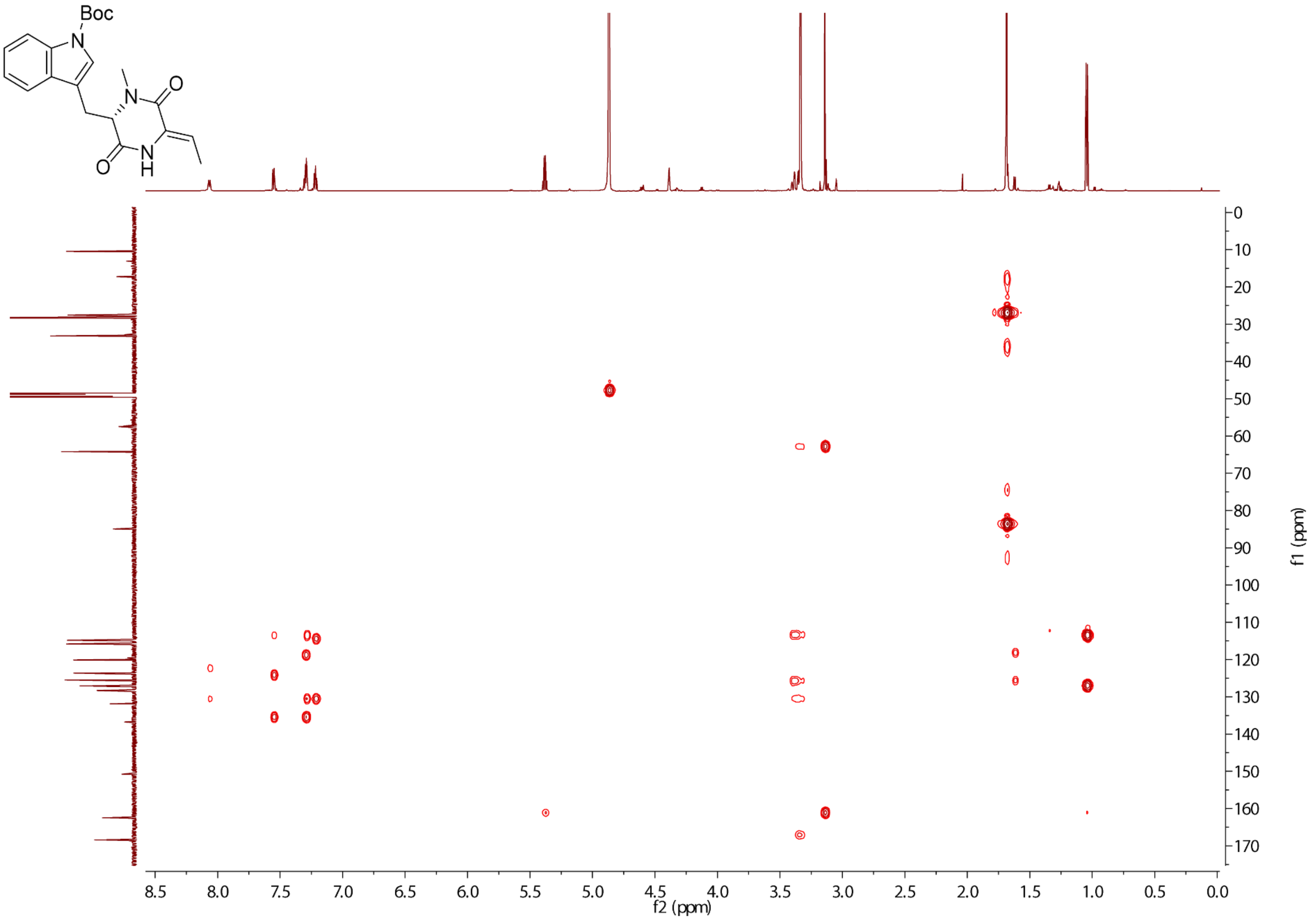

Figure S55. HMBC spectrum (CD_3OD) of **15**.

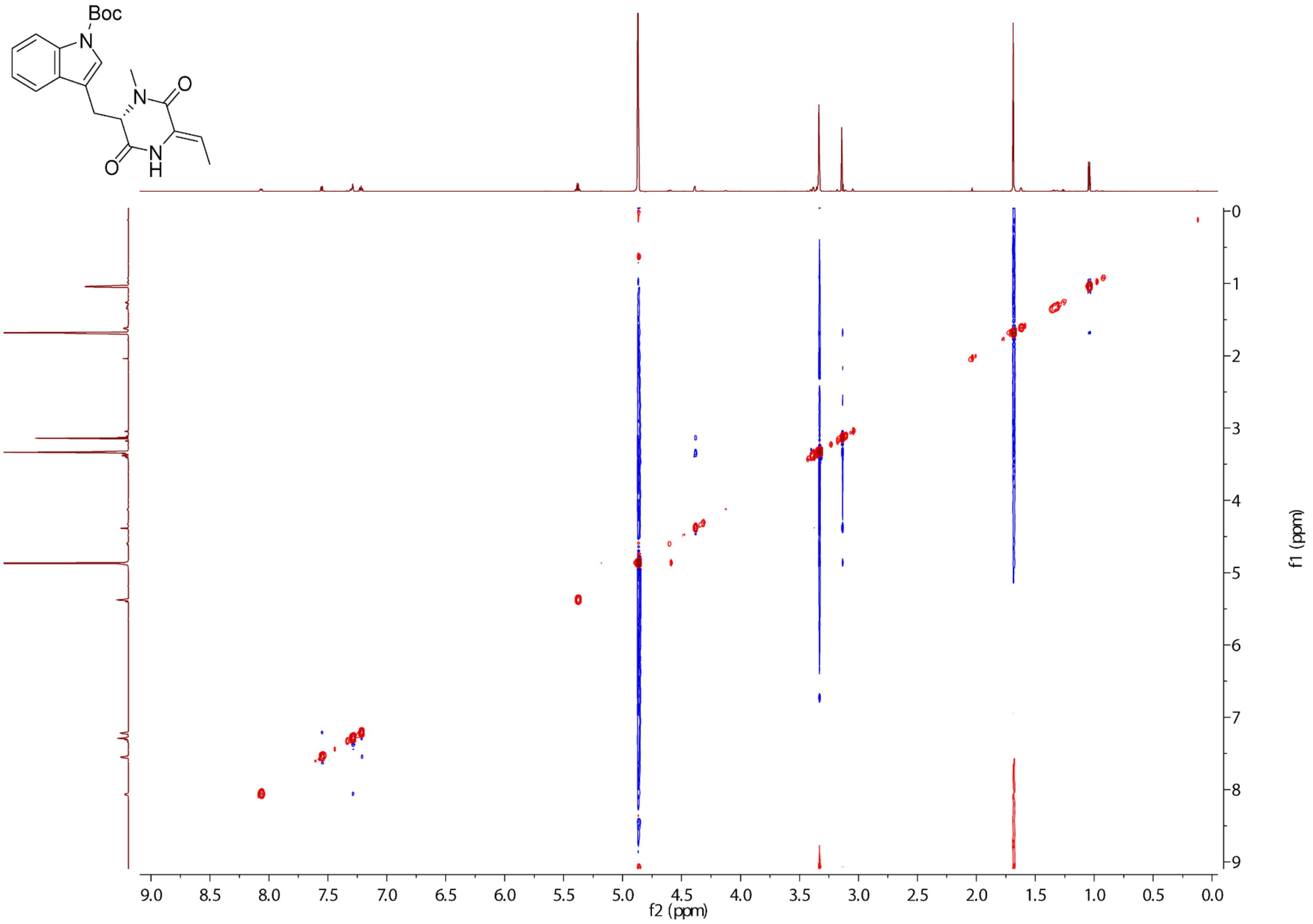

Figure S56. NOESY spectrum (CD_3OD) of **15**.

References

1. White, K. N.; Konopelski, J. P. *Org. Lett.,* **2005,** *7,* 4111–4112.
2. Blessing, R. H. *Acta Cryst. A,* **1995,** *51,* 33–36.
3. Sheldrick, G. M. *Acta Cryst. A*, **2015,** *71,* 3–8.
4. Sheldrick, G. M. *Acta Cryst. A,* **2008,** *64,* 112–122.
5. Patel, J. B.; Cockerill, F. R.; Bradford, P. A.; Eliopoulos, G. M.; Hindler, J. A.; Jenkins, S. G.; Lewis, J. S.; Limbago, B.; Miller, L. A.; Nicolau, D. P.; Powell, M.; Swenson, J. M.; Turnidge, J. D.; Weinstein, M. P.; Zimmer, B. L. Methods for dilution antimicrobial susceptibility tests for bacteria that grow aerobically. Approved Standard, 10th Edition, vol 35, Clinical and Laboratory Standards Institute, USA, **2015**.
6. Tacconelli, E.; Carrara, E.; Savoldi, A.; Harbarth, S.; Mendelson, M.; Monnet, D. L.; Pulcini, C.; Kahlmeter, G.; Kluytmans, J.; Carmeli, Y.; Ouellette, M.; Outterson, K.; Patel, J.; Cavaleri, M.; Cox, E. M.; Houchens, C. R.; Grayson, M. L.; Hansen, P.; Singh, N.; Theuretzbacher, U.; Magrini, N. *Lancet Infect. Dis.,* **2018,** *18,* 318–327.
7. Woodford, N.; Adebiyi, A. M.; Palepou, M. F.; Cookson, B. D. *Antimicrob. Agents Chemother.,* **1998,** *42,* 502–508.
8. Anagnostopoulos, C.; Spizizen, J. *J. Bacteriol.,* **1961,** *81,* 741–746.
9. Ma, D.; Cook, D. N.; Alberti, M.; Pon, N. G.; Nikaido, H.; Hearst, J. E. *Mol. Microbiol.,* **1995,** *16,* 45–55.
10. Srikumar, R.; Kon, T.; Gotoh, N.; Poole, K. *Antimicrob. Agents Chemother.,* **1998,** *42,* 65–71.
